# Forecasting Causal Effects of Interventions versus Predicting Future Outcomes

**DOI:** 10.1080/10705511.2020.1780598

**Published:** 2020-09-08

**Authors:** Christian Gische, Stephen G. West, Manuel C. Voelkle

**Affiliations:** 1 Humboldt-Universität zu Berlin, Germany;; 2 Arizona State University, Arizona;; 3 Max Planck Institute for Human Development, Germany

**Keywords:** Causal inference, DAG, cross-lagged panel, structural equation modeling

## Abstract

The present article provides a didactic presentation and extension of selected features of Pearl’s DAG-based approach to causal inference for researchers familiar with structural equation modeling. We illustrate key concepts using a cross-lagged panel design. We distinguish between (a) forecasts of the value of an outcome variable after an intervention and (b) predictions of future values of an outcome variable. We consider the mean level and variance of the outcome variable as well as the probability that the outcome will fall within an acceptable range. We extend this basic approach to include additive random effects, allowing us to distinguish between average effects of interventions and person-specific effects of interventions. We derive optimal person-specific treatment levels and show that optimal treatment levels may differ across individuals. We present worked examples using simulated data based on the results of a prior empirical study of the relationship between blood insulin and glucose levels.

## Introduction

Psychologists have long distinguished between experimental designs in which participants are randomly assigned to an active treatment or a control treatment and passive observational designs (i.e., non-experimental designs without a manipulation) in which the responses of participants are simply observed (e.g., Cronbach, 1957, 1975). Given randomization and manipulation of the treatment conditions, experimental designs permit strong causal inferences when their assumptions are met (Shadish et al., 2002; West & Thoemmes, 2010). A traditional view is that longitudinal passive observational designs only permit prediction of participants’ scores on the criterion variable at future time points and do not allow for causal conclusions.

In this paper, we present an approach that shows that under certain conditions longitudinal passive observational designs contain information that allows researchers to forecast the causal effect of a hypothetical treatment, even if that treatment has not yet been carried out. We consider the commonly used cross-lagged panel design in which two variables *X* and *Y* are each measured at a set of equally spaced measurement waves. Throughout we make a clear distinction between (a) the forecast of the causal effect of a (hypothetical) intervention on an outcome at a future time point and (b) the prediction of the individual’s outcome at a future time point. The machinery for making forecasts of causal effects of interventions is provided by Judea Pearl’s (2009) graphical approach to causal inference (see also Elwert, 2013). We provide a brief didactic presentation and illustration of the relevant key ideas of Pearl’s approach, showing the links to ideas from traditional path analysis familiar to readers of this journal.

Using this approach, we illustrate how to forecast the effect of an intervention on the level of an outcome variable at a future time point. We also show how to calculate the variance of the forecasted value and the probability that the outcome variable attains a value within a predefined acceptable range. We initially assume a population of homogeneous individuals and later extend the approach to consider potential individual differences in the mean level of each person’s time series data (unobserved heterogeneity). To facilitate a clear illustration of these ideas, we use as a running example a simple system with two variables measured at *T* = 4 equally spaced time points. We assume that the data are generated in a dynamic system in which the value of *X* at time *t*, *X*_*t*_, influences the value of *Y* at time *t* + 1, *Y*_*t*+1_, and likewise *Y*_*t*_ influences *X*_*t*+1_. The system also includes autoregressive effects in which *X*_*t*_ influences *X*_*t*+1_ and *Y*_*t*_ influences *Y*_*t*+1_. In our illustration *X*_*t*_ represents blood insulin levels in non-diabetic adults at each of four equally spaced measurement waves measured in micro international units per milliliter (mcIU/ml). Blood glucose (*Y*_*t*_) is also measured at each measurement wave in milligrams per deciliter (mg/dl). For ease of presentation and without loss of generality, we use variables centered at the individual mean for each person.

**Figure 1.**
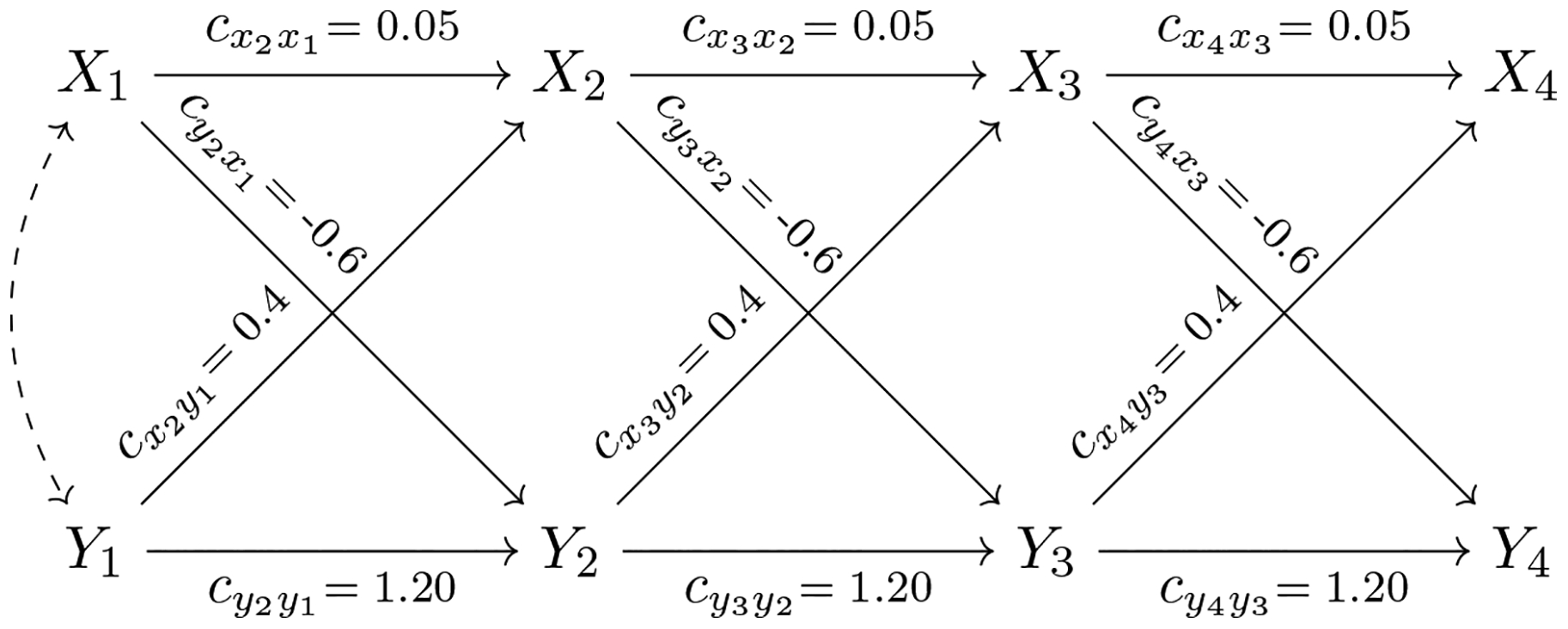
The causal diagram (DAG) of the causal model for the four-wave passive observational study is displayed. The dashed double-headed arrow (bidirected edge) represents a correlation between *X*_1_ on *Y*_1_ due to an unobserved common cause. Traditionally, in DAGs the disturbances denoted by $\varepsilon_{x_{t}}$, $\varepsilon_{y_{t}}$, *t* = 1, …, 4 which represent other unmeasured influences on each variable are not represented. Solid single-headed arrows (directed edges) are labeled with path coefficients that quantify direct causal effects. For example, the cross-lagged coefficient $c_{x_{2}y_{1}}$ represents the direct effect of *Y*_1_ on *X*_2_. The autoregressive coefficient $c_{x_{4}x_{3}}$ represents the direct effect of *X*_3_ on *X*_4_. The coefficients $c_{y_{t+1}x_{t}}$ and $c_{y_{t+1}y_{t}}$, *t* ≥ 1, are defined analogously.

The causal relationships in the system postulated above can be plotted using causal diagrams (see Figure 1). Causal diagrams are well-defined mathematical objects that consist of nodes and edges. Pearl’s approach focuses on causal diagrams without feedback or causal loops, that is, without directed paths that traverse the same variable more than once. These causal diagrams are termed *directed acyclic graphs* (DAG) and represent the researcher’s theory and prior (empirical) knowledge about the causal relationships between the variables.^1^ A key advantage of representing the hypothesized causal process as a DAG is that it allows the researcher to analyze the causal system using mathematical tools from the fields of graph theory and graphical modeling (Cowell et al., 1999; Jordan, 1998; Koller & Friedman, 2009; Pearl, 2009). These mathematical tools allow researchers to establish whether specific causal effects can be theoretically estimated given observable data (causal identification). They also help researchers to recognize the key variables needed for covariate adjustment and to find the testable implications of the causal system. Some of these tools are implemented in the user-friendly program DAGitty (Textor et al., 2016). In linear additive models, the insights provided by these mathematical tools have substantial overlap with the insights available from the tracing rules (Loehlin & Beaujean, 2017; Wright, 1934) and covariance algebra (Bollen, 1989) traditionally used in structural equation modeling. Unlike the tracing rules, the mathematical tools from graph theory can be applied to nonlinear and nonparametric models.

We initially consider a dynamic system representing a single person from a population of *homogenous* individuals. In the section on “Between-Person Heterogeneity” we extend the approach to consider potential individual differences in the mean level (unobserved heterogeneity). To keep the illustration simple we further assume that the data generating mechanism is linear and can be represented by the following set of linear structural equations:^2^ *t* = 1, 2, …, *T* = 4

(1a) $$X_{t+1}=c_{x_{t+1}x_{t}}X_{t}+c_{x_{t+1}y_{t}}Y_{t}+\varepsilon_{x_{t+1}}$$

(1b) $$Y_{t+1}=c_{y_{t+1}x_{t}}X_{t}+c_{y_{t+1}y_{t}}Y_{t}+\varepsilon_{y_{t+1}}$$

Error terms (residuals, disturbances) are denoted by *ε* and a subscript indicating the corresponding variable. For example, $\varepsilon_{x_{t+1}}$ denotes the error term corresponding to variable *X*_*t*+1_. The error term $\varepsilon_{x_{t+1}}$ captures those factors that determine the value of *X*_*t*+1_ that are not explicitly included in the model (omitted variables).

Consider the DAG in Figure 1 that represents the causal relationships for our simple two variable, four-wave illustration. Each solid single-headed arrow represents a direct causal relationship in this model. For simplicity of presentation, we will assume time-stable structural coefficients throughout the article. That is, we will assume that the autoregressive coefficients $c_{x_{t+1}x_{t}}$, *t* ≥ 1, for the *X*-series and the autoregressive coefficients $c_{y_{t+1}y_{t}}$, *t* ≥ 1, for the *Y*-series are constant over time. Likewise, we will assume that the cross-lagged coefficients from *X* at time *t* to *Y* at time *t* + 1, denoted by $c_{y_{t+1}x_{t}}$, *t* ≥ 1, and the cross-lagged coefficients from *Y*_*t*_ to *X*_*t*+1_, denoted by $c_{x_{t+1}y_{t}}$, *t* ≥ 1, are constant over time. In addition, we will assume that the variances of the disturbances corresponding to the *X*-series, denoted by $\psi_{x_{t},x_{t}}$, *t* ≥ 2, and those corresponding to the *Y*-series, denoted by $\psi_{y_{t},y_{t}}$, *t* ≥ 2, are constant over time.

Traditionally, error terms are not explicitly drawn in a DAG and the following conventions are used: (a) A bidirected edge drawn between two variables in the DAG indicates that the corresponding *error terms* covary due to unobserved confounding. For example, we assume an unobserved common cause of *X*_1_ and *Y*_1_ that implies a covariance between the corresponding error terms $\varepsilon_{x_{1}}$ and $\varepsilon_{y_{1}}$, denoted as $\psi_{x_{1},y_{1}}=\text{COV}\left(\varepsilon_{x_{1}},\varepsilon_{y_{1}}\right)$ (for interested readers, a detailed discussion of the specification of the first measurement occasion is provided in the Appendix on Initial Variables). (b) The absence of a bidirected edge between two variables reflects the assumption that there is no unobserved common cause. For example, we assume that there is no unobserved confounder for the *X*_2_ → *Y*_3_ relationship. Throughout this article, variances and covariances of disturbances are denoted by *ψ* and a subscript. For example, $\psi_{x_{t},x_{t}}$ denotes the variance of the disturbance $\varepsilon_{x_{t}}$, that is, $\psi_{x_{t},x_{t}}=\text{V}\left(\varepsilon_{x_{t}}\right)$.

For our illustration, we simulate data from a known data generating process. Numerical values have been assigned to each parameter in the DAG based on the values from a panel data study by Ito et al. (1998). These numerical values are displayed in Tables 1 and 5 and will be used to illustrate numerical calculations of causal effects later in the paper (see online supplementary material for a detailed account of the data generation). Note that these numerical values yield a stable and covariance stationary system (Hamilton, 1994).^3^ Based on these values we obtain a standard deviation of blood insulin of SD(*X*_*t*_) = 11.48 mcIU/ml and a standard deviation of blood glucose of SD(*Y*_*t*_) = 25.16 mg/dl, *t* ≥ 1.

**Figure 2.**
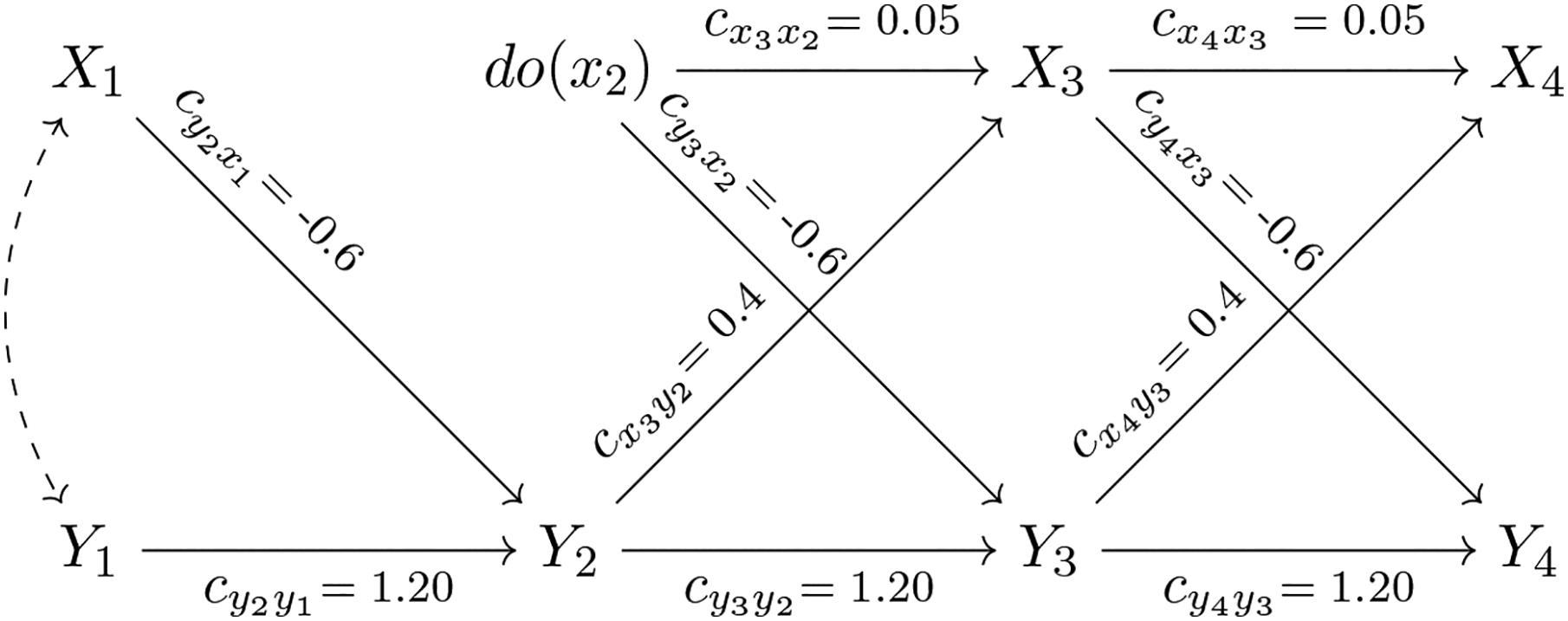
The causal diagram (DAG) displayed in Figure 1 is modified to account for the intervention *do*(*x*_2_). The variable *X*_2_ has been replaced by *do*(*x*_2_) and the solid single-headed arrows entering *X*_2_ have been removed, indicating that the interventional level *do*(*x*_2_) does not depend on *X*_1_ or *Y*_1_ but is set by an experimenter (in our illustrative example we choose *do*(*X*_2_ = 11.48)). Note that all other variables and directed edges (causal arrows) remain unchanged as compared to the situation without the intervention *do*(*x*_2_) as depicted in Figure 1 reflecting the assumption of modularity.

Figure 2 modifies Figure 1 by introducing a specific form of manipulation introduced by Pearl. Historically in psychology, we have thought of a manipulation as adding (subtracting) a constant to the person’s current level on *X*. Thus, if a dose of insulin were given, the person’s insulin level would be expected to experience a fixed increase to a level that reflects both the dose received and the person’s baseline insulin level prior to the manipulation. In contrast, Pearl (2009) proposed a manipulation that fixes a person’s level on *X* to a constant value *x*. The latter type of manipulation is formalized via the *do*-operator and has proven to be very useful for theoretically defining and quantifying causal effects. For example, the *do*-operator applied to variable *X*_*t*_ is denoted by *do*(*X*_*t*_ = *x*_*t*_) or equivalently abbreviated as *do*(*x*_*t*_). By definition, the *do*(*x*_*t*_)-operator has the following properties: It (a) fixes the level of *X*_*t*_ at *x*_*t*_ for each unit in the population, where *x*_*t*_ is referred to as the interventional level. The interventional level *x*_*t*_ is (b) not related to any causally prior variable. Finally, the *do*(*x*_*t*_)-operator (c) leaves all other relationships in the dynamic system unchanged.

Part (a) reflects the manipulation in a hypothetical experiment in which the level of the treatment variable *X*_*t*_ is set by an experimenter. By fixing the level of the variable, each unit will have the identical value of *X*_*t*_ following the *do*(*x*_*t*_)-manipulation and *X*_*t*_ will have 0 variance under the intervention. In other words, the *do*(*x*_*t*_)-operation reflects a hypothetical experiment in which the treatment *X*_*t*_ = *x*_*t*_ is applied uniformly over the whole population. Part (b) of the definition of the *do*(*x*_*t*_)-operator reproduces the feature of randomized experiments that all prior covariates are independent of the treatment variable. Part (c) of the definition of the *do*(*x*_*t*_)-operator, an assumption formally known as autonomy or modularity (Pearl, 2009; Peters et al., 2017; Spirtes et al., 2000), implies that the intervention *do*(*x*_*t*_) only alters the value of *X*_*t*_ and does not change the relationships between other measured variables not involved in the manipulation.

Figure 2 illustrates the *do*-operator applied to insulin levels at time *t* = 2, denoted as *do*(*x*_2_). Here, we imagine that each person’s insulin level is set to the interventional value *X*_2_ = 11.48 mcIU/ml, that is, one standard deviation above the person’s mean level (which is 0 for each person after mean-centering in a homogeneous population). The causal links of *X*_2_ to its direct predecessors in the causal chain (here: *X*_1_ and *Y*_1_) have been removed, but all other causal relationships in the model are maintained as before the *do*(*x*_2_)-operation.

Up to this point, we have discussed the data generating mechanism but have not yet introduced the statistical model we will use for data analysis. Since we use simulated data the choice of a statistical model is straightforward: We chose a correctly specified statistical model, that is, one that exactly captures the data generating process described above. In our example, we will use a bivariate linear cross-lagged panel model with four measurement waves. This model can be equivalently understood as a linear structural equation model. Thus, it can be represented by a causal path diagram as commonly used to represent linear SEM (Bauer, 2003; Curran, 2003). These causal path diagrams are similar to DAGs, as long as we restrict our attention to linear models without feedback or causal loops. An important difference, however, is that a dashed double-headed arrow in a path diagram represents a covariance between variables (or disturbances) that is not further analyzed. In the context of DAG-based causal inference, a dashed double-headed arrow represents a covariance that stems from an unobserved confounder. Throughout this paper, we draw DAGs and therefore double-headed arrows always indicate the existence of an unobserved confounder. From a statistical point of view, the linear bivariate cross-lagged panel model that captures the DAG depicted in Figure 1 is globally identified, that is, all parameters can theoretically be estimated uniquely from observational data (Bollen & Curran, 2004; Hsiao, 2014).

Throughout this paper, we know with certainty that the causal assumptions encoded in the DAG in Figure 1 are correct since we use simulated data. Note that all our data stems from the DAG depicted in Figure 1, that is, we have not actually performed an experiment. This *non-experimental* evidence is combined with our assumptions about the data generating mechanism to forecast the effect of the (hypothetical) intervention (see Figure 2). In practical applications, however, one usually does not know the true data generating mechanism and causal assumptions postulated by researchers might be wrong. We discuss statistical tests of model assumptions and methods to analyze the sensitivity of causal conclusions with respect to violations of assumptions in the section on “Current Limitations and Future Directions.”

Given the important distinction between the system following the *do*(*x*_2_)-operation depicted in Figure 2 and the passive observational system in the absence of any intervention depicted in Figure 1, we now explore the implications of these different situations for (a) forecasting the treatment effect of *X*_2_ on *Y*_3_ and (b) predicting the value of *Y*_3_ based on *X*_2_ in the absence of interventions.

## Distinguishing between forecasting effects of interventions and predicting future outcomes

**Figure 3.**
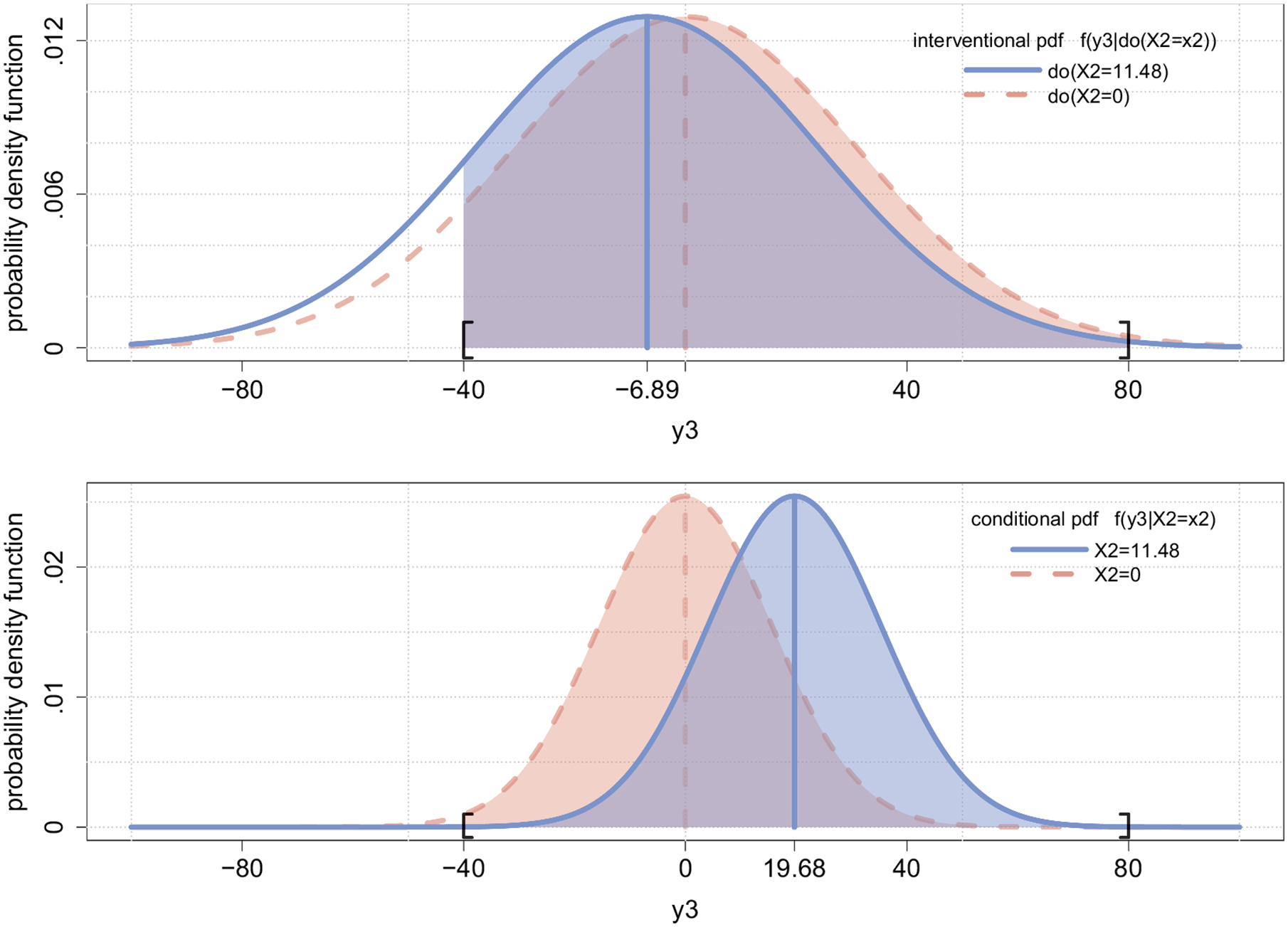
The upper panel displays the probability density functions (pdf) of the interventional distribution *P*(*Y*_3_|*do*(*x*_2_)) for two distinct treatment levels, namely *do*(*X*_2_ = 11.48) (solid line) and *do*(*X*_2_ = 0) (dashed line). Means are depicted as solid and dashed vertical line segments. The lower panel displays the pdf of the conditional distribution *P*(*Y*_3_|*X*_2_ = *x*_2_) for two distinct observed levels of blood insulin at time *t* = 2, namely *X*_2_ = 11.48 (solid line) and *X*_2_ = 0 (dashed line). The marked interval on the horizontal axes ([−40, 80], person mean-centered metric) represents the range of acceptable mean-centered blood glucose levels. The shaded areas under the curves (designated by square brackets) represent the probability that blood glucose level at time *t* = 3 falls in the acceptable range.

The effect of a treatment is always defined relative to another active or control treatment (Holland, 1986). The *do*-operator allows us to represent a specific type of treatment effect by comparing the distribution of an outcome variable under the intervention *do*(*x*_*t*_) (representing the treatment) to that of a second intervention $\text{do}\left(x_{t}^{\prime }\right)$ (representing the control). The upper panel of Figure 3 displays the distribution of *Y*_3_ given the interventions *do*(*X*_2_ = 11.48) (treatment; solid line) and *do*(*X*_2_ = 0) (control; dotted line).

We define the distribution of the outcome value *Y*_*t*+*k*_ in the population given the treatment *do*(*x*_*t*_) as the interventional distribution, denoted by P(*Y*_*t*+*k*_|*do*(*x*_*t*_)), where *k* is the number of waves after the intervention when the outcome is measured.^4^ In this paper, we restrict our attention to the interventional distribution *k* = 1 wave after the treatment has been applied. To compare the outcome variable *Y*_*t*+1_ given two distinct interventions *do*(*x*_*t*_) and $\text{do}\left(x_{t}^{\prime }\right)$, one has to compare the two random variables *Y*_*t*+1_|*do*(*x*_*t*_) and $Y_{t+1}|\text{do}\left(x_{t}^{\prime }\right)$. This comparison can, for example, be done visually by plotting the probability density functions (pdf) of the two interventional distributions P(*Y*_*t*+1_|*do*(*x*_*t*_)) and $\text{P}\left(Y_{t+1}|\text{do}\left(x_{t}^{\prime }\right)\right)$, as displayed in the upper panel of Figure 3. In our illustration, we compare the values of blood glucose at time *t* = 3 given two distinct interventions on blood insulin at *t* = 2, namely *do*(*X*_2_ = 11.48) and *do*(*X*_2_ = 0). The latter intervention sets the value of blood insulin to the mean value (= 0 in mean-centered metric), the former intervention to one standard deviation above the mean. The upper panel of Figure 3 depicts the probability density functions of the corresponding interventional distributions *P*(*Y*_3_|*do*(*X*_2_ = 0) (dashed line) and *P*(*Y*_3_|*do*(*X*_2_ = 11.48) (solid line).

Another common way to compare two distributions is to examine differences in their means. The difference between the interventional means $\text{E}\left(Y_{3}|\text{do}\left(x_{t}\right)\right)-\text{E}\left(Y_{3}|\text{do}\left(x_{t}^{\prime }\right)\right)$ is called the average treatment effect (ATE) of treatment *do*(*x*_*t*_) relative to treatment $\text{do}\left(x_{t}^{\prime }\right)$ on *Y*_*t*+1_, abbreviated as the ATE of *X*_*t*_ on *Y*_*t*+1_ (Pearl, 2009). In our illustration, we analyze the ATE of treatment *do*(*X*_2_ = 11.48) relative to treatment *do*(*X*_2_ = 0) on *Y*_*t*+1_, denoted as E(*Y*_3_|*do*(*X*_2_ = 11.48)) E(*Y*_3_|*do*(*X*_2_ = 0)). The interventional means are depicted in the upper panel of Figure 3 as the solid and the dashed vertical line segments.

The comparison of two distributions is not restricted to a comparison of their location (means). Another important feature of the interventional distribution is its variance. The variances of the outcome distributions under the distinct treatments *do*(*x*_*t*_) and $\text{do}\left(x_{t}^{\prime }\right)$ correspond to the *widths* of the bell-shaped curves in the upper panel of Figure 3. The two curves in the upper panel have the same widths, that is, the error when forecasting the effect of an intervention on *X*_2_ on the level of *Y*_3_ is the same for *do*(*X*_2_ = 11.48) and *do*(*X*_2_ = 0).

Comparisons that go beyond first- and second-order moments (means and variances) may be of importance in a number of research situations involving dynamic systems. For example, Monnier et al. (2017) propose the frequency of hypoglycemia (adversely low levels of blood glucose) as a target quantity that should be minimized during treatment. In the section titled “Interventional Probabilities versus Conditional Probabilities” we will consider the probability that a *do*-operation will lead to glucose levels that fall in an acceptable range of values that is specified a priori.

Throughout this article, we will make a clear distinction between (a) the forecast of the outcome value *Y*_*t*+1_ given an intervention *do*(*x*_*t*_), and (b) the prediction of the value of *Y*_*t*+1_ given the passive observation that variable *X* takes the value *x* at time *t*, denoted by *X*_*t*_ = *x*_*t*_. Returning to our example, in case (b) we would like to predict the value of blood glucose at *t* = 3 based on the passive observation (e.g., self-measured monitoring of blood insulin) that the insulin level is one standard deviation above the mean level at *t* = 2. We will, therefore, use the conditional distribution of *Y*_3_, given *X*_2_ = 11.48, denoted by P(*Y*_3_|*X*_2_ = 11.48), to predict the value of blood glucose at *t* = 3. We use the term *predictions* (of future values) to refer to probabilistic statements that are based on the conditional distribution.

Comparing the two panels of Figure 3 provides a visual representation of the differences between (a) forecasts of the effects of interventions and (b) predictions of future values. Observing *X*_2_ = 11.48 yields the conditional distribution P(*Y*_3_|*X*_2_ = 11.48) represented by the solid line in the lower panel, whereas administering the treatment *do*(*X*_2_ = 11.48) yields the interventional distribution P(*Y*_3_|*do*(*X*_2_ = 11.48)) represented by the solid line in the upper panel. The conditional distribution of the predicted values of blood glucose levels is shifted to the right relative to the interventional distribution of the *do*(*x*_2_)-forecast of blood glucose levels.

Given this initial impression of the differences between (a) the interventional and (b) the conditional distribution, we now systematically analyze the differences in mean levels and variances, followed by the probabilities of blood glucose levels falling in an acceptable range. As part of this systematic comparison, we present formulae for interventional distributions and the moments thereof (see also Gische & Voelkle, 2020). Formulae for conditional distributions and moments thereof are established results for the multivariate normal distribution (e.g., Rao, 1973).

For ease of presentation we will drop the time indices of the structural coefficients in the equations. For example, we will write *c*_*xx*_ instead of $c_{x_{2}x_{1}}$, $c_{x_{3}x_{2}}$, and $c_{x_{4}x_{3}}$ for the autoregressive coefficients of the *X*-series, because these coefficients are assumed to be stable over time. Analogously, we will write *c*_*yy*_ for the autoregressive coefficients of the *Y*-series as well as *c*_*xy*_ and *c*_*yx*_ for the cross-lagged coefficients. Likewise, we will drop the time indices of the variance parameters for *t* ≥ 2. We will write *ψ*_*xx*_ instead of $\psi_{x_{2}x_{2}}$, $\psi_{x_{3}x_{3}}$, and $\psi_{x_{4}x_{4}}$ and *ψ*_*yy*_ instead of $\psi_{y_{2}y_{2}}$, $\psi_{y_{3}y_{3}}$, and $\psi_{y_{4}y_{4}}$. All numeric quantities are calculated based on the parameter values displayed in Tables 1 and 5.

A complete presentation of all relevant formulae and details regarding the derivation thereof is provided in the online supplementary material together with a justification of the numerical values based on prior empirical results reported by Ito et al. (1998).

### Interventional mean versus conditional mean

In this section we systematically compare the mean level of blood glucose at *t* = 3 given (a) a *do*(*x*_2_)-intervention has been applied on insulin levels at *t* = 2 and (b) prior observations on the levels of *X*_2_ are available.

We start with a comparison of the baseline scenarios in which (a) no *do*-treatment is applied (denoted by *do*(∅)), and (b) no prior observations are available (denoted by the empty set ∅). In both situations, the best guess of the value of *Y*_3_ is zero since the data are mean-centered within each person (see row 1 of Table 2). Put more formally, in the baseline scenarios the interventional mean E(*Y*_3_|*do*(∅)) and the conditional mean E(*Y*_3_|∅) are both equal to the unconditional mean, that is, E(*Y*_3_|*do*(∅)) = E(*Y*_3_|∅) = E(*Y*_3_) = 0.

We now turn to a comparison of the scenarios where in case (a) the level of blood insulin has been set to *do*(*X*_2_ = 11.48) and in case (b) the value *X*_2_ = 11.48 has been observed (see row 2 of Table 2).

In case (a), the intervention *do*(*X*_2_ = 11.48) is performed, which sets the value of blood insulin to one SD above the mean level at time *t* = 2. Due to the intervention both paths entering into *X*_2_ are removed (see Figure 2). The formula for the interventional mean and its numeric evaluation are given by:

(2) $$\text{E}\left(Y_{3}|\text{do}\left(x_{2}\right)\right)=c_{yx}x_{2}\;\text{E}\left(Y_{3}|\text{do}\left(X_{2}=11.48\right)\right)=-0.6\cdot 11.48=-6.89$$

Thus, the mean forecast of *Y*_3_ given the intervention *do*(*X*_2_ = 11.48) is − 6.89 mg/dl (see also row 2, column 3 of Table 2). Recall that the ATE of a treatment is always defined relatively to another treatment. Here we analyze the ATE of treatment *do*(*X*_2_ = 11.48) relative to the treatment *do*(*X*_2_ = 0). Thus, the resulting ATE is equal to E(*Y*_3_|*do*(*X*_2_ = 11.48)) − E(*Y*_3_|*do*(*X*_2_ = 0)) = −6.89 − 0 = −6.89. In other words, actively changing the level of blood insulin at *t* = 2 from 0 to 11.48 (= one SD above the mean) results in a difference of −6.89 mg/dL in the forecasted blood glucose level at time *t* = 3.

In case (b) observing a blood insulin level of one SD above the person’s mean at time *t* = 2 leads to a predicted value of 1.71 · 11.48 = 19.63 mg/dl of blood glucose at time *t* = 3 (see row 2, column 6 of Table 2). The coefficient 1.71 is the population regression coefficient in a simple linear regression of *Y*_3_ on *X*_2_ and is calculated according to the following formula:

(3) $$\text{E}\left(Y_{3}|X_{2}=x_{2}\right)=\frac{\text{COV}\left(X_{2},Y_{3}\right)}{\text{V}\left(X_{2}\right)}x_{2}\;=\left(c_{yx}+\frac{c_{xx}c_{yx}c_{yy}\psi_{x_{1}x_{1}}+c_{xy}c_{yy}^{2}\psi_{y_{1}y_{1}}+\left(c_{xy}c_{yx}c_{yy}+c_{xx}c_{yy}^{2}\right)\psi_{x_{1}y_{1}}}{c_{xx}^{2}\psi_{x_{1}x_{1}}+c_{xy}^{2}\psi_{y_{1}y_{1}}+2c_{xx}c_{xy}\psi_{x_{1}y_{1}}+\psi_{xx}}\right)x_{2}\;=\left(-0.6+2.31\right)x_{2}=1.71x_{2}$$

The last line contains an numeric evaluation based on the parameter values displayed in Table 1. The population regression coefficient represents the sum of the direct causal effect and the spurious effects of *X*_2_ on *Y*_3_. These spurious effects correspond to backdoor paths from *X*_2_ to *Y*_3_ in Pearl’s terminology. One such backdoor path is *X*_2_ ← *X*_1_ → *Y*_2_ → *Y*_3_; another backdoor path is from *X*_2_ ← *Y*_1_ → *Y*_2_ → *Y*_3_ (see Figure 1). Observing a mean blood insulin level (=0) at time *t* = 2 leads to a predicted value of 1.71 · 0 = 0 mg/dl of blood glucose at time *t* = 3. Thus, based on traditional methods for prediction (e.g., linear regression), we would obtain a difference of 19.63 mg/dL between the predicted values of *Y*_3_ given we observe a change in *X*_2_ from 0 to 11.48.

In summary, in case (a) changing the *do*(*x*_2_) treatment level by plus one SD yields a −6.89 mg/dL decrease in the forecasted value of blood glucose at time *t* = 3. In case (b) an observed one SD increase in blood insulin at wave 2 is predicted to produce a 19.63 mg/dL increase in blood glucose at time *t* = 3. Both findings provide useful information that need to be used for distinct purposes. In case (a), a physician would correctly forecast a negative value (−0.6 · 11.48 = −6.89 mg/dl) of blood glucose at time *t* = 3 after administering the dose *do*(*X*_2_ = 11.48) of insulin at time *t* = 2. In case (b), a patient obtaining the insulin reading *X*_2_ = 11.48 at time *t* = 2 would correctly predict a positive level (1.71 · 11.48 = 19.63 mg/dl) of blood glucose at time *t* = 3.

Incorrect conclusions only arise if the algebraic machinery of conditional expectations (case (b)) is used to forecast the effects of interventions (case (a)) – or the other way around – the interventional mean based on the *do*-operator (case (a)) is used to predict future values of *Y* in the absence of interventions (case (b)).

We have seen that interventional means (forecasts) and conditional expectations (predictions) are different quantities. The latter is a purely statistical quantity that can always be estimated based on observational data in a correctly specified model. The former is a causal quantity that might *not* be identified based on observational data even if the model is correctly specified. Pearl (2009) provides DAG-based criteria for the identification of specific causal effects, some of which are implemented in the computer program DAGitty (Textor et al., 2016). For example, to identify the ATE of *X*_2_ on *Y*_3_ in the present example, a graphical rule called the backdoor criterion can be used. The backdoor criterion states that observing a set of variables which blocks all backdoor paths from *X*_2_ to *Y*_3_ provides sufficient information to correctly calculate the ATE based on non-experimental data. In the present example, the minimal sufficient backdoor adjustment set for the ATE of *X*_2_ on *Y*_3_ is {*Y*_2_}.^5^ Thus, if *Y*_2_ is held constant or statistically adjusted for, all backdoor paths are blocked.

Backdoor adjustment in linear models can for example, be obtained by linear regression. Thus, the ATE *X*_2_ on *Y*_3_ can be estimated using a linear regression of *Y*_3_ on *X*_2_ *and Y*_2_ (Pearl, 2009, Theorem 5.3.2). The resulting coefficient of *X*_2_ in the proposed regression of *Y*_3_ on *X*_2_ *and Y*_2_ is equal to − 0.6 (see row 3, column 6 of Table 2) which equals the coefficient in the interventional mean expression (see row 2, column 3 of Table 2). Thus, if the backdoor criterion is satisfied in a linear model, the interventional mean (causal quantity) can be estimated from observational data using linear regression.

### Interventional variance versus conditional variance

This section studies the interventional variance of the outcome variable following a *do*-operation, a feature of the interventional distribution that has received less attention than the interventional mean. Using our running example, we focus on the variance of blood glucose at *t* = 3.

In the baseline scenarios in which (a) no *do*-treatment is applied (denoted by *do*(∅)), and (b) no prior observations are available (denoted by the empty set ∅), the interventional variance V(*Y*_3_|*do*(∅)) and the conditional variance V(*Y*_3_|∅) are both equal to the unconditional variance V(*Y*_3_). All quantities take on the value 632.93, as displayed in row 1 of Table 3.

In the left part of Table 3 (columns below (a) intervention) the interventional variances are displayed. For example, the interventional variance V(*Y*_3_|*do*(*x*_2_)) can be calculated according to the following formula (see online supplementary material for derivation):

(4) $$\text{V}\left(Y_{3}|\text{do}\left(x_{2}\right)\right)=c_{yx}^{2}c_{yy}^{2}\psi_{x_{1}x_{1}}+c_{yy}^{4}\psi_{y_{1}y_{1}}+2c_{yx}c_{yy}^{3}\psi_{x_{1}y_{1}}\;+\left(1+c_{yy}^{2}\right)\psi_{yy}$$

Note that the interventional level *x*_2_ does *not* appear on the right-hand side of Equation (4). Thus, the interventional variance (in linear models with normally distributed error terms) does not depend on the exact level *x*_2_ of the *do*-treatment that is applied. This can also be seen in the upper panel of Figure 3: The *shape* of the interventional distributions is the same for both treatment levels *do*(*X*_2_ = 0) (dashed line) and *do*(*X*_2_ = 11.48) (solid line). If a patient is treated at time *t* = 2 and we change the treatment dose (e.g., set the insulin level to 11.48 instead of 0), only the location of the interventional distribution changes and the forecast variance (*width* of the bell-shaped curves) is not affected by the specific level to which blood insulin is set by the experimenter.

In contrast, the interventional variance does change as we introduce additional interventions on different variables in the model. This can be verified by comparing the value in row 1 with the value of row 2 in column 3 of Table 3. These values illustrate the case where we administer the treatment *do*(*x*_2_) to a formerly untreated (*do*(∅)) person: the forecast variance *increases* from 632.93 to 951.43. An increase in variance might appear counter-intuitive at first glance. However, our illustration considers the dynamic interplay of blood insulin and blood glucose in a population of *healthy* (non-diabetic) persons. For such a population the glucose and insulin levels are governed by a self-regulating dynamic system. In our example the external intervention temporarily eliminates the ability of the system to self-regulate. Consequently, the system becomes temporarily destabilized and the variance increases.

In case (b), the conditional variances used for prediction decrease (632.93 > 245.71 > 40.00) as we successively move from row 1 to row 3 in column 6 of Table 3. This result is intuitive: As we move from one row to the row below in the right part of Table 3, more observational information becomes available. Increasing the amount of available observational information (that stems from the same data generating mechanism) increases the precision of prediction as reflected in the sequence of decreasing conditional variances. The conditional variance V(*Y*_3_|*X*_2_ = *x*_2_) is calculated according to the following general formula (Rao, 1973):

(5) $$\text{V}\left(Y_{3}|X_{2}=x_{2}\right)=\text{V}\left(Y_{3}\right)-\frac{\text{COV}{\left(X_{2},Y_{3}\right)}^{2}}{\;\text{V}\left(X_{2}\right)}$$

Note that *x*_2_ does *not* appear on the right-hand side of Equation (5). In other words, unlike the conditional expectations in Table 2, the conditional variance (in linear models with normally distributed error terms) does not depend on the exact value *x*_2_ of the variable on which we condition. This result is also depicted in panel (b) of Figure 3, where the *shape* of the conditional distributions is the same for both observations *X*_2_ = 0 (dashed line) and *X*_2_ = 11.48 (solid line). The two curves only differ in location but have the same *width*.

So far, we have seen that the interventional variance and the conditional variance are different quantities. The conditional variance is a purely statistical quantity used to represent prediction error, whereas the interventional variance is a causal quantity used to represent forecast error when assessing the effect of a *do*-type intervention. The interventional variance (causal quantity) is identified since the backdoor criterion introduced in the section titled “Interventional Mean versus Conditional Mean” is not restricted to interventional means but applies to the entire interventional distribution (Pearl, 2009).

Given identification is ensured, the question arises whether there is a simple way to estimate the interventional variance of *Y*_3_ from observational data. In our example, the question is: Can we compute the numerical value V(*Y*_3_|*do*(*x*_2_)) = 951.43 (*causal* quantity) from the numerical values of the *statistical* quantities in column 6 of Table 3? The answer is yes, but the procedure is complex and also requires information from column 6 of Table 2 (Kuroki & Cai, 2007). Unfortunately, the standard backdoor adjustment procedure for linear models (i.e., linear regression) that is used to estimate the interventional mean cannot be used to estimate interventional variances.

This problem can be resolved by using the technique for causal identification and estimation in linearly parameterized causal DAG-models suggested by Gische and Voelkle (2020). The central idea is that a causal quantity defined via the *do*-operator can be expressed as a function of the parameters of the statistical model. This idea can be illustrated for the interventional variance as stated in Equation (4). The expression on the left-hand side of Equation (4) is V(*Y*_3_|*do*(*x*_2_)) which is a causal quantity and contains the *do*-operator. The expression on the right-hand side is a polynomial function of the model parameters *c*_*yx*_, *c*_*yy*_, $\psi_{x_{1}x_{1}}$, $\psi_{y_{1}y_{1}}$, $\psi_{x_{1}y_{1}}$, *ψ*_*yy*_ and does *not* contain the *do*-operator. In our working example all parameters that appear on the right-hand-side of Equation (4) (i.e., *c*_*yx*_, *c*_*yy*_, $\psi_{x_{1}x_{1}}$, $\psi_{y_{1}y_{1}}$, $\psi_{x_{1}y_{1}}$, *ψ*_*yy*_) are identified in the statistical model and can be estimated from observational data. Therefore, the interventional variance V(*Y*_3_|*do*(*x*_2_)) on the left-hand side of Equation (4) is also identified and estimates can be obtained from observational data.

### Interventional probabilities versus conditional probabilities

For some phenomena, it is important that a variable in a dynamic system falls within a predefined acceptable range. In our running example, the physician’s goal is that the patient’s level of blood glucose will be maintained in an acceptable range (so-called euglycemic range) after a treatment. A patient’s glucose level should not exceed a critical level (*y*^upper^, upper threshold) where she will experience hyperglycemia. Nor should a patient’s glucose level fall below a critical level (*y*^lower^, lower threshold) where she will experience hypoglycemia (Bode et al., 2005). The thresholds *y*^upper^ and *y*^lower^ are defined a priori based on medical standards. Each condition outside the acceptable range poses a threat to a patient’s health and should, therefore, be avoided.

We use the values *y*^upper^ = 80 mg/dl and *y*^lower^ = −40 mg/dl in our example. The original upper (180 mg/dl) and lower (60 mg/dl) thresholds were centered around a global mean of 100 mg/dl (Cryer, 2003; Danaei et al., 2011; Giugliano et al., 1997). Thus, a physician is interested in the event that *Y*_3_ falls in the acceptable range [−40, 80] in the mean-centered metric, given the treatment. We refer to the latter event as *successful treatment* and introduce an indicator variable $Y_{3}^{*}$ that is equal to 1 in case of a successful treatment and 0 otherwise. The probability of a successful treatment is denoted by P(−40 < *Y*_3_ < 80|*do*(*x*_2_)) or in brief $\text{P}\left(Y_{3}^{*}=1|\text{do}\left(x_{2}\right)\right)$. The shaded area under the solid curve in the upper panel of Figure 3 represents the probability of a successful treatment given the intervention *do*(*x*_2_ = 11.48).

We now turn to a systematic analysis of interventional probabilities and contrast them with conditional probabilities. In the baseline scenarios in which (a) no *do*-treatment is applied (denoted by *do*(∅)), and (b) no prior observations are available (denoted by the empty set ∅), the probability that blood glucose levels fall in the acceptable range at *t* = 3 is .94 as displayed in the first row of Table 4.

**Figure 4.**
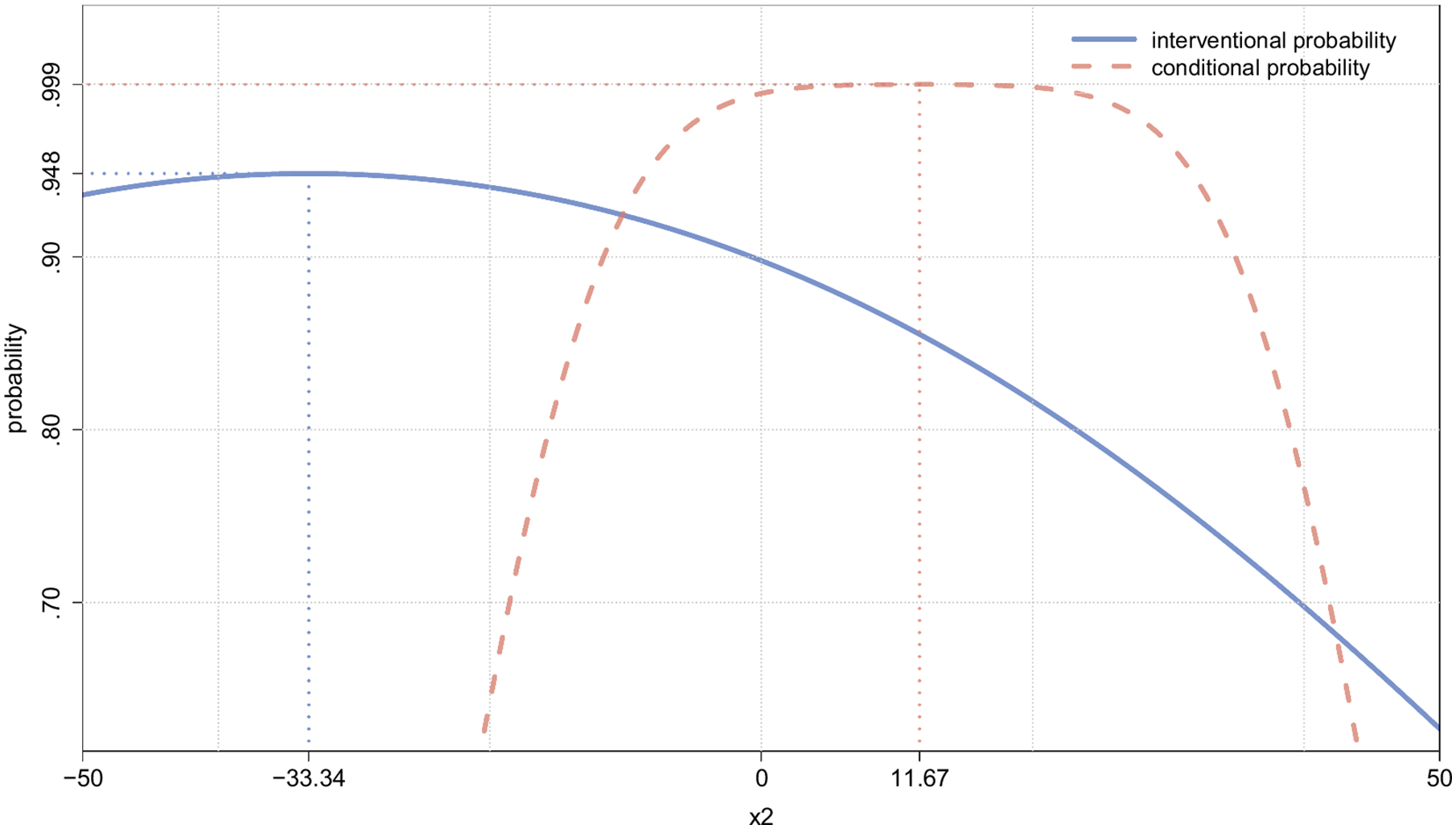
The interventional probability $\text{P}\left(Y_{3}^{*}=1|\text{do}\left(x_{2}\right)\right)$ on the vertical axis is depicted as a function of the (mean-centered) interventional level on the horizontal axis (solid line). In addition, the conditional probability $\text{P}\left(Y_{3}^{*}=1|X_{2}=x_{2}\right)$ on the vertical axis is depicted as a function of the (mean-centered) observed level *X*_2_ = *x*_2_ on the horizontal axis (dashed line). $Y_{3}^{*}=1$ whenever blood glucose level at time *t* = 3 falls within the acceptable range. A vertical dotted line is drawn at the interventional level *do*(*X*_2_ = −33.34) that maximizes the probability of treatment success. Another vertical dotted line is drawn at the observed measurement level *X*_2_ = 11.67 that maximizes the prediction probability that blood glucose levels will be within the acceptable range at time *t* = 3 (in the absence of an intervention).

In case (a), administering *do*(*X*_2_ = 11.48) at time *t* = 2 yields a probability of blood glucose values within the acceptable range at time *t* = 3 equal to .86 (see row 2 column 3 in Table 4). The interventional probability of treatment success depends on the level of blood insulin that has been administered at *t* = 2 in a nonlinear way that exhibits a unique maximum as depicted by the solid line in Figure 4.^6^

In case (b), where a value of blood insulin equal to one standard deviation above the person’s mean level is observed at time *t* = 2, the probability of blood glucose values being within the acceptable range at time *t* = 3 is equal to .9999 (see row 2 column 6 of Table 4). Again, the conditional probability $\text{P}\left(Y_{3}^{*}=1|X_{2}=x_{2}\right)$ has a nonlinear relationship to the level of blood insulin observed at *t* = 2 which is depicted as the dashed line in Figure 4.

Both curves displayed in Figure 4 have a single unique maximum. In case (a) a physician who must choose an interventional level should apply the treatment *do*(*X*_2_ = −33.34) to maximize the probability of treatment success (solid vertical line). Recall that we use mean-centered variables with SD(*X*_2_) = 11.48. Thus, the treatment *do*(*X*_2_ = −33.34) corresponds to setting a patient’s insulin level to 2.9 standard deviations below the mean level. In case (b) a patient who is passively measuring blood insulin levels at *t* = 2 would hope to measure a value of *x*_2_ = 11.67, since observing this value maximizes the conditional probability of blood glucose levels within the acceptable range at *t* = 3 (dashed vertical line).

If a physician were to erroneously use the conditional distribution to optimize an intervention and applied the treatment *do*(*X*_2_ = 11.67), this non-optimal intervention would result in a 85.5% probability of treatment success. A physician who correctly used the interventional distribution to choose the optimal treatment would apply *do*(*X*_2_ = −33.34), resulting in a 94.8% probability of treatment success. Thus, the consequence of an incorrect decision yields a 9.3% percentage drop in the probability of treatment success.

In summary, we have seen that conditional probabilities (statistical quantity) and the interventional probabilities (causal quantity) differ. The statistical quantity can always be estimated from observational data in a correctly specified model, whereas the causal quantity needs to be identified. The backdoor criterion ensures causal identification of interventional probabilities in our example model. The exact formula of the interventional probability $\text{P}\left(Y_{3}^{*}=1|\text{do}\left(x_{2}\right)\right)$ is complex and the regression-based backdoor adjustment formula for linear models can not be applied to calculate interventional probabilities. Instead, techniques for causal identification and estimation in linearly parameterized causal DAG-models suggested by Gische and Voelkle (2020) can be applied as explicated in more detail at the end of the section titled “Interventional Variance versus Conditional Variance.”

## Between-person heterogeneity

At the beginning of this article, we assumed that individuals come from a homogeneous population. This assumption will often be unrealistic in practice. A treatment that would be optimal (e.g., the administered dose of insulin maximizes the probability that blood glucose realizes within the acceptable range) for an “average” person is not necessarily optimal for a given patient if the population from which the average was calculated consists of heterogeneous individuals (Holland, 1986; Molenaar, 2004; Sidman, 1960; Voelkle et al., 2018). In this section we incorporate person-specific differences in mean levels into our model and focus on person-specific effects of interventions, that is, we forecast the effect of an intervention for a single individual drawn from a heterogeneous population.

### Modeling between-person heterogeneity via random intercepts

We use a panel data design with a fixed number of time points, *T* = 4, and sample size *N* as presented in the Introduction. Authors from different disciplines have proposed a variety of approaches for including person-specific differences into this framework (e.g., Hsiao, 2014; Usami et al., 2019; Wooldridge, 2010; Zyphur et al., 2019).

**Figure 5.**
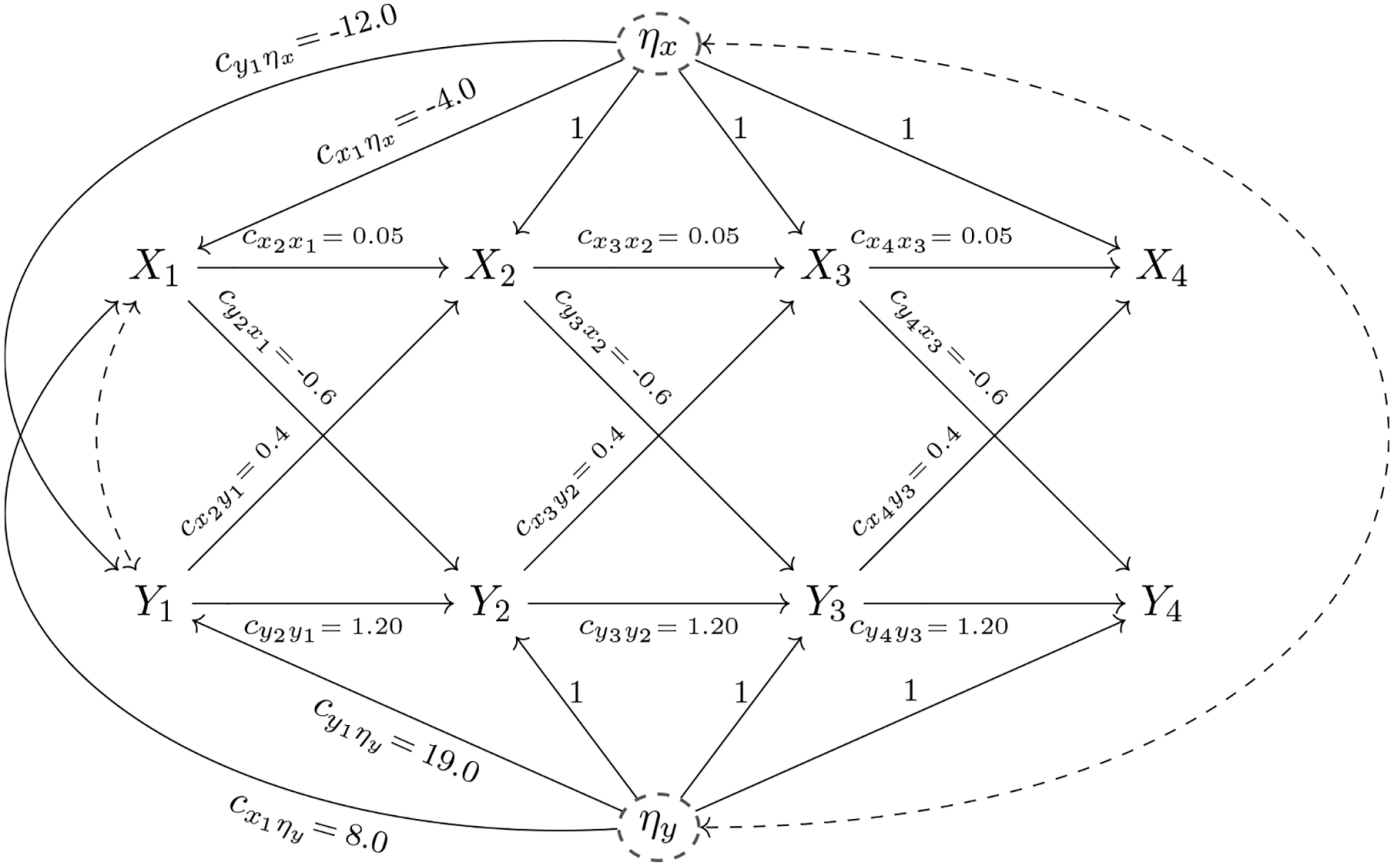
The causal diagram (DAG) displayed in Figure 1 is extended to include random intercepts (i.e., differences in the individual mean levels) that are represented in dashed circles, indicating that these variables are latent and therefore not observed. The directed edges from *η*_*x*_ to *X*_1_, …, *X*_4_ and *Y*_1_ indicate that time-invariant factors contained in the latent variables *η*_*x*_ have direct causal effects on blood insulin levels and the initial values of blood glucose levels. The directed edges from *η*_*y*_ to *Y*_1_, …, *Y*_4_ and *X*_1_ indicate that time-invariant factors contained in the latent variables *η*_*y*_ have direct causal effects on blood glucose levels and the initial values of blood insulin levels. The effects of the random intercepts on the initial variables are labeled with $c_{x_{1}\eta_{x}}$, $c_{x_{1}\eta_{y}}$, $c_{y_{1}\eta_{x}}$ and $c_{y_{1}\eta_{y}}$. The direct effects of *η*_*x*_ on *X*_2_, *X*_2_, and *X*_4_ and of *η*_*y*_ on *Y*_2_, *Y*_2_, and *Y*_4_ are assumed to be time-stable and equal to 1. The bidirected dashed edge between the random intercepts *η*_*x*_ and *η*_*y*_ indicate the existence of an unobserved confounder.

Here, we adopt the approach of including the latent variables *η*_*x*_ and *η*_*y*_ as additive random intercepts into the model (see Figure 5). The random intercept *η*_*x*_ is a term that includes all additive time-invariant factors that affect the level of blood insulin of a person; *η*_*y*_ is a term that includes all additive time-invariant factors that affect the level of blood glucose of a person. Thus, random intercepts capture those factors that vary across persons but do not change over time. For example, the random intercept could capture such factors as sex, marital status, and genetic endowment if these factors are constant during the study. Without loss of generality, we assume that random intercepts have zero means, that is, E(*η*_*x*_) = E(*η*_*y*_) = 0.

Note that these time-invariant variables (e.g., sex, marital status, and genetic endowment) are neither explicitly modeled nor measured in the study. Instead, the time-invariant variables are captured in the random intercepts (i.e., they are statistically accounted for).^7^ This approach gives random intercepts the status of latent variables as indicated by the dashed circles around *η*_*x*_ and *η*_*y*_ in the DAG representation of the model displayed in Figure 5. Since the random intercepts are latent we speak of *unobserved* heterogeneity.

The time-invariant variables collected in the random intercept *η*_*x*_ are assumed to have direct causal effects on the levels of blood insulin as indicated by the directed edges from *η*_*x*_ to *X*_1_, *X*_2_, *X*_3_, and *X*_4_ in Figure 5. For analogous reasons, directed edges from *η*_*y*_ to *Y*_1_, *Y*_2_, *Y*_3_, and *Y*_4_ are included in the DAG. The values of the structural coefficients corresponding to the paths from *η*_*x*_ to *X*_*t*_, *t* ≥ 2 are restricted to be equal to 1. These restrictions (a) assign a scale to the latent random intercept *η*_*x*_ and (b) reflect the assumption that the structural coefficients from the random intercepts to blood insulin levels *X*_*t*_, *t* ≥ 2 do not change over time. The same reasoning applies to the paths from *η*_*y*_ to *Y*_*t*_, *t* ≥ 2. The bidirected dashed edge between the random intercepts *η*_*x*_ and *η*_*y*_ indicates that time-invariant variables that determine blood insulin levels might covary with time-invariant variables that determine the level of blood glucose due to unobserved confounding. The random intercepts are assumed to be uncorrelated with the error terms $\varepsilon_{x_{t}}$, $\varepsilon_{y_{t}}$, *t* ≥ 1.

**Figure A1.**
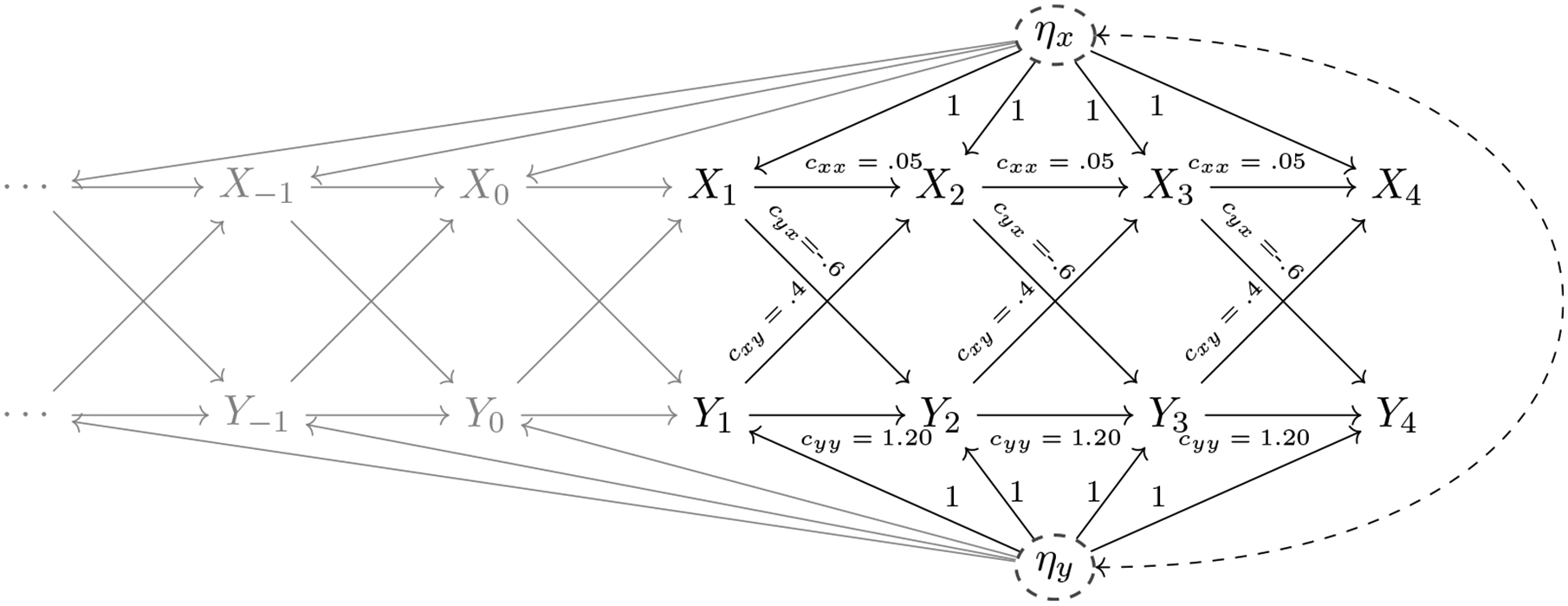
The depicted causal diagram (DAG) extends Figure 5 to account for the fact that the insulin-glucose dynamics were going on in the individuals prior to the first measurement wave. The four measurement waves that are observed in our study are drawn in black whereas the unobserved past of the process is drawn in gray. The graph in Figure A1 encodes the assumption, that the causal structure of the insulin-glucose dynamics is time-stable. In other words, the dynamics prior to the first measurement contain only autoregressive and cross-lagged effects of order one as well as direct effects from *η*_*x*_ to the insulin measures and from *η*_*y*_ to the glucose measures. We do not necessarily assume that the values of the structural coefficients are time-stable prior to the first measurement wave as indicated by leaving the directed edges in the gray part of Figure A1 unlabeled.

Special attention needs to be paid to the initial variables *X*_1_ and *Y*_1_ since they differ from the subsequent variables *X*_2_, *X*_3_, and *X*_4_ and *Y*_2_, *Y*_3_, and *Y*_4_ in an important way. The levels of *X*_*t*_ and *Y*_*t*_ are explained in our model by the values of *X*_*t*−1_ and *Y*_*t*−1_ when *t* ≥ 2. In contrast, the initial variables *X*_1_ and *Y*_1_ are exogenous in our model, that is, there are no incoming directed edges into *X*_1_ and *Y*_1_. The dynamics of the insulin–glucose relationship, however, were going on in the individuals prior to the first measurement wave. Thus, the initial variables somehow need to account for the past of the process that is not explicitly modeled (Figure A1 in the Appendix on Initial Variables).

Different approaches for incorporating initial variables into the statistical model have been proposed (Browne & Zhang, 2007; Hamaker et al., 2005; Hsiao, 2014; Molenaar, 1985). From a causal inference perspective, we believe the most straightforward and interpretable approach is to draw directed edges from $\eta_{x}\overset{c_{x_{1}\eta_{x}}}{\to }X_{1}$ and $\eta_{x}\overset{c_{y_{1}\eta_{x}}}{\to }Y_{1}$ as well as from $\eta_{y}\overset{c_{x_{1}\eta_{y}}}{\to }X_{1}$ and $\eta_{y}\overset{c_{y_{1}\eta_{y}}}{\to }Y_{1}$ (see Figure 5). A detailed justification of this modeling strategy is presented in the Appendix on Initial Variables where we also refer to viable alternatives. As noted in the previous paragraph, the initial variables differ in an important way from subsequent measurements. This special status is accounted for by not restricting the structural coefficients $c_{x_{1}\eta_{x}}$, $c_{y_{1}\eta_{x}}$, $c_{x_{1}\eta_{y}}$ and $c_{y_{1}\eta_{y}}$ to be equal to one.

A measure of the *degree* of unobserved heterogeneity in the population is the variance of the random intercepts. In the following, we assume the numerical values V(*η*_*x*_) = 5 and V(*η*_*y*_) = 10, where the time-invariant factors covary with COV(*η*_*x*_, *η*_*y*_) = 2.5. All parameters introduced to model person-specific differences in mean levels via random intercepts are collected in Table 5.

A consequence of modeling unobserved heterogeneity via random intercepts is an increase in total variance as compared to the model for the homogeneous population. For example, for *t* ≥ 2, the standard deviation of blood insulin levels increases from SD(*X*_*t*_) = 11.48 mcIU/ml in the homogeneous model to SD(*X*_*t*_) = 26.30 mcIU/ml in the model with random intercepts. Similarly, the standard deviation of blood glucose levels increases from SD(*Y*_*t*_) = 25.16 mg/dl to SD(*Y*_*t*_) = 61.83 mg/dl. A complete presentation of all relevant formulae and details regarding the derivation thereof is provided in the online supplementary material.

Figure 5 displays the causal diagram of the data generating mechanism. We use the bivariate linear cross-lagged panel model (*T* = 4) with random intercepts for data analysis. Since we use simulated data, we know with certainty that the causal model is correctly specified and that our statistical model captures the data generating process. In practical applications, however, one usually does not know the true data generating mechanism and causal assumptions postulated by researchers might be wrong. We discuss statistical tests of model assumptions and methods to analyze the sensitivity of causal conclusions with respect to violations of assumptions in the section on “Current Limitations and Future Directions.” From a statistical point of view, we believe that the model is identified (Hisao, 2014; Oud and Delsing, 2014, Zyphur et al., 2019), that is, all model parameters can be estimated from observational data and person-specific values of the random intercepts can be calculated.

### Average effects versus person-specific effects of interventions

**Figure 6.**
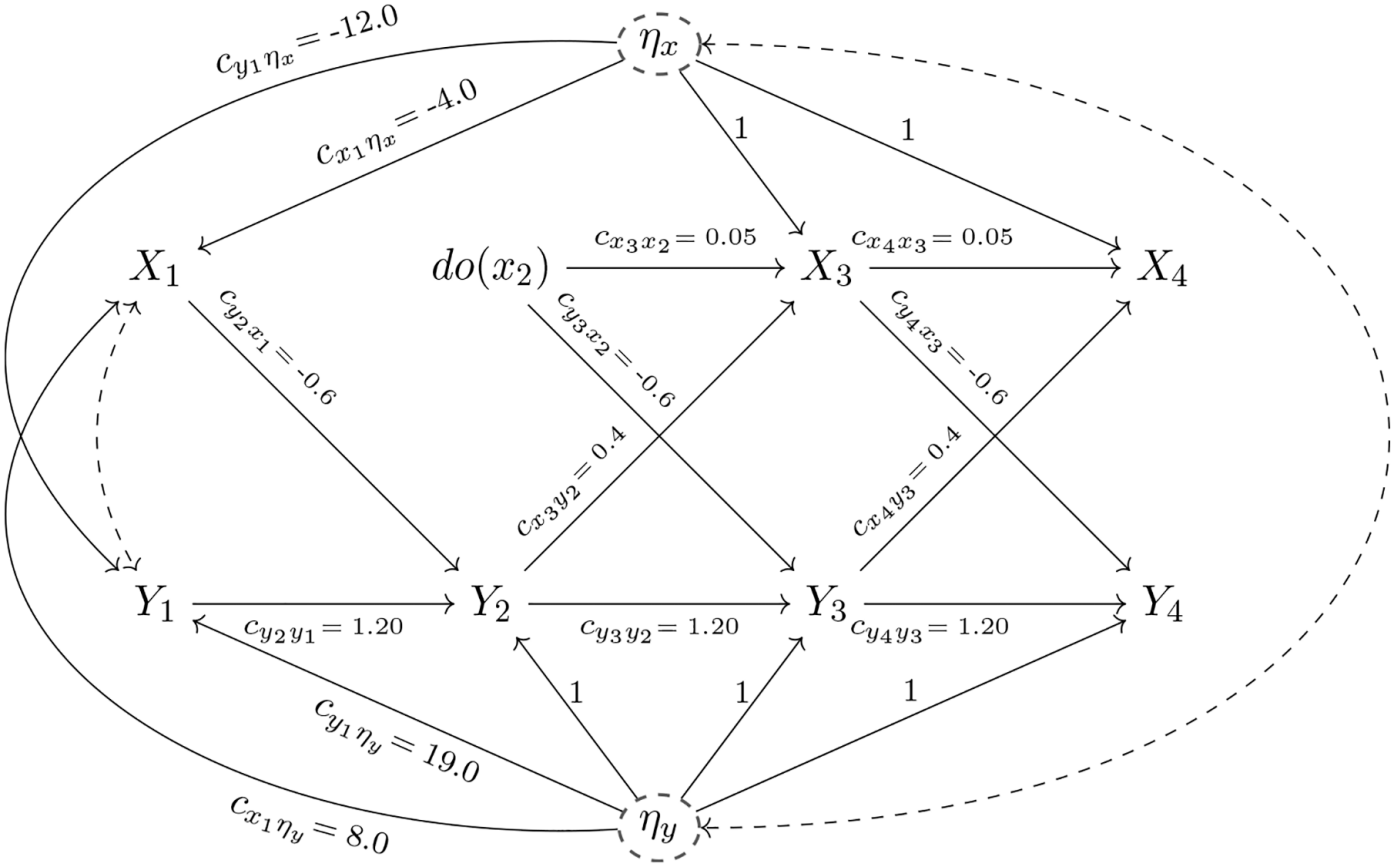
The causal diagram (DAG) from Figure 5 is modified to account for the intervention *do*(*x*_2_). The variable *X*_2_ has been replaced by *do*(*x*_2_) and all directed edges that formerly entered *X*_2_ have been removed, indicating that the interventional level *do*(*x*_2_) does not depend on *X*_1_, *Y*_1_ or *η*_*x*_ but is set by an experimenter (in our illustrative example we choose *do*(*X*_2_ = 26.30)). Note that all other variables and directed edges (causal arrows) remain unchanged as compared to the situation without the intervention *do*(*x*_2_) as depicted in Figure 5 reflecting the assumption of modularity.

As in the homogeneous model, we are interested in the effects of an intervention represented by the *do*-operator. Since the causal diagram of the model with random intercepts still belongs to the class of DAGs (see Figure 5), the *do*-operator is well-defined and can be directly applied to the model including random intercepts. Figure 6 displays the situation where the intervention *do*(*x*_2_) is applied.

The interventional distribution P(*Y*_3_|*do*(*x*_2_)) describes blood glucose levels in the entire population of individuals, given that the treatment *do*(*x*_2_) has been administered. The inclusion of random intercepts allows us to define the person-specific effects of interventions.

Each individual in the population can be characterized by the values *z*_*x*_ and *z*_*y*_ of his or her time-invariant characteristics *η*_*x*_ and *η*_*y*_. We define the person-specific interventional distribution of *Y*_3_ for an individual characterized by *η*_*x*_ = *z*_*x*_ and *η*_*y*_ = *z*_*y*_ as P(*Y*_3_|*do*(*x*_2_), *η*_*x*_ = *z*_*x*_, *η*_*y*_ = *z*_*y*_). The person-specific interventional distribution describes blood glucose levels at time *t* = 3 for an individual who is characterized by the time-invariant characteristics *η*_*x*_ = *z*_*x*_ and *η*_*y*_ = *z*_*y*_ after the treatment *do*(*x*_2_) has been applied. If multiple individuals have the same time-invariant characteristics the person-specific interventional distribution is also the subgroup-specific interventional distribution of this homogeneous group of individuals.

For both the interventional distribution P(*Y*_3_|*do*(*x*_2_)) and the person-specific interventional distribution P(*Y*_3_|*do*(*x*_2_), *η*_*x*_ = *z*_*x*_, *η*_*y*_ = *z*_*y*_), the random intercepts *η*_*x*_ and *η*_*y*_ belong to every adjustment set for backdoor identification (see Figure 5; Pearl (2009); Shpitser and Pearl (2006)). Thus, to apply backdoor adjustment in the model with random intercepts, the values of the latter need to be known. Recall that random intercepts are latent variables and thus unobserved (i.e., not part of the available data). As a consequence, the backdoor adjustment formulae cannot be applied straightforwardly.^8^ Causal identification can be established, however, by following the criteria for *linear* causal DAG-models suggested by Gische and Voelkle (2020) as explicated in more detail at the end of the section titled “Interventional Variance versus Conditional Variance.”

**Figure 7.**
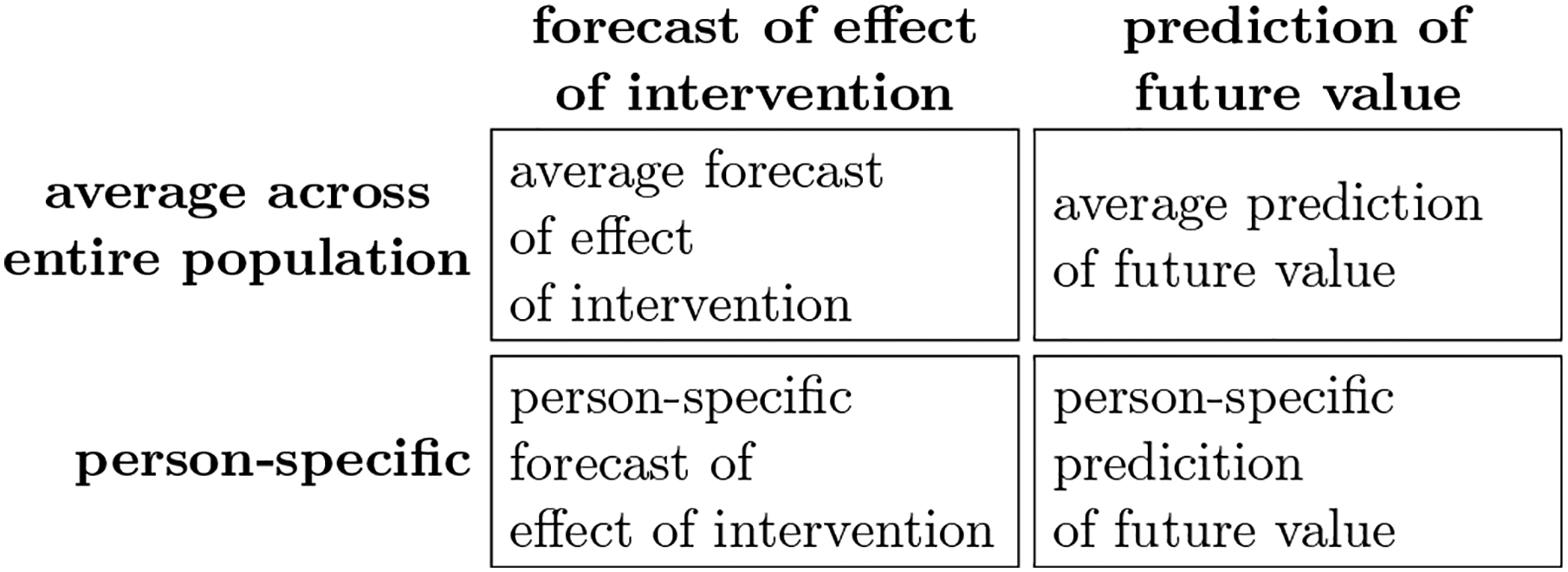
The four cells of the depicted (2 × 2) table display all possible combinations that result from the binary distinctions ‘forecast versus prediction’ (columns) and ‘average versus person-specific’ (rows).

In the first half of the paper, we analyzed a homogeneous population and focused on the difference between forecasts of effects of interventions and predictions of future values (cells in the first row in Figure 7). The introduction of between-person heterogeneity in the second half of the paper suggests an additional distinction: The difference between average values across the entire population and person-specific values. In the following, we focus on the difference between the average effects of interventions versus person-specific effects of interventions (cells in the first column in Figure 7). Space limitations preclude a discussion of predictions of future values in the presence of unobserved heterogeneity in the main text. A formal treatment of the differences between forecasts of effects of interventions and predictions of future values in the presence of unobserved heterogeneity is presented in the online supple mentary material.

In the following, we consider three hypothetical individuals from the population, namely Amy, Joe, and Sam, who are characterized by person-specific characteristics displayed in Table 6. We are interested in the blood glucose levels at time *t* = 3 after the intervention *do*(*X*_2_ = 26.30) has been applied, where 26.30 mcIU/ml is the standard deviation of blood insulin levels in the model with unobserved heterogeneity (recall that due to the inclusion of random intercepts into the model the standard deviation of blood insulin increased from 11.48 to 26.30.) In the following sections, we compute the person-specific effects of interventions for Amy, Joe, and Sam and compare these quantities to the average (unconditional) effect of an intervention in the entire population.

### Person-specific interventional mean and variance

The unconditional mean of blood glucose is the best prediction of blood glucose level at time *t* = 3 if no further information is available. Since all observable variables are mean-centered and the random intercepts *η*_*x*_ and *η*_*y*_ have zero means, the unconditional mean is equal to zero (see column one of Table 7). The unconditional variance in the presence of unobserved heterogeneity is equal to 3822.93 (see column 4 of Table 7) which is the variance of the prediction error when predicting *Y*_3_ in the absence of additional information.

The interventional mean and variance of *Y*_3_ describe the distribution of blood glucose levels at *t* = 3 in the entire population after the intervention *do*(*x*_2_) has been applied. They are given by the following formulae:

(6a) $$\text{E}\left(Y_{3}|\text{do}\left(x_{2}\right)\right)=c_{yx}x_{2}$$

(6b) $$\text{V}\left(Y_{3}|\text{do}\left(x_{2}\right)\right)={\left(c_{y_{1}\eta_{x}}c_{yy}^{2}+c_{x_{1}\eta_{x}}c_{yx}c_{yy}\right)}^{2}\psi_{\eta_{x}\eta_{x}}\;+{\left(c_{y_{1}\eta_{y}}c_{yy}^{2}+c_{x_{1}\eta_{y}}c_{yx}c_{yy}+c_{yy}+1\right)}^{2}\psi_{\eta_{y}\eta_{y}}\;+2\left(c_{y_{1}\eta_{x}}c_{yy}^{2}+c_{x_{1}\eta_{x}}c_{yx}c_{yy}\right)\left(c_{y_{1}\eta_{y}}c_{yy}^{2}+c_{x_{1}\eta_{y}}c_{yx}c_{yy}+c_{yy}+1\right)\psi_{\eta_{x}\eta_{y}}\;+c_{yx}^{2}c_{yy}^{2}\psi_{x_{1}x_{1}}+c_{yy}^{4}\psi_{y_{1}y_{1}}+2c_{yx}c_{yy}^{3}\psi_{x_{1}y_{1}}+\left(c_{yy}^{2}+1\right)\psi_{yy}$$

Equation (6a) reveals that the interventional mean in the presence of unobserved heterogeneity has the same linear functional form as in the homogeneous model (see Equation [2]). The first three lines of Equation (6b) are related to the random intercepts, whereas the last line is equal to the interventional variance in the homogeneous case (see Equation [4]). The value of V(*Y*_3_|*do*(*x*_2_)) in the presence of unobserved heterogeneity is equal to 5939.03 (see column 5 of Table 7), which is the variance of the forecast error when forecasting blood glucose levels at time *t* = 3 after the intervention *do*(*x*_2_) has been applied.

The person-specific interventional moments describe the distribution of blood glucose levels at *t* = 3 for an individual who is characterized by the time-invariant features *η*_*x*_ = *z*_*x*_ and *η*_*y*_ = *z*_*y*_, after the treatment *do*(*x*_2_) has been applied. The person-specific interventional mean is given by the following formula:

(7) $$\text{E}\left(Y_{3}|\text{do}\left(x_{2}\right),\eta_{x}=z_{x},\eta_{y}=z_{y}\right)\;=c_{yx}x_{2}+\left(c_{y_{1}\eta_{x}}c_{yy}^{2}+c_{x_{1}\eta_{x}}c_{yx}c_{yy}\right)z_{x}\;+\left(c_{y_{1}\eta_{y}}c_{yy}^{2}+c_{x_{1}\eta_{y}}c_{yx}c_{yy}+c_{yy}+1\right)z_{y}$$

Equation (7) reveals that the person-specific interventional mean is a function of the interventional level *x*_2_ and the time-invariant characteristics *z*_*x*_ and *z*_*y*_ of that person. For example, the person-specific interventional mean for Sam is computed by plugging in Sam’s numeric values $z_{x}^{\text{Sam}}=2.24$ and $z_{y}^{\text{Sam}}=3.16$ (see row 3 of Table 6) into the equation for the person-specific mean as stated in row 3 of Table 7:

(8) $$\text{E}\left(Y_{3}|\text{do}\left(X_{2}=26.30\right),\eta_{x}=z_{x}^{\text{Sam}},\eta_{y}=z_{y}^{\text{Sam}}\right)=\;-0.6\cdot 26.30-14.4\cdot 2.24+23.8\cdot 3.16=27.17$$

Likewise the person-specific interventional means for Amy (−58.73) and Joe (−15.78) can be computed. Thus, given the same treatment *do*(*X*_2_ = 26.30), different forecasts of blood glucose levels at time *t* = 3 are obtained for different individuals, depending on their respective time-invariant characteristics.

Note that the linear coefficient of the treatment level *x*_2_ is constant and equal to *c*_*yx*_ = −0.6 in the equation for the person-specific mean (see Equation [7] and column 3 of Table 7). As a consequence, the person-specific ATE is independent of the time-invariant characteristics of a person and constant across individuals. For example, the person-specific ATE of *X*_2_ on *Y*_3_ for Sam is given by:

(9) $$\text{E}\left(Y_{3}|\text{do}\left(X_{2}\right),\eta_{X}=z_{X}^{\text{Sam}},\eta_{y}=z_{y}^{\text{Sam}}\right)\;-\text{E}\left(Y_{3}|\text{do}\left(X_{2}^{\prime }\right),\eta_{X}=z_{X}^{\text{Sam}},\eta_{y}=z_{y}^{\text{Sam}}\right)\;=-0.6x_{2}-14.4z_{X}^{\text{Sam}}+23.8z_{y}^{\text{Sam}}\;-\left(-0.6x_{2}^{\prime }-14.4z_{X}^{\text{Sam}}+23.8z_{y}^{\text{Sam}}\right)\;=-0.6\left(x_{2}-x_{2}^{\prime }\right)$$

Equation (9) reveals that the ATE is independent of Sam’s time-invariant characteristics (i.e., $z_{x}^{\text{Sam}}$ and $z_{y}^{\text{Sam}}$ cancel out), a result that also holds for every other individual in the population.^9^ In other words, a *change* in the treatment level, say from *do*(*X*_2_ = *x*_2_) to $\text{do}\left(X_{2}=x_{2}^{\prime }\right)$, will cause the same *change* in forecasted values for each individual.

The difference between the (unconditional) interventional moments and the person-specific interventional moments can be illustrated as follows. A physician initially forecasts the value E(*Y*_3_|*do*(*X*_2_ = 26.30)) = −0.6 · 26.30 = −15.78 for a *new* patient under treatment of whom the person-specific characteristics are yet unknown. After learning that a patient is Amy, Joe, or Sam (and their respective person-specific characteristics) the physician updates his or her forecast to −58.73, −15.78, or 27.17, respectively. Note that by learning the person-specific characteristics of a new patient, the variance of the forecast error drops from 5939.03 to 951.43 (see columns 5 and 6 of Table 7).

### Person-specific probabilities of treatment success

As in the section titled “Interventional Probabilities versus Conditional Probabilities” we use the binary variable $Y_{3}^{*}$ to indicate $\left(Y_{3}^{*}=1\right)$ that blood glucose levels at time *t* = 3 are within an acceptable range [−40, 80] (mean-centered metric), an event we refer to as treatment success. Recall that $Y_{3}^{*}=0$ indicates the presence of hypo- or hypoglycemia, that is, blood glucose levels outside the acceptable range.

In the presence of unobserved heterogeneity, the (unconditional) probability of treatment success is equal to .52 given the treatment *do*(*X*_2_ = 26.30) (see column 1 of Table 8). That is, a physician would estimate a 52% probability of treatment success after the intervention *do*(*X*_2_ = 26.30) has been applied to a new patient from the heterogeneous population (a patient whose person-specific characteristics are yet unknown).

The right column of Table 8 contains the person-specific probabilities of treatment success for the three hypothetical individuals Amy, Joe, and Sam. After learning that a patient is Amy, Joe, or Sam (and their respective person-specific characteristics) the physician would update his or her initial beliefs about the probability of treatment success from 52% to 27%, 78%, or 94%, respectively. The reasons for this update are twofold. First, the person-specific means differ across the three individuals (see Equations [7] and [8]). Second, the amount of uncertainty when forecasting *Y*_3_ drops from 5939.03 to 951.43 after learning the person-specific characteristics (see columns 5 and 6 of Table 7).

**Figure 8.**
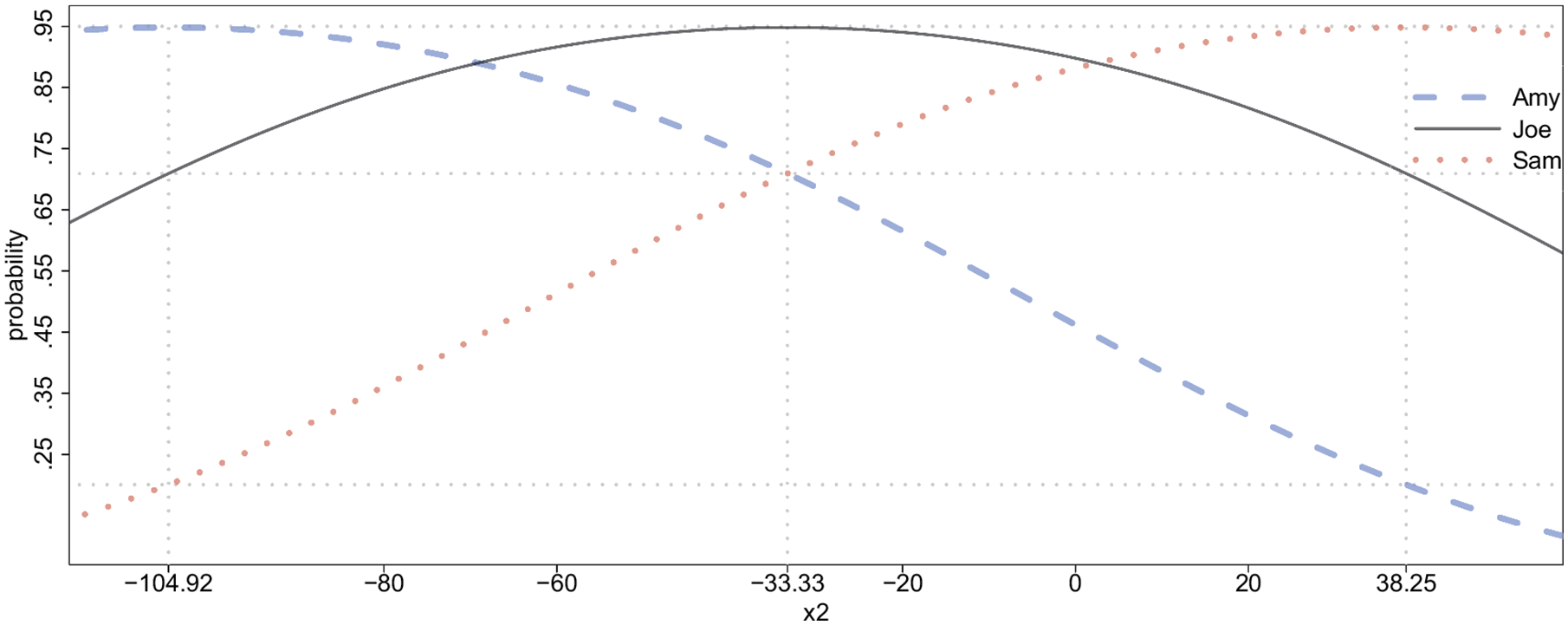
The person-specific interventional probabilities (vertical axis) are depicted for the three hypothetical individuals Amy (dashed line), Joe (solid line) and Sam (dotted line) as a function of the (mean-centered) interventional level (horizontal axis). Vertical-dotted lines are drawn at the interventional levels that maximize the respective probabilities of treatment success. For example, the interventional level *do*(*X*_2_ = 38.25) maximizes the probability of treatment success for Sam, which is 94.8%. A dotted horizontal line is drawn at .948 which is the probability of treatment success under optimal treatment. Two more dotted horizontal lines are drawn at .71 and .20 which correspond to person-specific probabilities of treatment success under different types of non-optimal treatment (see Table 6).

Figure 8 displays the person-specific probabilities of treatment success for Amy (dashed line), Joe (solid line) and Sam (dotted line) as a function of the interventional level (population mean-centered metric). Each curve has a unique maximum. Due to the simple nature of the model the three curves share the same maximal value of 94.8%.^10^ Note that the person-specific optimal treatment levels differ across persons: *do*(*X*_2_ = −104.92) for Amy, *do*(*X*_2_ = −33.33) for Joe, and *do*(*X*_2_ = 38.25) for Sam. In other words, if a given person is administered his or her individually optimal treatment level, the probability of treatment success for that person is 94.8%.

Joe is the average patient, that is, his person-specific characteristics coincide with the respective population means (see row 2 of Table 6). The person-specific optimal treatment level for Joe is −33.33 which is also the average optimal treatment level in the entire population. If a physician treats a *new* patient from the population for whom the person-specific characteristics are yet unknown, the optimal treatment decision would be *do*(*X*_2_ = −33.33). If the physician learns that the new patient is Joe (i.e., the average patient) the initial treatment *do*(*X*_2_ = −33.33) coincides with Joe’s person-specific optimal treatment resulting in a 94.8% probability of treatment success. On the other hand, if the new patient turns out to be Amy or Sam, then the treatment *do*(*X*_2_ = −33.33) is no longer optimal (given this new person-specific information) and would result in a 71% probability of treatment success in either case. The optimal treatment decision after learning that the new patient is Amy or Sam would be *do*(*X*_2_ = −104.92) or *do*(*X*_2_ = 38.25), respectively, resulting in a 94.8% probability of treatment success in either case. Thus, updating an initial population-based optimal treatment decision to include Amy’s or Sam’s person-specific characteristics, results in an absolute increase of 23.8% in the probability of treatment success.

## Discussion

In this article, we focused on the example of a four-wave linear cross-lagged panel design to provide a didactic presentation of Pearl’s (2009) approach to causal inference for researchers familiar with structural equation modeling.^11^ Throughout we emphasized the important distinction between (a) forecasting the future value of a variable following an intervention at a prior wave and (b) predicting the future value of a variable following measurement of variables at a prior wave. In task (a) a causal effect is computed based on the interventional distribution in a DAG-based framework. We focus on *do*(*x*_*t*_)-type interventions that set each individual’s value on the variable *X* at time *t* to a fixed constant value. In task (b) the future value of a variable is predicted using the conditional distribution (statistical quantity). Task (a) requires identification of the interventional distribution and its moments (causal quantities). Causal identification in the case of a homogeneous population can be established using the machinery of Pearl’s (2009) approach to causal inference which includes graphical criteria such as the backdoor criterion. Causal identification in case of a heterogeneous population can be established using a parametric procedure suggested by Gische and Voelkle (2020).

Our initial development of the approach assumed that all individuals were homogeneous. Later, we relaxed this assumption to account for unobserved heterogeneity (person-specific differences in the mean levels of the two variables) by including additive random intercepts into the linear cross-lagged panel framework. We showed that – even in this simple model – a treatment decision that is optimal on average can be improved substantially if person-specific characteristics can be incorporated into the model. In other words, treating a new, unknown patient with a treatment dose that has proven to be optimal on average (e.g., based on the results of randomized control trials) will only be optimal for a specific person as long as additional person-specific information is *not* available (Voelkle et al., 2018). As (a) relevant person-specific information becomes available or (b) repeated measures of the individual become available that allow us to control for time-invariant person characteristics, this information can be incorporated to develop a treatment level that more adequately matches the optimal level of treatment for the individual.

Pearl’s (2009) approach to DAG-based causal inference was developed using a general nonparametric framework. We used a linear parameterization of the cross-lagged panel framework to simplify the results and show the connection to linear structural equation modeling. The linear parameterization has weaknesses and strengths. On the one hand, if linear relationships represent a serious misspecification of the unknown true nonlinear functional form, the models will be misspecified and the results will be biased. On the other hand, the linear specification permits the inclusion of random intercepts, that is, person-specific differences in the mean levels of the time series, which reduces the risk of *causal* misspecification. In linear models with random intercepts additive time-invariant unobserved confounders are being statistically controlled. This, in turn, allows for the identification of causal effects that would *not* be identified in the most general non-parametric setup.^12^ In the case of linear models with random intercepts, we can to use the approach developed by Gische and Voelkle (2020) to establish identification of causal effects. The latter approach could also be used to obtain estimates of causal quantities in the presence of random intercepts.

### Current limitations and future directions

The methods presented in this paper permit causal conclusions to be reached based on a combination of observational data and assumptions about the data generating mechanism. The effects of a hypothetical *do*-intervention were forecasted without performing an experiment or otherwise perturbing the dynamic system. For causal conclusions based on observational data to be valid, the underlying assumptions need to hold. The methods discussed in this paper rely on the correct a priori specification of the causal ordering of the variables and the assumption of a linear dynamic process that is both stable over time and that is not altered by the intervention (modularity, autonomy). In the present article, we only allowed for between-person differences in the individual mean levels of the variables. Computing causal forecasts based on models that account for between-person differences in the autoregressive and cross-lagged effects remains a task for future research.

Statistical tests are available that can detect certain types of misspecification. In the present paper the set of possible causal orders is limited in part by the cross-lagged panel design (e.g., time ordering of variables). Further tests of the causal ordering of the variables (e.g., maximum lag of autoregressive effects) can be performed using procedures proposed by Chen et al. (2014), Shipley (2003), Thoemmes et al. (2018), and Tian and Pearl (2002). The assumption of autonomous mechanisms is in principle testable, but requires that both observational and experimental data on the process are available. The consequences of violations of non-testable assumptions can sometimes be gauged via sensitivity analyses and robustness checks (Ding & VanderWeele, 2016; Imai et al., 2010; Rosenbaum, 2011). Given that our developments were in the context of linear causal models, the linearity of the functional relations should be examined. Graphical checks utilizing lowess fits or splines commonly used in regression can be performed (e.g., Cohen et al., 2003; Suk et al., 2019). Formal statistical tests of linearity are available both for nested and non-nested models (Amemiya, 1985; Lee, 2007; Schumacker & Marcoulides, 1998).

In the development of our approach, we did not consider measurement error. Cole and Preacher (2014) have highlighted the serious bias that can arise from the failure to address measurement error in structural equation models. In our insulin-glucose example we would expect this problem to be minimal since instantaneous measures of blood insulin and blood glucose both have high precision. In cases in which only a single measure of each variable is available at each measurement wave, the bias can be reduced by using methods to correct the observed variables for measurement error (e.g., Fuller, 1987) or structural equation models with latent variables can be constructed that yield measurement error-free estimates. Our proposed approach to cross-lagged panel designs can potentially be extended to define, identify, and estimate causal effects between latent variables. Again, this is a task for future research.

Finally, our example was based on simulated data based on empirical findings reported by Ito et al. (1998). To simplify our didactic presentation of the approach, we ignored both possible contemporaneous correlations among the residuals of the insulin and glucose time-series as well as possible autocorrelations in the residuals for the blood insulin and blood glucose series. From a causal perspective, this means that we generated the data to reflect the absence of time-varying unobserved confounders in both the autoregressive and the cross-lagged relationships. With a real data example, these assumptions need to be checked, for example, by using the methods to detect certain types of misspecifications referred to in the second paragraph of this section.

In conclusion, the proposed method combines the desirable features of cross-lagged panel designs, a sound definition of causal quantities based on DAGs, and the flexibility of SEM for data analysis. We hope that our presentation facilitates the integration of linear SEM and DAG-based causal inference and enables researchers to better distinguish between tools for predictive and causal research questions.

## Supplementary Material

Supplemental Material [1]

## Figures and Tables

**Table 1.** Numeric values of parameters for the homogeneous population.

structural coefficients				variance-covariance parameters				
*c*_*xx*_	*c*_*xy*_	*c*_*yx*_	*c*_*yy*_	$\psi_{x_{1}x_{1}}$	$\psi_{y_{1}y_{1}}$	$\psi_{x_{1}y_{1}}$	*ψ*_*xx*_	*ψ*_*yy*_
0.05	0.40	−0.60	1.20	131.76	632.94	254.12	20.00	40.00

Note. The numeric values of the structural coefficients correspond to a stationary and ergodic bivariate time series for the insulin-glucose dynamics displayed in Figures 1 and 2. The variances $\left(\psi_{x_{1}x_{1}},\psi_{y_{1}y_{1}}\right)$ and covariance $\left(\psi_{x_{1}y_{1}}\right)$ of the initial variables correspond to the long-term equilibrium values of the insulin-glucose process.

**Table 2.** Mean forecast of effects of interventions vs. mean prediction of future values.

(a) intervention			(b) observation		
intervention	interventional mean	numeric	observation	conditional mean	numeric
*do*(∅)	E(*Y*_3_|*do*(∅))	0	∅	E(*Y*_3_|∅)	0
*do*(*x*_2_)	E(*Y*_3_|*do*(*x*_2_))	− 0.6*x*_2_	*X*_2_ = *x*_2_	E(*Y*_3_|*X*_2_ = *x*_2_)	1.71*x*_2_
*do*(*x*_2_), *do*(*y*_2_)	E(*Y*_3_|*do*(*x*_2_), *do*(*y*_2_))	− 0.6*x*_2_ + 1.2*y*_2_	*X* = *x*_2_*, Y*_2_ = *y*_2_	E(*Y*_3_|*X*_2_ = *x*_2_, *Y*_2_ = *y*_2_)	− 0.6*x*_2_ + 1.2*y*_2_

Note. The left part of Table 2 contains three different interventional means. In row one, no intervention is applied (*do*(∅)), in row two an intervention on blood insulin at *t* = 2 is applied (*do*(*x*_2_)), and in row three interventions on both blood insulin and blood glucose at *t* = 2 are applied (*do*(*x*_2_), *do*(*y*_2_)). The right part of Table 2 contains three different conditional means that are used for predictions of future values. In row one, we condition on the empty set ∅ (no prior observations available), in row two on the observation *X*_2_ = *x*_2_, and in row three on the observations *X*_2_ = *x*_2_ and *Y*_2_ = *y*_2_.

**Table 3.** Variance of forecasts (intervention) vs. variance of predictions (future values).

(a) intervention			(b) observation		
intervention	interventional variance	numeric	observation	conditional variance	numeric
*do*(∅)	V(*Y*_3_|*do*(∅))	632.93	∅	V(*Y*_3_|∅)	632.93
*do*(*x*_2_)	V(*Y*_3_|*do*(*x*_2_))	951.43	*X*_2_ = *x*_2_	V(*Y*_3_|*X*_2_ = *x*_2_)	245.71
*do*(*x*_2_), *do*(*y*_2_)	V(*Y*_3_|*do*(*x*_2_), *do*(*y*_2_))	40.00	*X* = *x*_2_*, Y*_2_ = *y*_2_	V(*Y*_3_|*X*_2_ = *x*_2_, *Y*_2_ = *y*_2_)	40.00

Note. The left part of Table 3 contains three different interventional variances used when forecasting the value of an outcome variable. In row one no intervention is applied (*do*(∅)), in row two an intervention on blood insulin at *t* = 2 is applied (*do*(*x*_2_)), and in row three interventions are applied on both blood insulin and blood glucose at *t* = 2 (*do*(*x*_2_), *do*(*y*_2_)). The right part of Table 3 contains three different conditional variances that are used for predictions of future values. In row one, we condition on the empty set ∅ (no prior information available), in row two on the observation *X*_2_ = *x*_2_, and in row three on the observations *X*_2_ = *x*_2_ and *Y*_2_ = *y*_2_.

**Table 4.** Interventional probabilities versus conditional probabilities.

(a) intervention			(b) observation		
intervention	interventional probability	numeric	observation	conditional probability	numeric
*do*(∅)	$\text{P}\left(Y_{3}^{*}=1|\text{do}(\emptyset )\right)$	.94	∅	$\text{P}\left(Y_{3}^{*}=1|\emptyset \right)$	.94
*do*(*x*_2_)	$\text{P}\left(Y_{3}^{*}=1|\text{do}\left(x_{2}\right)\right)$	.86	*X*_2_ = *x*_2_	$\text{P}\left(Y_{3}^{*}=1|X_{2}=X_{2}\right)$	.9999
*do*(*x*_2_), *do*(*y*_2_)	$\text{P}\left(Y_{3}^{*}=1|\text{do}\left(x_{2}\right),\text{do}\left(y_{2}\right)\right)$	≈ 1	*X*_2_ = *x*_2_*, Y*_2_ = *y*_2_	$\text{P}\left(Y_{3}^{*}=1|X_{2}=x_{2},Y_{2}=y_{2}\right)$	≈ 1

Note. The left part of the Table 4 contains three different interventional probabilities that are used to forecast outcome values after an intervention has been applied. In row one, no intervention is applied (*do*(∅)), in row two an intervention on blood insulin at *t* = 2 is applied (*do*(*x*_2_)) and in row three an intervention on both blood insulin and blood glucose at *t* = 2 is applied (*do*(*x*_2_), *do*(*y*_2_)). The right part of Table 4 contains three different conditional probabilities that are used for predictions. In row one, we condition on the empty set ∅ (no prior information available), in row two on the observation *X*_2_ = *x*_2_, and in row three on the observations *X*_2_ = *x*_2_ and *Y*_2_ = *y*_2_. We use the numerical values *x*_2_ = 11.48 and *y*_2_ = 0 for both interventional levels and observed values. $Y_{3}^{*}$ is an indicator variables that is equal to 1 if blood glucose levels fall within the acceptable range [−40, 80 at *t* = 3, and is equal to 0 otherwise.

**Table 5.** Numeric values of parameters related to random intercepts.

structural coefficients				variance-covariance parameters		
$c_{x_{1}\eta_{x}}$	$c_{y_{1}\eta_{y}}$	$c_{x_{1}\eta_{y}}$	$c_{y_{1}\eta_{x}}$	$\psi_{\eta_{x}\eta_{x}}$	$\psi_{\eta_{y}\eta_{y}}$	$\psi_{\eta_{x}\eta_{y}}$
−4.00	19.00	8	−12	5.00	10.00	2.50

Note. Parameters introduced to model unobserved person-specific differences in the mean levels (see Figures 5 and 6). The structural coefficients $c_{x_{1}\eta_{x}}$, $c_{y_{1}\eta_{y}}$, $c_{x_{1}\eta_{y}}$ and $c_{y_{1}\eta_{x}}$ correspond to the accumulated long-term effects of the random intercepts on insulin and glucose levels, respectively. The parameters $\psi_{\eta_{x}\eta_{x}}$, $\psi_{\eta_{y}\eta_{y}}$, and $\psi_{\eta_{x}\eta_{y}}$ represent the variance-covariance structure of the random intercepts.

**Table 6.** Person-specific characteristics for Amy, Joe, and Sam.

Person	*z*_*x*_	*z*_*y*_
Amy	$z_{x}^{\text{Amy}}=-\text{SD}\left(\eta_{x}\right)=-2.24$	$z_{y}^{\text{Amy}}=-\text{SD}\left(\eta_{y}\right)=-3.16$
Joe	$z_{x}^{\text{Joe}}=\text{E}\left(\eta_{x}\right)=0$	$z_{y}^{\text{Joe}}=\text{E}\left(\eta_{y}\right)=0$
Sam	$z_{x}^{\text{Sam}}=\text{SD}\left(\eta_{x}\right)=2.24$	$z_{y}^{\text{Sam}}=\text{SD}\left(\eta_{y}\right)=3.16$

Note. Table 6 displays the person-specific characteristics for three hypothetical individuals Amy, Joe, and Sam. Amy’s and Sam’s values are one standard deviation below and above the mean, respectively. Joe’s values are equal to the mean of the distribution of person-specific characteristics in the population.

**Table 7.** Interventional means and variances in models with unobserved heterogeneity.

Mean			Variance		
E(*Y*_3_)	E(*Y*_3_|*do*(*x*_2_))	E(*Y*_3_|*do*(*x*_2_), *η*_*x*_ = *z*_*x*_, *ηy* = *z*_*y*_)	V(*Y*_3_)	V(*Y*_3_|*do*(*x*_2_))	V(*Y*_3_|*do*(*x*_2_), *η*_*x*_ = *z*_*x*_, *ηy* = *z*_*y*_)
0	− 0.6*x*_2_	− 0.6*x*_2_ − 14.4*z*_*x*_ + 23.8*z*_*y*_	3822.93	5939.03	951.43

Note. The left part of Table 7 contains the unconditional mean (used for prediction of future values) and interventional means (used to forecast outcome values after an intervention has been applied). The right part of Table 7 contains the unconditional variance (prediction error) and interventional variances (forecast errors).

**Table 8.** Probabilities of treatment success in models with unobserved heterogeneity.

(unconditional) probability of treatment success	person-specific probabilities of treatment success
	$\text{P}\left(Y_{3}^{*}=1|\text{do}\left(X_{2}=26.30\right),\eta_{x}=z_{x}^{\text{Amy}},\eta_{y}=z_{y}^{\text{Amy}}\right)=.27$
$\text{P}\left(Y_{3}^{*}=1|\text{do}\left(X_{2}=26.30\right)\right)=.52$	$\text{P}\left(Y_{3}^{*}=1|\text{do}\left(X_{2}=26.30\right),\eta_{x}=z_{x}^{\text{Joe}},\eta_{y}=z_{y}^{\text{Joe}}\right)=.78$
	$\text{P}\left(Y_{3}^{*}=1|\text{do}\left(X_{2}=26.30\right),\eta_{x}=z_{x}^{\text{Sam}},\eta_{y}=z_{y}^{\text{Sam}}\right)=.94$

Note. $Y_{3}^{*}$ is an indicator variable that is equal to 1 if blood glucose levels at *t* = 3 fall inside the acceptable range [−40, 80] (treatment success), and is equal to 0 otherwise. The left part of the Table 8 contains the (unconditional) probability of treatment success after the treatment *do*(*X*_2_ = 26.30) has been applied. The right part of Table 8 contains person-specific probabilities of treatment success for three hypothetical individuals. Amy’s time-invariant values are one standard deviation below the mean, Joe’s values are exactly at the mean, and Sam’s values are one standard deviation above the mean.

## Footnotes

1 Sometimes the class of acyclical directed *mixed* graphs is used. Mixed refers to the fact that graphs in this class contain directed edges as well as bidirected edges (Kang & Tian, 2009; Richardson, 2003).

2 The complete set of model equations and distributional assumptions are provided in the online supplementary material.

3 The two assumptions of homogeneous individuals and stationarity over time allow us to make statements about either a single individual over time or a population of homogeneous individuals. Given these two assumptions, the results of between subjects and within subjects analyses of the data are theoretically expected to be identical (Molenaar, 2004). Complexity is introduced if the parameters of the model are allowed to vary across individuals, that is, a person is drawn from a population of heterogeneous individuals (see section on “Between-Person Heterogeneity”).

4 In its most general form, the interventional distribution is the multivariate joint distribution of *all* variables in the system given the intervention *do*(*x*_*t*_) is applied. For example, the joint interventional distribution for the system depicted in Figure 2 is given by P(*X*_1_, *Y*_1_, *X*_2_, *Y*_2_, *X*_3_, *Y*_3_, *X*_4_, *Y*_4_|*do*(*x*_2_)). In this paper, we restrict our attention to the *marginal* interventional distribution of a single outcome variable, for example, P(*Y*_3_|*do*(*x*_2_)).

5 An introduction to DAG-based causal identification written in a didactic style of presentation is provided by Pearl et al. (2016).

6 Note that we did *not* set up an additional model for the probability of treatment success. Instead, we analytically derived the functional form of the population quantity $\text{P}\left(Y_{3}^{*}=1|\text{do}\left(x_{2}\right)\right)$ for the model depicted in Figure 1 and Figure 2. The complete set of model equations for a homogeneous population and the explicit formula for the interventional probability are provided in the online supplementary material.

7 In case one or more time-invariant variables (e.g., a person’s sex) are explicitly included as predictors of, say, *X*_*t*_, *t* = 1, …, *T*, then *η*_*x*_ collects all *other* additive time-invariant factors that are not explicitly modeled (the omitted factors; e.g., marital status, genetic endowment) that determine the value of *X*_*t*_.

8 The causal quantities P(*Y*_3_|*do*(*x*_2_)) and P(*Y*_3_|*do*(*x*_2_), *η*_*x*_ = *z*_*x*_, *η*_*y*_ = *z*_*y*_) are not identified based on the DAG depicted in Figure 5 in the most general non-parametric DAG setup. One way to ensure identification is to impose further assumptions on the functional form of the structural equations (e.g., linearity).

9 This result is a consequence of modeling unobserved heterogeneity via additive random intercepts. To capture heterogeneity in the person-specific ATE we need more complex models (e.g., models including product terms).

10 More complex models (e.g., models allowing for person-specific differences in error term variances) would be needed to model individual differences in the probabilities of treatment success under optimal treatment. We will return to this limitation in the Discussion.

11 Technical details, formal derivations, and R-code for numerical computations are provided in the online supplementary material.

12 All models discussed in the paper belong to the class of DAGs. Random intercepts are by definition latent variables and are therefore unobserved. Therefore, many standard nonparametric adjustment formulae used to compute causal quantities can no longer be applied in the presence of random intercepts.

## Appendix on Initial Variables

In this Appendix, we provide a rationale for our treatment of initial variables and briefly discuss alternative approaches. We focus on the model depicted in Figure 5 that includes individual heterogeneity. The model depicted in Figure 1 can be viewed as a special case.

The initial variables *X*_1_ and *Y*_1_ in Figure 5 are (a) connected by a bidirected dashed edge, (b) have incoming directed edges from the random intercepts *η*_*x*_ and *η*_*y*_, and (c) the values of the structural coefficients corresponding to the directed edges are denoted by $c_{x_{1}\eta_{x}}$, $c_{x_{1}\eta_{y}}$, $c_{y_{1}\eta_{x}}$ and $c_{y_{1}\eta_{y}}$. All three choices (a), (b), and (c) rest on the key assumptions depicted in Figure A1 that address the dynamics of the insulin–glucose relationship *prior* to the first measurement wave. The DAG depicted in Figure A1 encodes the assumption, that the *causal structure* of the insulin glucose dynamics is time-stable. In other words, the dynamics prior to the first measurement (gray part) contains only autoregressive and cross-lagged effects of order one. Note that we do *not* assume that the *values* of the structural coefficients are necessarily time-stable prior to the first measurement wave as indicated by leaving out labels for the directed edges in the gray part of the graph.

Drawing (a) a bidirected edge among the initial variables in Figure 5 indicates the existence of unobserved confounders. Assuming that the dynamics have already been going on before the first measurement wave (as displayed in Figure A1) implies that the *X*_1_-*Y*_1_ relationship is confounded by previous insulin and glucose levels. Examples for such confounding paths are *X*_1_ ← *X*_0_ → *Y*_1_ or *X*_1_ ← *Y*_0_ → *Y*_1_.

Drawing (b) directed edges from the random intercepts to the initial variables can be justified as follows: Directed edges drawn from *η*_*x*_ to the insulin levels *X*_*t*_ reflect the assumption that the omitted person-specific time-invariant variables have direct causal effects on the insulin levels at time *t*. Since the timing of the first measurement wave was determined by factors outside of the system (e.g., grant money deadlines, availability of the lab) there is no reason to believe that *η*_*x*_ should not have a direct causal effect on the initial variable *X*_1_ in Figure 5. Including the past of the dynamic process prior to the first measurement (gray part in Figure A1) reveals that insulin level at time *t* = 1 does not have a special status in the ongoing *underlying real-world* process. The fact that we treat it differently in our *model* (e.g., we assign the structural coefficient $c_{x_{1}\eta_{x}}$ to the path *η*_*x*_ → *X*_1_ instead of restricting its value to be equal to one) only arises due to the timing of our measurements which is determined by factors independent of the insulin-glucose dynamics (see Singer & Willett, 2003). The same argument holds true for the directed edge from *η*_*y*_ to the glucose level *Y*_1_.

The directed edge from *η*_*y*_ to *X*_1_ in Figure 5 reflects the assumption that *η*_*y*_ has a direct causal effect on *X*_1_ *in the depicted model*.^13^ Note that the model in Figure 5 omits those parts of the insulin glucose dynamics that were going on prior to the initial measurement wave. The DAG in depicted in Figure A1 explicitly contains the entire past of the insulin-glucose dynamics (gray part). As a consequence, *η*_*y*_ has indirect effects on *X*_1_ (e.g., *η*_*y*_ → *Y*_0_ → *X*_1_, *η*_*y*_ → *Y*_−1_ → *X*_0_ → *X*_1_) in the model depicted in Figure A1. These indirect paths from Figure A1 translate to direct paths in the linear cross-lagged panel model with *T* = 4 measurement waves as depicted in Figure 5. The same argument holds true for the directed edge from *η*_*x*_ to the insulin levels *Y*_1_.

Statement (c) says that the values of the structural coefficients corresponding to the directed edges are denoted by $c_{x_{1}\eta_{x}}$, $c_{x_{1}\eta_{y}}$, $c_{y_{1}\eta_{x}}$ and $c_{y_{1}\eta_{y}}$ and are therefore not restricted to be equal to one. In practice, we recommend estimating the values of these coefficients from the data without imposing any restrictions. For our data simulation we used a particular set of values, namely $c_{x_{1}\eta_{x}}=-4.00$, $c_{x_{1}\eta_{y}}=8.00$, $c_{y_{1}\eta_{x}}=-12.00$ and $c_{y_{1}\eta_{y}}=19.00$, as depicted in Figures 5 and 6 (see online supplementary material for a derivation of these values).
