# Supplementary material for: Forecasting Causal Effects of Interventions versus Predicting Future Outcomes
Source: Struct Equ Modeling. Author manuscript; Available in PMC 2022 Apr 22. (PMC9030387; doi:10.1080/10705511.2020.1780598)
Supplement: Supplemental Material [1] [file NIHMS1794239-supplement-Supplemental_Material__1_.pdf]

Forecasting Causal Effects of Interventions versus Predicting Future Outcomes

–

Online Supplementary Material

Christian Gische[a], Stephen G. West[b], and Manuel C. Voelkle[ac]

[a] Department of Psychology, Humboldt University Berlin, Berlin, Germany.
[b] Department of Psychology, Arizona State University, Tempe, Arizona, USA.
[c] Max Planck Institute for Human Development, Berlin, Germany.

Contact:	Christian Gische
Email:	christian.gische@hu-berlin.de.
Address:	Humboldt-Universität zu Berlin<br>Lebenswissenschaftliche Fakultät<br>Institut für Psychologie<br>Unter den Linden 6<br>10099 Berlin.

This material supplements the following main text:

Gische, C., West, S. G., & Voelkle, M. C. (2020). Forecasting Causal Effects of Interventions Predicting Future Outcomes. *Structural Equation Modeling.*
https://doi.org/10.1080/10705511.2020.1780598

© 2020 The Author(s). Published with license by Taylor & Francis Group, LLC. This is an Open Access article distributed under the terms of the Creative Commons Attribution License (http://creativecommons.org/licenses/by/4.0/), which permits unrestricted use, distribution, and reproduction in any medium, provided the original work is properly cited.

Introduction

The goal of this online supplementary material is to provide a comprehensive and reproducible account of how the results presented in the main text were derived. Based on this account, the reader should be able to reproduce both the analytic and the numerical results. In the section on "Data Generating Mechanisms" we state the complete set of equations that represent the data generating mechanisms. In the section on "Derivation of the Interventional and the Conditional Distribution" we analytically derive the formulae of the interventional distribution and the conditional distribution using matrix notation. In the section titled "Formulae from the Main Text" we link these matrix expressions to the formulae displayed in the main text. By doing so we show that the scalar expressions from the main text are an alternative and equivalent representation of the the general matrix expressions. In the section titled "Population Values for Illustration" we illustrate briefly how the numerical parameter values used in the illustrative example were calculated. Finally, the section titled "Computer Code" provides computer syntax for the software packages R and Mathematica. This computer syntax enables readers to reproduce all numerical and analytic results from the main text and allows adapting the code to their own data.

For ease of presentation we will drop the time indices of the structural coefficients in the online supplementary material. For example, we will write c_{xx} instead of $c_{x_2x_1}$, $c_{x_3x_2}$, and $c_{x_4x_3}$ for the autoregressive coefficients of the X-series. Analogously, we will write c_{yy} for the autoregressive coefficients of the Y-series as well as c_{xy} and c_{yx} for the cross-lagged coefficients. Likewise, we will drop the time indices of the variance parameters for $t \geq 2$. We will write ψ_{xx} instead of $\psi_{x_2x_2}$, $\psi_{x_3x_3}$, and $\psi_{x_4x_4}$ and ψ_{yy} instead of $\psi_{y_2y_2}$, $\psi_{y_3y_3}$, and $\psi_{y_4y_4}$. Equations and Tables from the online supplementary material are labeled with S (e.g., Table S.1, Equation [S.1]) to distinguish them from those in the main text.

Data Generating Mechanisms

We first provide the equations for the data generating mechanism for the case of a homogeneous population in the section titled "Data Generating Mechanisms (Homogeneous Population)" before we move on to the case of a heterogeneous population in the section titled "Data Generating Mechanisms (Heterogeneous Population)".

Data Generating Mechanisms (Homogeneous Population)

The causal diagram (DAG) displayed in Figure 1 of the main text represents the data generating mechanisms that underlies a bivariate time series model of fixed length $T = 4$. The units of analysis are individuals. The corresponding equations are assumed to hold for each individual $i = 1, ..., N$ and are denoted as:

$$\begin{aligned}
X_{i1} &= \varepsilon_{xi1} \\
Y_{i1} &= \varepsilon_{yi1} \\
X_{i2} &= c_{xx}X_{i1} + c_{xy}Y_{i1} + \varepsilon_{xi2} \\
Y_{i2} &= c_{yx}X_{i1} + c_{yy}Y_{i1} + \varepsilon_{yi2} \\
X_{i3} &= c_{xx}X_{i2} + c_{xy}Y_{i2} + \varepsilon_{xi3} \\
Y_{i3} &= c_{yx}X_{i2} + c_{yy}Y_{i2} + \varepsilon_{yi3} \\
X_{i4} &= c_{xx}X_{i3} + c_{xy}Y_{i3} + \varepsilon_{xi4} \\
Y_{i4} &= c_{yx}X_{i3} + c_{yy}Y_{i3} + \varepsilon_{yi4}
\end{aligned} \tag{S.1}$$

In Equation (S.1) we use the subscript i to indicate that the units of analysis are persons and to clearly distinguish between quantities that vary across persons (values of variables and error terms) and those that are assumed not to vary across persons (structural coefficients). In the following we drop the person index i most of the time for ease of presentation. The model consists of 8 equations and can alternatively be denoted in matrix notation:

$$\mathbf{V} = \mathbf{C}\mathbf{V} + \boldsymbol{\varepsilon} \tag{S.2}$$

The 8×1 vectors $\mathbf{V}$ and $\boldsymbol{\varepsilon}$ contain the variables and the error terms, respectively:

$$\mathbf{V}^{\mathsf{T}} = (X_1, Y_1, X_2, Y_2, X_3, Y_3, X_4, Y_4) \tag{S.3a}$$

$$\boldsymbol{\varepsilon}^{\mathsf{T}} = (\varepsilon_{x1}, \varepsilon_{y1}, \varepsilon_{x2}, \varepsilon_{y2}, \varepsilon_{x3}, \varepsilon_{y3}, \varepsilon_{x4}, \varepsilon_{y4}) \tag{S.3b}$$

The 8×8 matrix of structural coefficients $\mathbf{C}$ and the 8×8 covariance matrix of the error terms $\boldsymbol{\Psi}$ are given by Equations (S.4) and (S.5), respectively:

$$\mathbf{C} = \begin{pmatrix} 0 & 0 & 0 & 0 & 0 & 0 & 0 & 0 \\ 0 & 0 & 0 & 0 & 0 & 0 & 0 & 0 \\ c_{xx} & c_{xy} & 0 & 0 & 0 & 0 & 0 & 0 \\ c_{yx} & c_{yy} & 0 & 0 & 0 & 0 & 0 & 0 \\ 0 & 0 & c_{xx} & c_{xy} & 0 & 0 & 0 & 0 \\ 0 & 0 & c_{yx} & c_{yy} & 0 & 0 & 0 & 0 \\ 0 & 0 & 0 & 0 & c_{xx} & c_{xy} & 0 & 0 \\ 0 & 0 & 0 & 0 & c_{yx} & c_{yy} & 0 & 0 \end{pmatrix} \tag{S.4}$$

$$\boldsymbol{\Psi} = \begin{pmatrix} \psi_{x_1x_1} & \psi_{x_1y_1} & 0 & 0 & 0 & 0 & 0 & 0 \\ \psi_{x_1y_1} & \psi_{y_1y_1} & 0 & 0 & 0 & 0 & 0 & 0 \\ 0 & 0 & \psi_{xx} & 0 & 0 & 0 & 0 & 0 \\ 0 & 0 & 0 & \psi_{yy} & 0 & 0 & 0 & 0 \\ 0 & 0 & 0 & 0 & \psi_{xx} & 0 & 0 & 0 \\ 0 & 0 & 0 & 0 & 0 & \psi_{yy} & 0 & 0 \\ 0 & 0 & 0 & 0 & 0 & 0 & \psi_{xx} & 0 \\ 0 & 0 & 0 & 0 & 0 & 0 & 0 & \psi_{yy} \end{pmatrix} \tag{S.5}$$

Given the assumptions of N random draws from the population and multivariate normal error terms, we obtain $\boldsymbol{\varepsilon}_i \overset{iid}{\sim} \text{Norm}(\mathbf{0}, \boldsymbol{\Psi})$, $i = 1, ..., N$, where *iid* stands for independent and identically distributed. The 9×1 vector of distinct, functionally unrelated and unknown model parameters is denoted as $\boldsymbol{\theta}$ and given by the following expression:

$$\boldsymbol{\theta}^{\mathsf{T}} = (c_{xx}, c_{xy}, c_{yx}, c_{yy}, \psi_{x_1x_1}, \psi_{y_1y_1}, \psi_{x_1y_1}, \psi_{xx}, \psi_{yy}) \tag{S.6}$$

Based on *iid* data that stems from the data generating mechanism and a correctly specified statistical model (i.e., a bivariate linear cross-lagged panel model with correlated initial variables), the parameter vector $\boldsymbol{\theta}$ can be estimated, for example, via maximum likelihood. Numerical quantities in the main text, the Appendix to the main text and this online supplementary material are calculated based on the parameter values displayed in Table S.2.

Data Generating Mechanisms (Heterogeneous Population)

The causal diagram (DAG) displayed in Figure 5 of the main text represents the data generating mechanisms that underlie a bivariate time series model of fixed length $T = 4$

with additive random intercepts. The units of analysis are individuals. The corresponding equations are assumed to hold for each individual $i = 1, ..., N$ and are denoted as:

$$\begin{aligned}
\eta_{xi} &= \varepsilon_{\eta_x i} \\
\eta_{yi} &= \varepsilon_{\eta_y i} \\
X_{i1} &= c_{x1\eta_x}\eta_{xi} + c_{x1\eta_y}\eta_{yi} + \varepsilon_{xi1} \\
Y_{i1} &= c_{y1\eta_x}\eta_{xi} + c_{y1\eta_y}\eta_{yi} + \varepsilon_{yi1} \\
X_{i2} &= c_{xx}X_{i1} + c_{xy}Y_{i1} + \eta_{xi} + \varepsilon_{xi2} \\
Y_{i2} &= c_{yx}X_{i1} + c_{yy}Y_{i1} + \eta_{yi} + \varepsilon_{yi2} \\
X_{i3} &= c_{xx}X_{i2} + c_{xy}Y_{i2} + \eta_{xi} + \varepsilon_{xi3} \\
Y_{i3} &= c_{yx}X_{i2} + c_{yy}Y_{i2} + \eta_{yi} + \varepsilon_{yi3} \\
X_{i4} &= c_{xx}X_{i3} + c_{xy}Y_{i3} + \eta_{xi} + \varepsilon_{xi4} \\
Y_{i4} &= c_{yx}X_{i3} + c_{yy}Y_{i3} + \eta_{yi} + \varepsilon_{yi4}
\end{aligned} \tag{S.7}$$

In Equation (S.7) we use the subscript i to indicate that the units of analysis are persons and to clearly distinguish between quantities that vary across persons (values of variables, random intercepts, and error terms) and those that are assumed not to vary across persons (structural coefficients). In the following we drop the person index i most of the time for ease of presentation. The model consists of 10 equations and extends the set of equations for the homogeneous population (see Equation [S.1]) by including an equation for each random intercept η_x and η_y (first two Equations in [S.7]). The system can alternatively be denoted in matrix notation:

$$\mathbf{V} = \mathbf{C}\mathbf{V} + \boldsymbol{\varepsilon} \tag{S.8}$$

The 10×1 vectors $\mathbf{V}$ and $\boldsymbol{\varepsilon}$ contain the variables and the error terms, respectively:

$$\mathbf{V}^{\mathsf{T}} = (\eta_x, \eta_y, X_1, Y_1, X_2, Y_2, X_3, Y_3, X_4, Y_4) \tag{S.9a}$$

$$\boldsymbol{\varepsilon}^{\mathsf{T}} = (\varepsilon_{\eta_x}, \varepsilon_{\eta_y}, \varepsilon_{x1}, \varepsilon_{y1}, \varepsilon_{x2}, \varepsilon_{y2}, \varepsilon_{x3}, \varepsilon_{y3}, \varepsilon_{x4}, \varepsilon_{y4}) \tag{S.9b}$$

The 10×10 matrix of structural coefficients $\mathbf{C}$ is given by:

$$\mathbf{C} = \left(\begin{array}{cc|cccccccc}
0 & 0 & 0 & 0 & 0 & 0 & 0 & 0 & 0 & 0 \\
0 & 0 & 0 & 0 & 0 & 0 & 0 & 0 & 0 & 0 \\
\hline
c_{x1\eta_x} & c_{x1\eta_y} & 0 & 0 & 0 & 0 & 0 & 0 & 0 & 0 \\
c_{y1\eta_x} & c_{y1\eta_y} & 0 & 0 & 0 & 0 & 0 & 0 & 0 & 0 \\
1 & 0 & c_{xx} & c_{xy} & 0 & 0 & 0 & 0 & 0 & 0 \\
0 & 1 & c_{yx} & c_{yy} & 0 & 0 & 0 & 0 & 0 & 0 \\
1 & 0 & 0 & 0 & c_{xx} & c_{xy} & 0 & 0 & 0 & 0 \\
0 & 1 & 0 & 0 & c_{yx} & c_{yy} & 0 & 0 & 0 & 0 \\
1 & 0 & 0 & 0 & 0 & 0 & c_{xx} & c_{xy} & 0 & 0 \\
0 & 1 & 0 & 0 & 0 & 0 & c_{yx} & c_{yy} & 0 & 0
\end{array}\right) \tag{S.10}$$

Note that the 8×8 submatrix in the lower right is equal to the matrix of structural coefficients in the homogeneous case (see Equation [S.4]). The 10×10 covariance matrix

of the error terms $\mathbf{\Psi}$ is given by:

$$\mathbf{\Psi} = \left(\begin{array}{cc|cccccccc} \psi_{\eta_x\eta_x} & \psi_{\eta_x\eta_y} & 0 & 0 & 0 & 0 & 0 & 0 & 0 & 0 \\ \psi_{\eta_x\eta_y} & \psi_{\eta_y\eta_y} & 0 & 0 & 0 & 0 & 0 & 0 & 0 & 0 \\ \hline 0 & 0 & \psi_{x_1x_1} & \psi_{x_1y_1} & 0 & 0 & 0 & 0 & 0 & 0 \\ 0 & 0 & \psi_{x_1y_1} & \psi_{y_1y_1} & 0 & 0 & 0 & 0 & 0 & 0 \\ 0 & 0 & 0 & 0 & \psi_{xx} & 0 & 0 & 0 & 0 & 0 \\ 0 & 0 & 0 & 0 & 0 & \psi_{yy} & 0 & 0 & 0 & 0 \\ 0 & 0 & 0 & 0 & 0 & 0 & \psi_{xx} & 0 & 0 & 0 \\ 0 & 0 & 0 & 0 & 0 & 0 & 0 & \psi_{yy} & 0 & 0 \\ 0 & 0 & 0 & 0 & 0 & 0 & 0 & 0 & \psi_{xx} & 0 \\ 0 & 0 & 0 & 0 & 0 & 0 & 0 & 0 & 0 & \psi_{yy} \end{array}\right) \tag{S.11}$$

Note that the 8×8 submatrix in the lower right is equal to the covariance matrix in the homogeneous case (see Equation [S.5]). Given the assumptions of N random draws from the population and multivariate normal error terms, we obtain $\boldsymbol{\varepsilon}_i \overset{iid}{\sim} \text{Norm}(\mathbf{0}, \mathbf{\Psi})$, $i = 1, ..., N$, where *iid* stands for independent and identically distributed. The 16×1 vector of distinct, functionally unrelated and unknown model parameters is denoted as $\boldsymbol{\theta}$ and given by the following expression:

$$\begin{aligned} \boldsymbol{\theta}^\intercal = (&c_{x_1\eta_x}, c_{x_1\eta_y}, c_{y_1\eta_x}, c_{y_1\eta_y}, c_{xx}, c_{xy}, c_{yx}, c_{yy}, \\ &\psi_{\eta_x\eta_x}, \psi_{\eta_y\eta_y}, \psi_{\eta_x\eta_y}, \psi_{x_1x_1}, \psi_{y_1y_1}, \psi_{x_1y_1}, \psi_{xx}, \psi_{yy}) \end{aligned} \tag{S.12}$$

Note that the parameter vector in Equation (S.12) contains the parameters from the situation of a homogeneous population (see Equation [S.6] and also row 2 of Table S.2) and additional parameters related to heterogeneity in the population (see row 1 of Table S.2). Thus, the model for a heterogeneous population is an extension of the model for a homogeneous population. Based on *iid* data that stems from the data generating mechanism and a correctly specified statistical model (i.e., a bivariate linear cross-lagged panel model with correlated initial variables and correlated additive random intercepts), the parameter vector $\boldsymbol{\theta}$ can be estimated, for example, via maximum likelihood. Numerical quantities in the main text, the Appendix to the main text and this online supplementary material are calculated based on the parameter values displayed in Table S.2.

Derivation of the Interventional and the Conditional Distribution

We introduce the general procedure for deriving an interventional distribution using matrix notation in the sections on “Interventional Distribution (Homogeneous Population)” and “Interventional Distribution (Heterogeneous Population)”. In the section titled “Conditional Distribution (Heterogeneous Population)” we state the conditional distribution in the model for a heterogeneous population.

The starting point is the reduced from equation for recursive linear SEM (Bollen, 1989):[1]

$$\mathbf{V} = \mathbf{C}\mathbf{V} + \boldsymbol{\varepsilon} \quad \Leftrightarrow \quad \mathbf{V} = (\mathbf{I}_n - \mathbf{C})^{-1}\boldsymbol{\varepsilon} \tag{S.13}$$

[1]Note that we use the same notation for the data generating mechanisms and the correctly specified statistical model (here: a linear structural equation model that corresponds to a bivariate linear cross-lagged panel model used for data analysis ($T = 4$; including additive random intercepts in case of a heterogeneous population).

https://doi.org/10.1080/10705511.2020.1780598

The matrix $\mathbf{I}_n$ is the $(n \times n)$-dimensional identity matrix. The joint distribution of the variables $\mathbf{V}$ follows from the reduced form representation, the distributional assumption $\boldsymbol{\varepsilon} \sim \text{Norm}(\mathbf{0}, \boldsymbol{\Psi})$, and a result on linear transformation of a multivariate normal random vector given by Rao (1973, pp. 183-184):

$$\mathbf{V} \sim \text{Norm}(\,\mathbf{0}\,,\, (\mathbf{I}_n - \mathbf{C})^{-1}\boldsymbol{\Psi}(\mathbf{I}_n - \mathbf{C})^{-1\intercal}\,) \tag{S.14}$$

We use the result on "interventional distribution for multivariate normal linear SCM" from Gische and Voelkle (2020) for the derivation of the interventional distribution. To this end we need to show that our illustrative model satisfies the setup stated in the definition on "interventions in linear SCM" in Gische and Voelkle (2020). This is done separately for the model for a homogeneous population and the model for a heterogeneous population in the following two sections.

Interventional Distribution (Homogeneous Population)

In the following step-by-step procedure we show that the model introduced in the section titled "Data Generating Mechanisms (Homogeneous Population)" satisfies the setup from the definition on "interventions in linear SCM" in Gische and Voelkle (2020):[2]

1. The set of interventional variables $\mathcal{X}$: In our illustration we perform a hypothetical intervention on X_2. Thus, the set of interventional variables contains only a single element and is given by $\mathcal{X} = \{X_2\}$.

2. The index set $\mathcal{I}$ of interventional variables: The interventional variable X_2 is the 3rd variable in (the causally ordered) vector $\mathbf{V}$ (see Equation [S.3a]). Thus, the index set of interventional variables is $\mathcal{I} = \{3\}$.

3. Set size K_x of the set of interventional variables: Here, the set size of the set of interventional variables is $K_x = |\mathcal{X}| = 1$ since we intervene on a single variable.

4. Since the system of equations contains a total number of $n = 8$ variables, we obtain the following terms for the non-interventional variables:

   (a) Set of non-interventional variables: $\{X_1, Y_1, Y_2, X_3, Y_3, X_4, Y_4\}$

   (b) Index set $\mathcal{N}$ of non-interventional variables: $\mathcal{N} = \{1, 2, 4, 5, 6, 7, 8\}$

   (c) Number of non-interventional variables: $n - K_x = 8 - 1 = 7$

5. The 8×7 matrix $\mathbf{1}_\mathcal{N}$ is defined to contain all 8-dimensional unit vectors with non-interventional indices. The 8×1 matrix $\mathbf{1}_\mathcal{I}$ is defined to contain all 8-dimensional unit vectors with interventional indices:

$$\mathbf{1}_\mathcal{N} = \begin{pmatrix} 1 & 0 & 0 & 0 & 0 & 0 & 0 \\ 0 & 1 & 0 & 0 & 0 & 0 & 0 \\ 0 & 0 & 0 & 0 & 0 & 0 & 0 \\ 0 & 0 & 1 & 0 & 0 & 0 & 0 \\ 0 & 0 & 0 & 1 & 0 & 0 & 0 \\ 0 & 0 & 0 & 0 & 1 & 0 & 0 \\ 0 & 0 & 0 & 0 & 0 & 1 & 0 \\ 0 & 0 & 0 & 0 & 0 & 0 & 1 \end{pmatrix} \quad , \quad \mathbf{1}_\mathcal{I} = \begin{pmatrix} 0 \\ 0 \\ 1 \\ 0 \\ 0 \\ 0 \\ 0 \\ 0 \end{pmatrix}$$

[2]Throughout this section some symbols are used following the notation used by Gische and Voelkle (2020). These symbols include $\mathcal{X}$, $\mathcal{I}$, K_x, $\mathcal{N}$, $\mathbf{1}_\mathcal{N}$, $\mathbf{1}_\mathcal{I}$, etc. Whenever a symbol is used for the first time we also briefly explain its meaning. For a detailed explanation of each symbol, the reader is referred to the original article.

6. The matrix $\mathbf{I}_{\mathcal{N}}$ is defined as a 8×8 identity matrix where all diagonal elements with interventional indices are set to zero:

$$\mathbf{I}_{\mathcal{N}} = \begin{pmatrix} 1 & 0 & 0 & 0 & 0 & 0 & 0 & 0 \\ 0 & 1 & 0 & 0 & 0 & 0 & 0 & 0 \\ 0 & 0 & 0 & 0 & 0 & 0 & 0 & 0 \\ 0 & 0 & 0 & 1 & 0 & 0 & 0 & 0 \\ 0 & 0 & 0 & 0 & 1 & 0 & 0 & 0 \\ 0 & 0 & 0 & 0 & 0 & 1 & 0 & 0 \\ 0 & 0 & 0 & 0 & 0 & 0 & 1 & 0 \\ 0 & 0 & 0 & 0 & 0 & 0 & 0 & 1 \end{pmatrix}$$

The result on "interventional distribution for multivariate normal linear SCM" from Gische and Voelkle (2020) states that the interventional distribution of $\mathbf{V}$ given the intervention $do(x_2)$, denoted as $\text{P}(\mathbf{V} \mid do(x_2))$, is given by:

$$\text{Norm}(\,(\mathbf{I}_n - \mathbf{I}_{\mathcal{N}}\mathbf{C})^{-1}\mathbf{1}_{\mathcal{I}} \cdot x_2\,,\,(\mathbf{I}_n - \mathbf{I}_{\mathcal{N}}\mathbf{C})^{-1}\mathbf{I}_{\mathcal{N}}\mathbf{\Psi}\mathbf{I}_{\mathcal{N}}(\mathbf{I}_n - \mathbf{I}_{\mathcal{N}}\mathbf{C})^{-1\intercal}\,) \tag{S.15}$$

Note that the interventional distribution $\text{P}(\mathbf{V} \mid do(x_2))$ is an 8-dimensional *singular* normal distribution with reduced rank 7. The reason for the reduced rank is that the 8×1 vector $\mathbf{V}$ contains the variable X_2 which is subject to the intervention. Given the intervention $do(x_2)$ the variable X_2 is no longer stochastic but *fixed* to the constant value x_2. As a consequence of the intervention X_2 has a variance equal to zero which results in the reduced rank of the interventional distribution of $\mathbf{V}$. Singular normal distributions belong to the family of normal distributions, but some matrix operations are not defined due to the singularity (e.g., the probability density function [pdf] of non-singular normal distributions contains the inverse of the covariance matrix which is not defined for singular normal distribution and consequently the pdf of singular normal distributions needs to be represented in a different way). One way to avoid working with singular normal distributions is to focus on the interventional distribution of those variables that are not themselves subject to intervention. The latter variables are referred to as non-interventional variables and are denoted by $\mathbf{V}_{\mathcal{N}}$. The interventional distribution $\text{P}(\mathbf{V}_{\mathcal{N}} \mid do(x_2))$ is given by:

$$\text{Norm}(\,\mathbf{1}_{\mathcal{N}}^{\intercal}(\mathbf{I}_n - \mathbf{I}_{\mathcal{N}}\mathbf{C})^{-1}\mathbf{1}_{\mathcal{I}} \cdot x_2\,,\,\mathbf{1}_{\mathcal{N}}^{\intercal}(\mathbf{I}_n - \mathbf{I}_{\mathcal{N}}\mathbf{C})^{-1}\mathbf{I}_{\mathcal{N}}\mathbf{\Psi}\mathbf{I}_{\mathcal{N}}(\mathbf{I}_n - \mathbf{I}_{\mathcal{N}}\mathbf{C})^{-1\intercal}\mathbf{1}_{\mathcal{N}}\,) \tag{S.16}$$

The following list highlights some of the most important results of the interventional distribution in linear models with multivariate normally distributed error terms:

- The interventional distribution is again multivariate normal.
- All results for the multivariate normal distribution apply. For example, marginal interventional and conditional interventional distributions are themselves normal and can be calculated based on established procedures.
- The mean vector of the interventional distribution is a function of the interventional level (x_2 appears in the expressions of the interventional mean).
- The covariance matrix of the interventional distribution is functionally independent of the interventional level (x_2 does *not* appear in the expressions for the interventional covariance matrix).

The marginal interventional distribution $\text{P}(Y_3 \mid do(x_2))$ is a univariate normal distribution. To obtain the mean and the variance of $\text{P}(Y_3 \mid do(x_2))$ one has to extract the 6th entry from $\text{E}(\mathbf{V} \mid do(x_2))$ and the $(6,6)$ entry from $\text{V}(\mathbf{V} \mid do(x_2))$, respectively. Such

extractions of entries from vectors and matrices can be obtained by multiplication with so-called selection matrices. Let $\hat{\boldsymbol{\imath}}_6$ denote the 8×1 unit vector with the entry 1 on the 6th position. Then, $Y_3 = \hat{\boldsymbol{\imath}}_6^\intercal \mathbf{V}$ holds and the resulting marginal interventional distribution $\mathrm{P}(Y_3 \mid do(x_2))$ is univariate normal with the following scalar mean and scalar variance:

$$\mathrm{E}(Y_3 \mid do(x_2)) = \hat{\boldsymbol{\imath}}_6^\intercal (\mathbf{I}_n - \mathbf{I}_{\mathcal{N}} \mathbf{C})^{-1} \mathbf{1}_{\mathcal{I}} \cdot x_2 \tag{S.17a}$$

$$\mathrm{V}(Y_3 \mid do(x_2)) = \hat{\boldsymbol{\imath}}_6^\intercal \mathbf{1}_{\mathcal{N}}^\intercal (\mathbf{I}_n - \mathbf{I}_{\mathcal{N}} \mathbf{C})^{-1} \mathbf{I}_{\mathcal{N}} \boldsymbol{\Psi} \mathbf{I}_{\mathcal{N}} (\mathbf{I}_n - \mathbf{I}_{\mathcal{N}} \mathbf{C})^{-1\intercal} \mathbf{1}_{\mathcal{N}} \hat{\boldsymbol{\imath}}_6 \tag{S.17b}$$

Equations (S.17) appear in the main text and are therefore restated as Equations (S.33) in the section titled "Formulae from the Main Text (Homogeneous Population)". The representations in Equations (S.17) and (S.33) are equivalent and differ only in notation: The former uses matrix notation whereas the latter uses only scalar expressions.[3]

In addition to the interventional distribution, the main text also displays conditional distributions and discusses some of the differences between these two types of distributions. Formulae used for the computation of conditional distributions (and moments thereof) are established results for the multivariate normal distribution (Rao, 1973) and are therefore not treated explicitly in this section.

Interventional Distribution (Heterogeneous Population)

In the following step-by-step procedure we show that the model from the section titled "Data Generating Mechanisms (Heterogeneous Population)" satisfies the setup from the definition on "interventions in linear SCM" in Gische and Voelkle (2020):

1. The set of interventional variables $\mathcal{X}$: In our illustration we perform a hypothetical intervention on X_2. Thus, the set of interventional variables contains only a single element and is given by $\mathcal{X} = \{X_2\}$.

2. The index set $\mathcal{I}$ of interventional variables: The interventional variable X_2 is the 5th variable in (the causally ordered) vector $\mathbf{V}$ (see Equation S.9a). Thus, the index set of interventional variables is $\mathcal{I} = \{5\}$.

3. Set size K_x: The set size of the set of interventional variables is $K_x = |\mathcal{X}| = 1$ since we intervene on a single variable.

4. Since the system of equations contains a total of $n = 10$ variables we obtain the following terms for the non-interventional variables:

   (a) Set of non-interventional variables: $\{\eta_x, \eta_y, X_1, Y_1, Y_2, X_3, Y_3, X_4, Y_4\}$

   (b) Index set $\mathcal{N}$ of non-interventional variables: $\mathcal{N} = \{1, 2, 3, 4, 6, 7, 8, 9, 10\}$

   (c) Number of non-interventional variables: $n - K_x = 10 - 1 = 9$

5. The 10×9 matrix $\mathbf{1}_{\mathcal{N}}$ is defined to contain all 10-dimensional unit vectors with non-interventional indices in $\mathcal{N}$. The 10×1 matrix $\mathbf{1}_{\mathcal{I}}$ is defined to contain all

[3] Each entry of the interventional mean $\mathrm{E}(\mathbf{V} \mid do(x_2))$ and the interventional covariance matrix $\mathrm{V}(\mathbf{V} \mid do(x_2))$ can be stated using scalar expressions in a similar way. Due to space restrictions we do not provide all these scalar expressions in the online supplementary material. The computer code in the section titled "Mathematica Code for Algebraic Derivations" produces analytic expressions for the entire joint interventional distribution that involve only scalars.

10-dimensional unit vectors with interventional indices in $\mathcal{I}$:

$$\mathbf{1}_{\mathcal{N}} = \begin{pmatrix} 1 & 0 & 0 & 0 & 0 & 0 & 0 & 0 & 0 \\ 0 & 1 & 0 & 0 & 0 & 0 & 0 & 0 & 0 \\ 0 & 0 & 1 & 0 & 0 & 0 & 0 & 0 & 0 \\ 0 & 0 & 0 & 1 & 0 & 0 & 0 & 0 & 0 \\ 0 & 0 & 0 & 0 & 0 & 0 & 0 & 0 & 0 \\ 0 & 0 & 0 & 0 & 1 & 0 & 0 & 0 & 0 \\ 0 & 0 & 0 & 0 & 0 & 1 & 0 & 0 & 0 \\ 0 & 0 & 0 & 0 & 0 & 0 & 1 & 0 & 0 \\ 0 & 0 & 0 & 0 & 0 & 0 & 0 & 1 & 0 \\ 0 & 0 & 0 & 0 & 0 & 0 & 0 & 0 & 1 \end{pmatrix} , \quad \mathbf{1}_{\mathcal{I}} = \begin{pmatrix} 0 \\ 0 \\ 0 \\ 0 \\ 1 \\ 0 \\ 0 \\ 0 \\ 0 \\ 0 \end{pmatrix}$$

6. The matrix $\mathbf{I}_{\mathcal{N}}$ is defined as a 10×10 identity matrix where all diagonal elements with non-interventional indices are set to zero:

$$\mathbf{I}_{\mathcal{N}} = \begin{pmatrix} 1 & 0 & 0 & 0 & 0 & 0 & 0 & 0 & 0 & 0 \\ 0 & 1 & 0 & 0 & 0 & 0 & 0 & 0 & 0 & 0 \\ 0 & 0 & 1 & 0 & 0 & 0 & 0 & 0 & 0 & 0 \\ 0 & 0 & 0 & 1 & 0 & 0 & 0 & 0 & 0 & 0 \\ 0 & 0 & 0 & 0 & 0 & 0 & 0 & 0 & 0 & 0 \\ 0 & 0 & 0 & 0 & 0 & 1 & 0 & 0 & 0 & 0 \\ 0 & 0 & 0 & 0 & 0 & 0 & 1 & 0 & 0 & 0 \\ 0 & 0 & 0 & 0 & 0 & 0 & 0 & 1 & 0 & 0 \\ 0 & 0 & 0 & 0 & 0 & 0 & 0 & 0 & 1 & 0 \\ 0 & 0 & 0 & 0 & 0 & 0 & 0 & 0 & 0 & 1 \end{pmatrix}$$

The result on "interventional distribution for multivariate normal linear SCM" from Gische and Voelkle (2020) states that the interventional distribution of $\mathbf{V}$ given the intervention $do(x_2)$, denoted as $\mathrm{P}(\mathbf{V} \mid do(x_2))$, is given by:

$$\mathrm{Norm}(\, (\mathbf{I}_n - \mathbf{I}_{\mathcal{N}}\mathbf{C})^{-1}\mathbf{1}_{\mathcal{I}} \cdot x_2 \,,\, (\mathbf{I}_n - \mathbf{I}_{\mathcal{N}}\mathbf{C})^{-1}\mathbf{I}_{\mathcal{N}}\mathbf{\Psi}\mathbf{I}_{\mathcal{N}}(\mathbf{I}_n - \mathbf{I}_{\mathcal{N}}\mathbf{C})^{-1\intercal} \,) \tag{S.18}$$

Similar to the case of the homogeneous population, the interventional distribution of the 10×1 vector $\mathbf{V}$ (i.e., the vector of *all* variables *including* the variable X_2 that is intervened on) is singular. Again, to avoid working with singular distributions we focus on the interventional distribution of non-interventional variables denoted as $\mathrm{P}(\mathbf{V}_{\mathcal{N}} \mid do(x_2))$ and given by:

$$\mathrm{Norm}(\, \mathbf{1}_{\mathcal{N}}^\intercal(\mathbf{I}_n - \mathbf{I}_{\mathcal{N}}\mathbf{C})^{-1}\mathbf{1}_{\mathcal{I}} \cdot x_2 \,,\, \mathbf{1}_{\mathcal{N}}^\intercal(\mathbf{I}_n - \mathbf{I}_{\mathcal{N}}\mathbf{C})^{-1}\mathbf{I}_{\mathcal{N}}\mathbf{\Psi}\mathbf{I}_{\mathcal{N}}(\mathbf{I}_n - \mathbf{I}_{\mathcal{N}}\mathbf{C})^{-1\intercal}\mathbf{1}_{\mathcal{N}} \,) \tag{S.19}$$

The marginal interventional distribution $\mathrm{P}(Y_3 \mid do(x_2))$ is a univariate normal distribution. To obtain the mean and the variance of $\mathrm{P}(Y_3 \mid do(x_2))$ one has to extract the 8th entry from $\mathrm{E}(\mathbf{V} \mid do(x_2))$ and the $(8, 8)$ entry from $\mathrm{V}(\mathbf{V} \mid do(x_2))$, respectively. Such extractions of entries from vectors and matrices can be obtained by multiplication with so-called selection matrices. Let $\hat{\boldsymbol{\imath}}_8$ denote the 10×1 unit vector with the entry 1 on the 8th position. Then, $Y_3 = \hat{\boldsymbol{\imath}}_8^\intercal \mathbf{V}$ holds and the resulting marginal interventional distribution $\mathrm{P}(Y_3 \mid do(x_2))$ is univariate normal with the following mean and variance:

$$\mathrm{E}(Y_3 \mid do(x_2)) = \hat{\boldsymbol{\imath}}_8^\intercal(\mathbf{I}_n - \mathbf{I}_{\mathcal{N}}\mathbf{C})^{-1}\mathbf{1}_{\mathcal{I}}x_2 \tag{S.20a}$$

$$\mathrm{V}(Y_3 \mid do(x_2)) = \hat{\boldsymbol{\imath}}_8^\intercal\mathbf{1}_{\mathcal{N}}^\intercal(\mathbf{I}_n - \mathbf{I}_{\mathcal{N}}\mathbf{C})^{-1}\mathbf{I}_{\mathcal{N}}\mathbf{\Psi}\mathbf{I}_{\mathcal{N}}(\mathbf{I}_n - \mathbf{I}_{\mathcal{N}}\mathbf{C})^{-1\intercal}\mathbf{1}_{\mathcal{N}}\hat{\boldsymbol{\imath}}_8 \tag{S.20b}$$

Equations (S.20) appear in the main text and are therefore restated as Equations (S.38) in the section titled "Formulae from the Main Text (Heterogeneous Population)". The

representations in Equations (S.20) and (S.38) are equivalent and differ only in notation: The former uses matrix notation whereas the latter uses only scalar expressions.[4]

The person-specific interventional distribution of Y_3 (see Equation [S.39]) is defined as a conditional interventional distribution: we (a) intervene on X_2, that is, we apply $do(x_2)$, and (b) condition on the person-specific characteristics $\eta_x = z_x$ and $\eta_y = z_y$.

Recall that the interventional distribution $\mathrm{P}(\mathbf{V}_{\mathcal{N}} \mid do(x_2))$ is multivariate normal. Therefore, the conditional interventional distribution can be obtained based on established results for multivariate normal distributions. These established results are based on partitioning the mean vector and the covariance matrix of the joint distribution (e.g., Rao, 1973). The starting point is the 9×1 vector of non-interventional variables $\mathbf{V}_{\mathcal{N}} \mid do(x_2)$ given the intervention $do(x_2)$. Recall, that this vector $\mathbf{V}_{\mathcal{N}}$ is given by $(\eta_x, \eta_y, X_1, Y_1, Y_2, X_3, Y_3, X_4, Y_4)^\intercal$, that is, it contains all variables except X_2 on which the intervention occurred. We partition the 9×1 mean vector as follows:

$$\mathrm{E}(\mathbf{V}_{\mathcal{N}} \mid do(x_2)) = \left(\frac{\mathrm{E}((\eta_x, \eta_y)^\intercal \mid do(x_2))}{\mathrm{E}((X_1, Y_1, Y_2, X_3, Y_3, X_4, Y_4)^\intercal \mid do(x_2))} \right) =: \begin{pmatrix} \boldsymbol{\mu}_1^\dagger \\ \boldsymbol{\mu}_2^\dagger \end{pmatrix} \quad \begin{matrix} 2 \times 1 \\ 7 \times 1 \end{matrix}$$

To simplify the notation in the following formulae we define the symbols $\boldsymbol{\mu}_1^\dagger$ and $\boldsymbol{\mu}_2^\dagger$ to denote interventional means. Similarly, the 9×9 covariance matrix of the interventional distribution $\mathrm{P}(\mathbf{V}_{\mathcal{N}} \mid do(x_2))$ is partitioned as follows:

$$\mathrm{V}(\mathbf{V}_{\mathcal{N}} \mid do(x_2)) = \left(\begin{array}{c|c} \mathrm{V}\left(\begin{pmatrix} \eta_x \\ \eta_y \end{pmatrix} \mid do(x_2)\right) & \mathrm{COV}\left(\begin{pmatrix} \eta_x \\ \eta_y \end{pmatrix}, \begin{pmatrix} X_1 \\ Y_1 \\ Y_2 \\ X_3 \\ Y_3 \\ X_4 \\ Y_4 \end{pmatrix} \mid do(x_2)\right) \\ \hline \mathrm{COV}\left(\begin{pmatrix} X_1 \\ Y_1 \\ Y_2 \\ X_3 \\ Y_3 \\ X_4 \\ Y_4 \end{pmatrix}, \begin{pmatrix} \eta_x \\ \eta_y \end{pmatrix} \mid do(x_2)\right) & \mathrm{V}\left(\begin{pmatrix} X_1 \\ Y_1 \\ Y_2 \\ X_3 \\ Y_3 \\ X_4 \\ Y_4 \end{pmatrix} \mid do(x_2)\right) \end{array} \right) \tag{S.21}$$

Again, for simplicity of notation, we introduce the symbols $\boldsymbol{\Sigma}_{11}^\dagger$, $\boldsymbol{\Sigma}_{12}^\dagger$, $\boldsymbol{\Sigma}_{21}^\dagger$, and $\boldsymbol{\Sigma}_{22}^\dagger$ to refer to the corresponding entries of the partitioned interventional covariance matrix in Equation (S.21). Using this compact notation, the partitioned interventional covariance matrix from Equation (S.21) can be denoted as:

$$\mathrm{V}(\mathbf{V}_{\mathcal{N}} \mid do(x_2)) =: \left(\begin{array}{c|c} \boldsymbol{\Sigma}_{11}^\dagger & \boldsymbol{\Sigma}_{12}^\dagger \\ \hline \boldsymbol{\Sigma}_{21}^\dagger & \boldsymbol{\Sigma}_{22}^\dagger \end{array} \right) \quad , \quad \underbrace{\begin{array}{c|c} 2 \times 2 & 2 \times 7 \\ \hline 7 \times 2 & 7 \times 7 \end{array}}_{9 \times 9} \tag{S.22}$$

Based on the partitioned first- and second-order moments as stated above, the conditional interventional distribution $\mathrm{P}((X_1, Y_1, Y_2, X_3, Y_3, X_4, Y_4)^\intercal \mid do(x_2), \eta_x = z_x, \eta_y =$

[4] Each entry of the interventional mean $\mathrm{E}(\mathbf{V} \mid do(x_2))$ and the interventional covariance matrix $\mathrm{V}(\mathbf{V} \mid do(x_2))$ can be stated using scalar expressions in a similar way. We do not provide these scalar expressions in the online supplementary material. The computer code in section titled "Mathematica Code for Algebraic Derivations" produces analytic expressions for the entire joint interventional distribution that involve only scalars.

z_y) is multivariate normal with the following moments (e.g., Rao, 1973):

$$\mathrm{E}(\begin{pmatrix} X_1 \\ Y_1 \\ Y_2 \\ X_3 \\ Y_3 \\ X_4 \\ Y_4 \end{pmatrix} \mid do(x_2), \eta_x = z_x, \eta_y = z_y) = \boldsymbol{\mu}_2^\dagger - \boldsymbol{\Sigma}_{21}^\dagger \boldsymbol{\Sigma}_{11}^{\dagger -1} (\mathbf{z} - \boldsymbol{\mu}_1^\dagger) \tag{S.23a}$$

$$\mathrm{V}(\begin{pmatrix} X_1 \\ Y_1 \\ Y_2 \\ X_3 \\ Y_3 \\ X_4 \\ Y_4 \end{pmatrix} \mid do(x_2), \eta_x = z_x, \eta_y = z_y) = \boldsymbol{\Sigma}_{22}^\dagger - \boldsymbol{\Sigma}_{21}^\dagger \boldsymbol{\Sigma}_{11}^{\dagger -1} \boldsymbol{\Sigma}_{12}^\dagger \tag{S.23b}$$

Where $\mathbf{z} = (z_x, z_y)^\intercal$. The person-specific interventional distribution $\mathrm{P}(Y_3 \mid do(x_2), \eta_x = z_x, \eta_y = z_y)$ is defined in the main text. Therefore the matrix expressions in Equations (S.23) are restated in Equations (S.39). Note that the person-specific interventional mean $\mathrm{E}(Y_3 \mid do(x_2), \eta_x = z_x, \eta_y = z_y)$ is the 5th entry of the mean expression in Equation (S.23a) and the person-specific interventional variance $\mathrm{V}(Y_3 \mid do(x_2), \eta_x = z_x, \eta_y = z_y)$ is the $(5, 5)$th entry of the covariance expression in Equation (S.23b).

Conditional Distribution (Heterogeneous Population)

The *conditional* distributions of blood glucose levels at time $t = 3$ are *not* discussed in the main text for the model for a heterogeneous population.[5] In this section we state two conditional distributions from the model for a heterogeneous population, namely $\mathrm{P}(Y_3 \mid X_2 = x_2)$ and $\mathrm{P}(Y_3 \mid \eta_x = z_x, \eta_y = z_y, X_2 = x_2)$. These conditional distributions are calculated according to established results for multivariate normal distributions (e.g., Rao, 1973). We briefly compare these *conditional* distributions with the respective *interventional* distributions $\mathrm{P}(Y_3 \mid do(x_2))$ and $\mathrm{P}(Y_3 \mid do(x_2), \eta_x = z_x, \eta_y = z_y)$ that were derived in the previous section.

The model implied normal distribution in the illustrative example model (heterogeneous population) is stated in Equation (S.14). As a consequence of multivariate normality, the mean and variance of the conditional distribution $\mathrm{P}(Y_3 \mid X_2 = x_2)$ can be obtained via linear regression and are given by:

$$\mathrm{E}(Y_3 \mid X_2 = x_2) = \frac{\mathrm{COV}(X_2, Y_3)}{\mathrm{V}(X_2)} x_2 \tag{S.24a}$$

$$\mathrm{V}(Y_3 \mid X_2 = x_2) = \mathrm{V}(Y_3) - \frac{\mathrm{COV}(X_2, Y_3)^2}{\mathrm{V}(X_2)} \tag{S.24b}$$

The variances and covariances that appear on the right-hand side of Equations (S.24), namely $\mathrm{V}(X_2)$, $\mathrm{V}(Y_3)$, and $\mathrm{COV}(X_2, Y_3)$ correspond to the $(5, 5)$, $(8, 8)$, and $(5, 8)$ entry of the model-implied covariance matrix from Equation (S.14), respectively. These

[5]Instead, the text focuses on the comparison of two *interventional* distributions, namely the unconditional interventional distribution $\mathrm{P}(Y_3 \mid do(x_2))$ and the person-specific interventional distribution $\mathrm{P}(Y_3 \mid do(x_2), \eta_x = z_x, \eta_y = z_y)$.

variances and covariances are given as:

$$
\begin{aligned}
&\mathrm{V}(X_2) && \text{(S.25a)}\\
&= (1 + c_{x_1\eta_x}c_{xx} + c_{xy}c_{y_1\eta_x})^2\psi_{\eta_x\eta_x} + 2(1 + c_{x_1\eta_x}c_{xx} + c_{xy}c_{y_1\eta_x})(c_{x_1\eta_y}c_{xx} + c_{xy}c_{y_1\eta_y})\psi_{\eta_x\eta_y}\\
&+ (c_{x_1\eta_y}c_{xx} + c_{xy}c_{y_1\eta_y})^2\psi_{\eta_y\eta_y} + c_{xx}^2\psi_{x_1x_1} + 2c_{xx}c_{xy}\psi_{x_1y_1} + c_{xy}^2\psi_{y_1y_1} + \psi_{xx}\\
&\mathrm{V}(Y_3) && \text{(S.25b)}\\
&= \left(c_{yx} + c_{x_1\eta_x}c_{xx}c_{yx} + c_{xy}c_{y_1\eta_x}c_{yx} + c_{x_1\eta_x}c_{yx}c_{yy} + c_{y_1\eta_x}c_{yy}^2\right)^2\psi_{\eta_x\eta_x}\\
&+ 2\left(c_{yx} + c_{x_1\eta_x}c_{xx}c_{yx} + c_{xy}c_{y_1\eta_x}c_{yx} + c_{x_1\eta_x}c_{yx}c_{yy} + c_{y_1\eta_x}c_{yy}^2\right)\\
&\times\left(1 + c_{x_1\eta_y}c_{xx}c_{yx} + c_{xy}c_{y_1\eta_y}c_{yx} + c_{yy} + c_{x_1\eta_y}c_{yx}c_{yy} + c_{y_1\eta_y}c_{yy}^2\right)\psi_{\eta_x\eta_y}\\
&+ \left(1 + c_{x_1\eta_y}c_{xx}c_{yx} + c_{xy}c_{y_1\eta_y}c_{yx} + c_{yy} + c_{x_1\eta_y}c_{yx}c_{yy} + c_{y_1\eta_y}c_{yy}^2\right)^2\psi_{\eta_y\eta_y}\\
&+ (c_{xx}c_{yx} + c_{yx}c_{yy})^2\psi_{x_1x_1} + 2(c_{xx}c_{yx} + c_{yx}c_{yy})\left(c_{xy}c_{yx} + c_{yy}^2\right)\psi_{x_1y_1}\\
&+ \left(c_{xy}c_{yx} + c_{yy}^2\right)^2\psi_{y_1y_1} + c_{yx}^2\psi_{xx} + \left(1 + c_{yy}^2\right)\psi_{yy}\\
&\mathrm{COV}(X_2, Y_3) && \text{(S.25c)}\\
&= (1 + c_{x_1\eta_x}c_{xx} + c_{xy}c_{y_1\eta_x})\left(c_{yx} + c_{x_1\eta_x}c_{xx}c_{yx} + c_{xy}c_{y_1\eta_x}c_{yx} + c_{x_1\eta_x}c_{yx}c_{yy} + c_{y_1\eta_x}c_{yy}^2\right)\psi_{\eta_x\eta_x}\\
&+ \Bigg((c_{x_1\eta_y}c_{xx} + c_{xy}c_{y_1\eta_y})\left(c_{yx} + c_{x_1\eta_x}c_{xx}c_{yx} + c_{xy}c_{y_1\eta_x}c_{yx} + c_{x_1\eta_x}c_{yx}c_{yy} + c_{y_1\eta_x}c_{yy}^2\right)\\
&+ (1 + c_{x_1\eta_x}c_{xx} + c_{xy}c_{y_1\eta_x})\left(1 + c_{x_1\eta_y}c_{xx}c_{yx} + c_{xy}c_{y_1\eta_y}c_{yx} + c_{yy} + c_{x_1\eta_y}c_{yx}c_{yy} + c_{y_1\eta_y}c_{yy}^2\right)\Bigg)\psi_{\eta_x\eta_y}\\
&+ (c_{x_1\eta_y}c_{xx} + c_{xy}c_{y_1\eta_y})\left(1 + c_{x_1\eta_y}c_{xx}c_{yx} + c_{xy}c_{y_1\eta_y}c_{yx} + c_{yy} + c_{x_1\eta_y}c_{yx}c_{yy} + c_{y_1\eta_y}c_{yy}^2\right)\psi_{\eta_y\eta_y}\\
&+ c_{xx}(c_{xx}c_{yx} + c_{yx}c_{yy})\psi_{x_1x_1} + \left(c_{xy}(c_{xx}c_{yx} + c_{yx}c_{yy}) + c_{xx}\left(c_{xy}c_{yx} + c_{yy}^2\right)\right)\psi_{x_1y_1}\\
&+ c_{xy}\left(c_{xy}c_{yx} + c_{yy}^2\right)\psi_{y_1y_1} + c_{yx}\psi_{xx}
\end{aligned}
$$

To calculate the conditional mean $\mathrm{E}(Y_3 \mid X_2 = x_2)$, one has to insert Equations (S.25) into Equation (S.24a). By doing so one obtains a lengthy fraction that cannot be simplified appreciably. The most important insight for our discussion is that the resulting term for the conditional mean is *not* equal to the interventional mean $\mathrm{E}(Y_3 \mid do(x_2)) = c_{yx}x_2$ as stated in (S.38a). Using the numeric parameter values from Table S.2 one obtains $\mathrm{E}(Y_3 \mid X_2 = x_2) = 2.25x_2$ for the conditional mean and $\mathrm{E}(Y_3 \mid do(x_2)) = -0.6x_2$ for the interventional mean.

Likewise, the conditional variance $\mathrm{V}(Y_3 \mid X_2 = x_2)$ is obtained by inserting Equations (S.25) into Equation (S.24b). Again a lengthy fraction is obtained which cannot be simplified appreciably. The resulting term for the conditional variance is *not* equal to the interventional variance from Equation (S.38b). Using the numeric parameter values from Table S.2 one obtains $\mathrm{V}(Y_3 \mid X_2 = x_2) = 323.52$ for the conditional variance and $\mathrm{V}(Y_3 \mid do(x_2)) = 5939.03$ for the interventional variance.

As a consequence of multivariate normality, the mean and variance of the conditional distribution $\mathrm{P}(Y_3 \mid \eta_x = z_x, \eta_y = z_y, X_2 = x_2)$ can be obtained via linear regression. The conditional mean $\mathrm{E}(Y_3 \mid \eta_x = z_x, \eta_y = z_y, X_2 = x_2)$ is a linear function of z_x, z_y, and x_2. In other words, $\mathrm{E}(Y_3 \mid \eta_x = z_x, \eta_y = z_y, X_2 = x_2) = a_{x_2}x_2 + a_{z_x}z_x + a_{z_y}z_y$, where the

linear coefficients take the following values:[6]

$$
\begin{aligned}
a_{x_2} = {} & \frac{1}{c_{xx}^2\psi_{x_1x_1} + 2c_{xx}c_{xy}\psi_{x_1y_1} + c_{xy}^2\psi_{y_1y_1} + \psi_{xx}} \\
& \times \Big(\left(c_{xx}^2 c_{yx} + c_{xx}c_{yx}c_{yy}\right)\psi_{x_1x_1} + \left(2c_{xx}c_{xy}c_{yx} + c_{xy}c_{yx}c_{yy} + c_{xx}c_{yy}^2\right)\psi_{x_1y_1} \\
& + \left(c_{xy}^2 c_{yx} + c_{xy}c_{yy}^2\right)\psi_{y_1y_1} + c_{yx}\psi_{xx} \Big)
\end{aligned} \tag{S.26a}
$$

$$
\begin{aligned}
a_{z_x} = {} & \frac{1}{c_{xx}^2\psi_{x_1x_1} + 2c_{xx}c_{xy}\psi_{x_1y_1} + c_{xy}^2\psi_{y_1y_1} + \psi_{xx}} \\
& \times \Big(\left(-c_{xx}(1 + c_{xy}c_{y_1\eta_x})c_{yx}c_{yy} + c_{xx}^2 c_{y_1\eta_x}c_{yy}^2\right)\psi_{x_1x_1} \\
& + \left(-c_{xy}c_{yx}c_{yy} + c_{x_1\eta_x}c_{xx}c_{xy}c_{yx}c_{yy} - c_{xy}^2 c_{y_1\eta_x}c_{yx}c_{yy} - c_{xx}c_{yy}^2 - c_{x_1\eta_x}c_{xx}^2 c_{yy}^2 + c_{xx}c_{xy}c_{y_1\eta_x}c_{yy}^2\right)\psi_{x_1y_1} \\
& + \left(c_{x_1\eta_x}c_{xy}^2 c_{yx}c_{yy} - c_{xy}c_{yy}^2 - c_{x_1\eta_x}c_{xx}c_{xy}c_{yy}^2\right)\psi_{y_1y_1} + \left(c_{x_1\eta_x}c_{yx}c_{yy} + c_{y_1\eta_x}c_{yy}^2\right)\psi_{xx} \Big)
\end{aligned} \tag{S.26b}
$$

$$
\begin{aligned}
a_{z_y} = {} & \frac{1}{c_{xx}^2\psi_{x_1x_1} + 2c_{xx}c_{xy}\psi_{x_1y_1} + c_{xy}^2\psi_{y_1y_1} + \psi_{xx}} \\
& \times \Big(\left(c_{xx}^2 + c_{xx}^2 c_{yy} - c_{xx}c_{xy}c_{y_1\eta_y}c_{yx}c_{yy} + c_{xx}^2 c_{y_1\eta_y}c_{yy}^2\right)\psi_{x_1x_1} \\
& + \left(2c_{xx}c_{xy} + 2c_{xx}c_{xy}c_{yy} + c_{x_1\eta_y}c_{xx}c_{xy}c_{yx}c_{yy} - c_{xy}^2 c_{y_1\eta_y}c_{yx}c_{yy} - c_{x_1\eta_y}c_{xx}^2 c_{yy}^2 + c_{xx}c_{xy}c_{y_1\eta_y}c_{yy}^2\right)\psi_{x_1y_1} \\
& + \left(c_{x_1\eta_y}c_{xy}^2 c_{yx}c_{yy} - c_{x_1\eta_y}c_{xx}c_{xy}c_{yy}^2 + c_{xy}^2(1 + c_{yy})\right)\psi_{y_1y_1} + \left(1 + c_{yy} + c_{x_1\eta_y}c_{yx}c_{yy} + c_{y_1\eta_y}c_{yy}^2\right)\psi_{xx} \Big)
\end{aligned} \tag{S.26c}
$$

Again, the most important insight for our discussion is that the resulting term for the conditional mean $\mathrm{E}(Y_3 \mid \eta_x = z_x, \eta_y = z_y, X_2 = x_2)$ is *not* equal to the interventional mean $\mathrm{E}(Y_3 \mid do(x_2), \eta_x = z_x, \eta_y = z_y)$ as stated in (S.39a). Using the numeric parameter values from Table S.2 one obtains $\mathrm{E}(Y_3 \mid \eta_x = z_x, \eta_y = z_y, X_2 = x_2) = -5.14z_x + 5.29z_y + 1.71x_2$ for the conditional mean and $\mathrm{E}(Y_3 \mid do(x_2), \eta_x = z_x, \eta_y = z_y) = -0.6x_2 + 14.4z_x + 23.8z_y$ for the interventional mean.

The variance of the conditional distribution $\mathrm{P}(Y_3 \mid \eta_x = z_x, \eta_y = z_y, X_2 = x_2)$ is given by:

$$
\begin{aligned}
& \mathrm{V}(Y_3 \mid \eta_x = z_x, \eta_y = z_y, X_2 = x_2) \\
& = \Big(\psi_{x_1x_1}\left(\psi_{y_1y_1}\left(c_{xx}^2 c_{yy}^4 - 2c_{xx}c_{xy}c_{yx}c_{yy}^3 + c_{xy}^2 c_{yx}^2 c_{yy}^2\right) + \psi_{yy}\left(c_{xx}^2 c_{yy}^2 + c_{xx}^2\right) + c_{yx}^2 c_{yy}^2 \psi_{xx}\right) \\
& + \psi_{x_1y_1}^2\left(-c_{xx}^2 c_{yy}^4 + 2c_{xx}c_{xy}c_{yx}c_{yy}^3 - c_{xy}^2 c_{yx}^2 c_{yy}^2\right) \\
& + \psi_{x_1y_1}\left(2c_{xx}c_{xy}\left(c_{yy}^2 + 1\right)\psi_{yy} + 2c_{yx}c_{yy}^3\psi_{xx}\right) + \psi_{y_1y_1}\left(c_{xy}^2\left(c_{yy}^2 + 1\right)\psi_{yy} + c_{yy}^4\psi_{xx}\right) \\
& + \left(c_{yy}^2 + 1\right)\psi_{xx}\psi_{yy} \Big) \cdot \frac{1}{c_{xx}^2\psi_{x_1x_1} + 2c_{xx}c_{xy}\psi_{x_1y_1} + c_{xy}^2\psi_{y_1y_1} + \psi_{xx}}
\end{aligned} \tag{S.27}
$$

Again, the most important insight for our discussion is that the resulting term for the conditional variance $\mathrm{V}(Y_3 \mid \eta_x = z_x, \eta_y = z_y, X_2 = x_2)$ is *not* equal to the interventional variance $\mathrm{V}(Y_3 \mid do(x_2), \eta_x = z_x, \eta_y = z_y)$ as stated in (S.39b). Using the numeric parameter values from Table S.2 one obtains $\mathrm{V}(Y_3 \mid \eta_x = z_x, \eta_y = z_y, X_2 = x_2) = 245.71$ for the conditional variance and $\mathrm{V}(Y_3 \mid do(x_2), \eta_x = z_x, \eta_y = z_y) = 951.43$ for the interventional variance.

[6]We use the symbols "×" and "·" interchangeably to represent standard scalar multiplication.

Formulae from the Main Text

In the previous section the formulae for the interventional and conditional distribution were derived using matrix notation. Note that the formulae in the main text contain only scalar expressions and are free of matrices and vectors. In the following two sections we link the general expressions using matrix notation and the scalar representations from the main.

Formulae from the Main Text (Homogeneous Population)

The first part of the main text treats the model for a homogeneous population. It contains formulae for the unconditional, the conditional, and the interventional distribution of the single outcome variable Y_3. First, the means and the variances of all three distributions are discussed followed by probabilities of interventional events.

The unconditional moments of $\mathbf{V}$ are derived via the reduced form of the structural equation model (Bollen, 1989). The corresponding model implied joint distribution of all variables $\mathbf{V}$ is stated in Equation (S.14). In the homogeneous model the vector $\mathbf{V}$ is given by $\mathbf{V}^\intercal = (X_1, X_2, Y_1, Y_2, X_3, Y_3, X_4, Y_4)$ of which Y_3 is the 6th entry. The unconditional mean of Y_3 is obtained by selecting the 6th entry of the model implied mean vector and the unconditional variance of Y_3 is obtained by selecting the $(6, 6)$ entry of the model implied covariance matrix.

$$\mathrm{E}(Y_3) = 0 \tag{S.28a}$$

$$\begin{aligned}\mathrm{V}(Y_3) = {} & (c_{xx}c_{yx} + c_{yx}c_{yy})^2\psi_{x_1x_1} + \left(c_{xy}c_{yx} + c_{yy}^2\right)^2 \psi_{y_1y_1} \\ & + 2(c_{xx}c_{yx} + c_{yx}c_{yy})\left(c_{xy}c_{yx} + c_{yy}^2\right)\psi_{x_1y_1} + c_{yx}^2\psi_{xx} + \left(1 + c_{yy}^2\right)\psi_{yy}\end{aligned} \tag{S.28b}$$

The unconditional variance of X_2 together with the zero-order covariance $\mathrm{COV}(X_2, Y_3)$ are not explicitly mentioned in the main text. These quantities will be used to calculate conditional means and variances later and are therefore stated below:

$$\mathrm{V}(X_2) = c_{xx}^2\psi_{x_1x_1} + c_{xy}^2\psi_{y_1y_1} + 2c_{xx}c_{xy}\psi_{x_1y_1} + \psi_{xx} \tag{S.29a}$$

$$\begin{aligned}\mathrm{COV}(X_2, Y_3) = {} & c_{xx}c_{yx}(c_{xx} + c_{yy})\psi_{x_1x_1} + c_{xy}(c_{xy}c_{yx} + c_{yy}^2)\psi_{y_1y_1} \\ & + \left(c_{xy}c_{yx}(c_{xx} + c_{yy}) + c_{xx}(c_{xy}c_{yx} + c_{yy}^2)\right)\psi_{x_1y_1} + c_{yx}\psi_{xx}\end{aligned} \tag{S.29b}$$

Note that the formulae of conditional moments presented below are derived from the established results for conditional moments of the multivariate normal distribution (Rao, 1973). Since we are dealing with a linear multivariate normal system, the conditional means and variances can be obtained via linear regression and are given by:

$$\mathrm{E}(Y_3 \mid X_2 = x_2) = \frac{\mathrm{COV}(X_2, Y_3)}{\mathrm{V}(X_2)}x_2 \tag{S.30a}$$

$$\mathrm{V}(Y_3 \mid X_2 = x_2) = \mathrm{V}(Y_3) - \frac{\mathrm{COV}(X_2, Y_3)^2}{\mathrm{V}(X_2)} \tag{S.30b}$$

The conditional mean is a function of the observed insulin level x_2 (i.e., x_2 appears on the right-hand side of Equation [S.30a]) whereas the conditional variance is functionally independent of x_2 (i.e., x_2 does not appear on the right-hand side of Equation [S.30b]). Plugging in Equations (S.29a) and (S.29b) into the right-hand side of the conditional mean equation (Equation [S.30a]) yields the following representation:

$$\begin{aligned}\mathrm{E}(Y_3 \mid X_2 = x_2) = & \left(\frac{c_{xx}c_{yx}(c_{xx} + c_{yy})\psi_{x_1x_1} + c_{xy}(c_{xy}c_{yx} + c_{yy}^2)\psi_{y_1y_1}}{c_{xx}^2\psi_{x_1x_1} + c_{xy}^2\psi_{y_1y_1} + 2c_{xx}c_{xy}\psi_{x_1y_1} + \psi_{xx}}\right. \\ & + \left.\frac{(c_{xy}c_{yx}(c_{xx} + c_{yy}) + c_{xx}(c_{xy}c_{yx} + c_{yy}^2))\psi_{x_1y_1} + c_{yx}\psi_{xx}}{c_{xx}^2\psi_{x_1x_1} + c_{xy}^2\psi_{y_1y_1} + 2c_{xx}c_{xy}\psi_{x_1y_1} + \psi_{xx}}\right)x_2\end{aligned} \tag{S.31}$$

Rearranging the scalar expressions in Equation (S.31) yields the following representation that is also displayed in the main text:

$$\begin{aligned} \mathrm{E}(Y_3 \mid X_2 = x_2) &= \frac{\mathrm{COV}(X_2, Y_3)}{\mathrm{V}(X_2)} x_2 \\ &= \left(c_{yx} + \frac{c_{xx} c_{yx} c_{yy} \psi_{x_1 x_1} + c_{xy} c_{yy}^2 \psi_{y_1 y_1} + (c_{xy} c_{yx} c_{yy} + c_{xx} c_{yy}^2) \psi_{x_1 y_1}}{c_{xx}^2 \psi_{x_1 x_1} + c_{xy}^2 \psi_{y_1 y_1} + 2 c_{xx} c_{xy} \psi_{x_1 y_1} + \psi_{xx}} \right) x_2 \end{aligned} \tag{S.32}$$

Similarly, the conditional variance could be represented as a function of the model parameters by plugging in Equations (S.29a), (S.29b) and (S.28b) into the expressions on the right-hand side of Equation (S.30b).

The interventional moments in the homogeneous model are calculated according to the following formulae:

$$\mathrm{E}(Y_3 \mid do(x_2)) = c_{yx} x_2 \tag{S.33a}$$

$$\mathrm{V}(Y_3 \mid do(x_2)) = c_{yx}^2 c_{yy}^2 \psi_{x_1 x_1} + c_{yy}^4 \psi_{y_1 y_1} + 2\, c_{yx} c_{yy}^3 \psi_{x_1 y_1} + (1 + c_{yy}^2) \psi_{yy} \tag{S.33b}$$

These formulae are an equivalent representation of the matrix expressions given in Equations (S.17). Note that the interventional mean is a function of the interventional level x_2 (i.e., x_2 appears on the right-hand side of Equation [S.33a]), whereas the interventional variance is functionally independent of x_2 (i.e., x_2 does not appear on the right-hand side of Equation [S.33b]). In other words, after performing $do(X_2 = x_2)$ the expected value of Y_3 depends on the particular value x_2 of the intervention, whereas the interventional variance does not.

Equations (S.28), (S.30), and (S.33) contain expressions for the mean and the variance of the unconditional, the conditional and the interventional distribution, respectively. Note that all three distributions are normal distributions, that is:

$$\mathrm{P}(Y_3) = \mathrm{Norm}(\, \mathrm{E}(Y_3)\, ,\, \mathrm{V}(Y_3)\,) \tag{S.34a}$$

$$\mathrm{P}(Y_3 \mid X_2 = x_2) = \mathrm{Norm}(\, \mathrm{E}(Y_3 \mid X_2 = x_2)\, ,\, \mathrm{V}(Y_3 \mid X_2 = x_2)\,) \tag{S.34b}$$

$$\mathrm{P}(Y_3 \mid do(x_2)) = \mathrm{Norm}(\, \mathrm{E}(Y_3 \mid do(x_2))\, ,\, \mathrm{V}(Y_3 \mid do(x_2))\,) \tag{S.34c}$$

Thus, probabilities can be calculated using the cumulative distribution function (cdf) of the standard normal distribution, denoted by Φ. For example, the unconditional probability that Y_3 realizes within the acceptable range $[y^{\text{lower}}, y^{\text{upper}}]$ can be calculated via:

$$\mathrm{P}(y^{\text{lower}} < Y_3 < y^{\text{upper}}) = \Phi\left(\frac{y^{\text{upper}} - \mathrm{E}(Y_3)}{\sqrt{\mathrm{V}(Y_3)}} \right) - \Phi\left(\frac{y^{\text{lower}} - \mathrm{E}(Y_3)}{\sqrt{\mathrm{V}(Y_3)}} \right) \tag{S.35}$$

Probabilities of conditional and interventional events can be calculated analogously using the conditional and interventional moments, respectively.

Figure 4 from the main text displays the interventional probability $\mathrm{P}(y^{\text{lower}} < Y_3 < y^{\text{upper}} \mid do(x_2))$ as a function of the interventional level x_2. The interventional probability is a function of x_2 because the interventional mean $\mathrm{E}(Y_3 \mid do(x_2))$ as displayed in Equation (S.33a) is a function of x_2:

$$\begin{aligned} &\mathrm{P}(y^{\text{lower}} < Y_3 < y^{\text{upper}} \mid do(x_2)) \\ =&\Phi\left(\frac{y^{\text{upper}} - c_{yx} x_2}{\sqrt{c_{yx}^2 c_{yy}^2 \psi_{x_1 x_1} + c_{yy}^4 \psi_{y_1 y_1} + 2\, c_{yx} c_{yy}^3 \psi_{x_1 y_1} + (1 + c_{yy}^2) \psi_{yy}}} \right) \\ -&\Phi\left(\frac{y^{\text{lower}} - c_{yx} x_2}{\sqrt{c_{yx}^2 c_{yy}^2 \psi_{x_1 x_1} + c_{yy}^4 \psi_{y_1 y_1} + 2\, c_{yx} c_{yy}^3 \psi_{x_1 y_1} + (1 + c_{yy}^2) \psi_{yy}}} \right) \end{aligned} \tag{S.36}$$

Note that after fixing the lower and upper bound to $y^{\text{lower}} = -40$ and $y^{\text{upper}} = 80$, respectively, and fixing the values of the model parameters to the numeric values displayed in Table S.2, the only remaining variable on the right-hand side of Equation (S.36) is x_2. In other words, the interventional probability $\text{P}(y^{\text{lower}} < Y_3 < y^{\text{upper}} \mid do(x_2))$ is a scalar-valued function of a scalar-valued argument x_2 as depicted in Figure 4 from the main text. Figure 4 from the main text also displays the conditional probability that can be expressed as a function of x_2 in a similar way.

Formulae from the Main Text (Heterogeneous Population)

The second part of the main text focuses on the model for a heterogeneous population. It contains formulae for the interventional distribution across the entire population and person-specific interventional distributions. First, the means and the variances of these distributions are discussed before average probabilities of treatment success are calculated and contrasted with person-specific probabilities of treatment success.

The unconditional moments of $\mathbf{V}$ are derived via the reduced form of the structural equation model (Bollen, 1989). The corresponding model implied joint distribution of all variables $\mathbf{V}$ is stated in Equation (S.14). In the model with random intercepts the vector $\mathbf{V}$ is given by $\mathbf{V}^\intercal = (\eta_x, \eta_y, X_1, Y_1, X_2, Y_2, X_3, Y_3, X_4, Y_4)$ of which Y_3 is the 8th entry. The unconditional mean of Y_3 is obtained by selecting the 8th entry of the model implied mean vector and the unconditional variance of Y_3 is obtained by selecting the $(8, 8)$ entry of the model implied covariance matrix (see distribution in Equation (S.18) for model implied moments).

$$\text{E}(Y_3) = 0 \tag{S.37a}$$

$$\begin{aligned}
\text{V}(Y_3) = &\left(c_{y_1\eta_x}c_{yy}^2 + c_{x_1\eta_x}c_{yx}c_{yy} + c_{x_1\eta_x}c_{xx}c_{yx} + c_{xy}c_{y_1\eta_x}c_{yx} + c_{yx}\right)^2 \psi_{\eta_x\eta_x}\\
&+ \left(c_{y_1\eta_y}c_{yy}^2 + c_{x_1\eta_y}c_{yx}c_{yy} + c_{yy} + c_{x_1\eta_y}c_{xx}c_{yx} + c_{xy}c_{y_1\eta_y}c_{yx} + 1\right)^2 \psi_{\eta_y\eta_y}\\
&+ 2\left(c_{y_1\eta_x}c_{yy}^2 + c_{x_1\eta_x}c_{yx}c_{yy} + c_{x_1\eta_x}c_{xx}c_{yx} + c_{xy}c_{y_1\eta_x}c_{yx} + c_{yx}\right)\\
&\times \left(c_{y_1\eta_y}c_{yy}^2 + c_{x_1\eta_y}c_{yx}c_{yy} + c_{yy} + c_{x_1\eta_y}c_{xx}c_{yx} + c_{xy}c_{y_1\eta_y}c_{yx} + 1\right)\psi_{\eta_x\eta_y}\\
&+ (c_{xx}c_{yx} + c_{yy}c_{yx})^2\psi_{x_1x_1} + \left(c_{yy}^2 + c_{xy}c_{yx}\right)^2 \psi_{y_1y_1}\\
&+ 2(c_{xx}c_{yx} + c_{yy}c_{yx})\left(c_{yy}^2 + c_{xy}c_{yx}\right)\psi_{x_1y_1}\\
&+ c_{yx}^2\psi_{xx} + \left(c_{yy}^2 + 1\right)\psi_{yy}
\end{aligned} \tag{S.37b}$$

The formulae for the interventional moments in the model for a heterogeneous population are given by:

$$\text{E}(Y_3 \mid do(x_2)) = c_{yx}x_2 \tag{S.38a}$$

$$\begin{aligned}
\text{V}(Y_3 \mid do(x_2)) = &(c_{y_1\eta_x}c_{yy}^2 + c_{x_1\eta_x}c_{yx}c_{yy})^2\psi_{\eta_x\eta_x}\\
&+ (c_{y_1\eta_y}c_{yy}^2 + c_{x_1\eta_y}c_{yx}c_{yy} + c_{yy} + 1)^2\psi_{\eta_y\eta_y}\\
&+ 2(c_{y_1\eta_x}c_{yy}^2 + c_{x_1\eta_x}c_{yx}c_{yy})(c_{y_1\eta_y}c_{yy}^2 + c_{x_1\eta_y}c_{yx}c_{yy} + c_{yy} + 1)\psi_{\eta_x\eta_y}\\
&+ c_{yx}^2c_{yy}^2\psi_{x_1x_1} + c_{yy}^4\psi_{y_1y_1} + 2c_{yx}c_{yy}^3\psi_{x_1y_1} + (c_{yy}^2 + 1)\psi_{yy}
\end{aligned} \tag{S.38b}$$

These formulae are an equivalent representation of the matrix expressions given in Equations (S.20a) and (S.20b). Note that the interventional mean is a function of the interventional level x_2 (i.e., x_2 appears on the right-hand side of Equation [S.33a]) whereas the interventional variance is functionally independent of x_2 (i.e., x_2 does not appear on the right-hand side of Equation [S.33b]). The interventional mean in the model for a heterogeneous population (Equation [S.38a]) is the same as in the homogeneous model (see Equation [S.33a]). The first three lines of the interventional variance in Equation

(S.38b) are related to unobserved heterogeneity; the last line is related to uncertainty that is also present in the homogeneous model (i.e., uncertainty related to initial values and error terms) and is therefore equal to the interventional variance in the homogeneous model (see Equation [S.33b]).

The person-specific interventional moments are given by:

$$\begin{aligned}&\mathrm{E}(Y_3 \mid do(x_2), \eta_x = z_x, \eta_y = z_y) &\text{(S.39a)}\\ =\,& c_{yx}x_2 + (c_{y_1\eta_x}c_{yy}^2 + c_{x_1\eta_x}c_{yx}c_{yy})z_x + (c_{y_1\eta_y}c_{yy}^2 + c_{x_1\eta_y}c_{yx}c_{yy} + c_{yy} + 1)z_y\\ &\mathrm{V}(Y_3 \mid do(x_2), \eta_x = z_x, \eta_y = z_y) &\text{(S.39b)}\\ =\,& c_{yx}^2c_{yy}^2\psi_{x_1x_1} + c_{yy}^4\psi_{y_1y_1} + 2c_{yx}c_{yy}^3\psi_{x_1y_1} + (c_{yy}^2 + 1)\psi_{yy}\end{aligned}$$

The person-specific interventional mean as displayed in (S.39a) is equivalent to the 5th entry of the mean vector displayed in Equation (S.23a). The person-specific interventional variance as displayed in Equation (S.39b) is equivalent to the $(5, 5)$th entry of the covariance matrix displayed in Equation (S.23b).

The person-specific interventional mean is a function of the interventional level x_2 and the time-invariant characteristics z_x and z_y of a person (i.e., x_2, z_x, and z_y appear on the right-hand side of Equation [S.39a]). The person-specific interventional variance is functionally independent of x_2, z_x, and z_y and equal to the interventional variance in the homogeneous model given in Equation (S.33b).

Equations (S.37), (S.38), and (S.39) contain expressions for the mean and the variance of the unconditional, the interventional and the person-specific interventional distribution, respectively. Note that all three distributions are normal distributions, that is:

$$\begin{aligned}\mathrm{P}(Y_3) &= \mathrm{Norm}(\,\mathrm{E}(Y_3)\,,\,\mathrm{V}(Y_3)\,) &\text{(S.40a)}\\ \mathrm{P}(Y_3 \mid do(x_2)) &= \mathrm{Norm}(\,\mathrm{E}(Y_3 \mid do(x_2))\,,\,\mathrm{V}(Y_3 \mid do(x_2))\,) &\text{(S.40b)}\\ \mathrm{P}(Y_3 \mid do(x_2), \eta_x = z_x, \eta_y = z_y) &= \mathrm{Norm}(\,\mathrm{E}(Y_3 \mid do(x_2), \eta_x = z_x, \eta_y = z_y)\,,\\ &\qquad \mathrm{V}(Y_3 \mid do(x_2), \eta_x = z_x, \eta_y = z_y)\,) &\text{(S.40c)}\end{aligned}$$

Thus, probabilities can be calculated using the cumulative distribution function of the standard normal distribution, denoted by Φ. For example, the unconditional probability that Y_3 realizes within the acceptable range $[y^{\text{lower}}, y^{\text{upper}}]$ can be calculated via:

$$\mathrm{P}(y^{\text{lower}} < Y_3 < y^{\text{upper}}) = \Phi\left(\frac{y^{\text{upper}} - \mathrm{E}(Y_3)}{\sqrt{\mathrm{V}(Y_3)}}\right) - \Phi\left(\frac{y^{\text{lower}} - \mathrm{E}(Y_3)}{\sqrt{\mathrm{V}(Y_3)}}\right) \qquad \text{(S.41)}$$

Interventional probabilities and person-specific interventional probabilities can be calculated analogously using the interventional and person-specific interventional moments, respectively.

Figure 7 from the main text displays the person-specific interventional probability $\mathrm{P}(y^{\text{lower}} < Y_3 < y^{\text{upper}} \mid do(x_2), \eta_x = z_x, \eta_y = z_y)$ as a function of the interventional level x_2 three different value combinations of $\eta_x = z_x$ and $\eta_y = z_y$. The person-specific interventional probability is a function of x_2 because the person-specific interventional mean $\mathrm{E}(Y_3 \mid do(x_2), \eta_x = z_x, \eta_y = z_y)$ as displayed in Equation (S.39a) is a function of

x_2:

$$
\begin{aligned}
&\mathrm{P}(y^{\text{lower}} < Y_3 < y^{\text{upper}} \mid do(x_2), \eta_x = z_x, \eta_y = z_y) \qquad \text{(S.42)} \\
=&\Phi\left(\frac{y^{\text{upper}} - (c_{yx}x_2 + (c_{y_1\eta_x}c_{yy}^2 + c_{x_1\eta_x}c_{yx}c_{yy})z_x + (c_{y_1\eta_y}c_{yy}^2 + c_{x_1\eta_y}c_{yx}c_{yy} + c_{yy} + 1)z_y)}{\sqrt{c_{yx}^2c_{yy}^2\psi_{x_1x_1} + c_{yy}^4\psi_{y_1y_1} + 2\,c_{yx}c_{yy}^3\psi_{x_1y_1} + (1 + c_{yy}^2)\psi_{yy}}}\right) \\
-&\Phi\left(\frac{y^{\text{lower}} - (c_{yx}x_2 + (c_{y_1\eta_x}c_{yy}^2 + c_{x_1\eta_x}c_{yx}c_{yy})z_x + (c_{y_1\eta_y}c_{yy}^2 + c_{x_1\eta_y}c_{yx}c_{yy} + c_{yy} + 1)z_y)}{\sqrt{c_{yx}^2c_{yy}^2\psi_{x_1x_1} + c_{yy}^4\psi_{y_1y_1} + 2\,c_{yx}c_{yy}^3\psi_{x_1y_1} + (1 + c_{yy}^2)\psi_{yy}}}\right)
\end{aligned}
$$

Note that after fixing the lower and upper bound to $y^{\text{lower}} = -40$ and $y^{\text{upper}} = 80$, respectively, fixing the person-specific characteristics to, say $\eta_x = z_x^{Amy}, \eta_y = z_y^{Amy}$, and fixing the values of the model parameters to the numeric values displayed in Table S.2, the only remaining variable on the right-hand sinde of Equation (S.36) is x_2. In other words, the interventional probability $\mathrm{P}(y^{\text{lower}} < Y_3 < y^{\text{upper}} \mid do(x_2), \eta_x = z_x, \eta_y = z_y)$ can be understood as a scalar-valued function of a scalar-valued argument x_2 as depicted in Figure 4 from the main text. Fixing the values of the person-specific characteristics to a different pair of values, say $\eta_x = z_x^{Joe}, \eta_y = z_y^{Joe}$ modifies the functional relationship between $\mathrm{P}(y^{\text{lower}} < Y_3 < y^{\text{upper}} \mid do(x_2), \eta_x = z_x, \eta_y = z_y)$ and x_2 resulting in a different curve.

Population Values for Illustration

The numeric parameter values used throughout the article are partly based on the work by Ito et al. (1998). Unfortunately, we were unable to obtain access to the raw data (which were requested from the publisher), so we used the available time series data for the non-diabetic (control) patient depicted in Figure S.1 (line with empty circles).

To model the insulin-glucose dynamics we use the following bivariate vector autore-

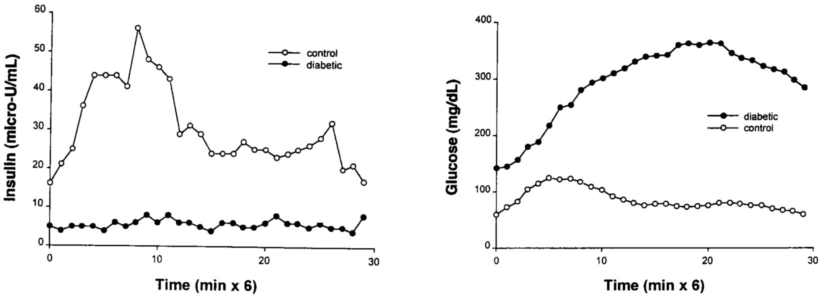

(a) time series of insulin (b) time series of glucose

Figure S.1: Time series data ($T = 31$) for a diabetic individual (line with filled circles) and a non-diabetic individual (line with empty circles) for blood insulin levels (panel a) and blood glucose levels (panel b). Figures are reprinted with permission from Ito et al. (1998, p.30).

gressive model of order one (VAR(1)):[7]

$$
\underbrace{\begin{pmatrix} X_{t+1} \\ Y_{t+1} \end{pmatrix}}_{\mathbf{Y}_{t+1}} = \underbrace{\begin{pmatrix} m_x \\ m_y \end{pmatrix}}_{\mathbf{m}} + \underbrace{\begin{pmatrix} c_{xx} & c_{xy} \\ c_{yx} & c_{yy} \end{pmatrix}}_{\mathbf{B}} \underbrace{\begin{pmatrix} X_t \\ Y_t \end{pmatrix}}_{\mathbf{Y}_t} + \underbrace{\begin{pmatrix} \varepsilon_{x,t+1} \\ \varepsilon_{y,t+1} \end{pmatrix}}_{\boldsymbol{\varepsilon}_{t+1}} \qquad t \in \mathbb{N} \qquad \text{(S.43)}
$$

[7] Note that we use the same notation for the data generating mechanisms and the the statistical model (here: a bivariate linear cross-lagged panel model).

Further we make the following assumptions:

1. innovations $\boldsymbol{\varepsilon}_t$ are independent white noise, that is, independent draws from the following normal distribution:

$$\begin{pmatrix} \varepsilon_{xt} \\ \varepsilon_{yt} \end{pmatrix} \overset{iid}{\sim} \text{Norm}\left(\begin{pmatrix} 0 \\ 0 \end{pmatrix} , \underbrace{\begin{pmatrix} \psi_{xx} & \psi_{xy} \\ \psi_{xy} & \psi_{yy} \end{pmatrix}}_{\boldsymbol{\Psi}_\varepsilon} \right) \qquad t \geq 2 \tag{S.44}$$

2. regularity conditions on $\mathbf{B}$:[8]

   (a) all eigenvalues of $\mathbf{B}$ have an absolute value less than one

   (b) the matrix $(\mathbf{I}_2 - \mathbf{B})$ is nonsingular ($\mathbf{I}_2$ is the 2×2 identity matrix)

Under assumptions (1) and (2) as stated above the solution to the stochastic difference equation displayed in Equation (S.43) is covariance-stationary and the first- and second-order moments are given by Lütkepohl (2005, p. 15):

$$\boldsymbol{\mu}_Y := \text{E}(\mathbf{Y}_t) = (\mathbf{I}_2 - \mathbf{B})^{-1}\mathbf{m} \tag{S.45a}$$

$$\boldsymbol{\Gamma}_Y(h) := \text{COV}(\mathbf{Y}_t, \mathbf{Y}_{t+h}) = \sum_{i=0}^{\infty} \mathbf{B}^{h+i}\boldsymbol{\Psi}_\varepsilon\mathbf{B}^{i\intercal} \;,\; h \in \mathbb{N}_0 \tag{S.45b}$$

We used the R-package vars (Pfaff, 2008) to fit the bivariate VAR(1) model to the data of the non-diabetic individual (lines with empty circles in panels (a) and (b) of Figure S.1), yielding the following results (see the section on "R Code for Numerical Computations" for the data set and R-code):

Estimation results and numeric values used in the illustration

	m_x	m_y	c_{xx}	c_{xy}	c_{yx}	c_{yy}	ψ_{xx}	ψ_{yy}	ψ_{xy}
estimate	-8.04	0.80	0.19	0.39	-0.58	1.20	18.95	44.02	3.00
standard error	3.73	5.69	0.15	0.08	0.23	0.12	–	–	–
value for illustration	0	0	0.05	0.40	-0.60	1.20	20	40	0

Table S.1: Note. Rows 1 and 2 of Table S.1 contain the estimation results of the VAR(1)-model for a non-diabetic individual. An empty cell (denoted by '–') indicates that the information is not routinely reported by the software. The third row of Table S.1 contains the values we use for our numerical illustration. Parameters that are set to zero in the illustration (i.e., m_x, m_y, and ψ_{xy}) do not appear in the main text.

The mean structure was not modeled in our illustration since we assumed mean-centered data. Thus, the parameters corresponding to m_x and m_y are set to zero. To keep the illustration simple, we assumed that there is no contemporaneous correlation between the innovations ε_{xt} and ε_{yt} (for $t \geq 2$) as indicated by the zero entry in the third row of the last column of Table S.1. For the remaining parameters the numeric values used to simulate data for the illustrative example were chosen to be relatively close to the estimated values (see row 1 and row 3 of Table S.1). The choice of $c_{xx} = .05$ (instead of a value closer to $\widehat{c}_{xx} = .19$) was motivated by the fact that $\widehat{c}_{xx}$ is not significantly different from zero ($\widehat{c}_{xx} = .19$, $\widehat{s.d.}(\widehat{c}_{xx}) = .15$, $\alpha = .05$). The roots of the characteristic

[8] See Bollen (1987), Hamilton (1994) and Lütkepohl (2005) for formal details and other regularity conditions.

polynomial of the estimated VAR(1)-process are real and have absolute values less than one ($\widehat{\lambda}_1 = .86$, $\widehat{\lambda}_2 = .52$), that is, the regularity conditions for $\widehat{\mathbf{B}}$ are met. To keep the illustration simple we did not allow for autocorrelations in the error terms. Consequently, we did not check for autocorrelations in the residuals of our estimation but recommend to do so in empirical applications.[9]

Inserting the numeric values from the third row of Table S.1 into the matrices $\mathbf{B}$, $\mathbf{m}$ and $\mathbf{\Psi}_\varepsilon$ from Equations (S.43) and (S.44) yields the following population quantities for our illustration:

$$\mathbf{B} = \begin{pmatrix} .05 & .40 \\ -.60 & 1.20 \end{pmatrix}, \quad \mathbf{m} = \begin{pmatrix} 0 \\ 0 \end{pmatrix}, \quad \mathbf{\Psi}_\varepsilon = \begin{pmatrix} 20 & 0 \\ 0 & 40 \end{pmatrix} \tag{S.46}$$

Again, the roots of characteristic polynomial are real and have absolute values less than one ($\lambda_1 = .93$, $\lambda_2 = .32$). The model implied covariance matrix at lag $h = 0$ can be calculated according to Equation (S.45b), yielding:

$$\mathbf{\Gamma}_Y(0) := \mathrm{COV}(\mathbf{Y}_t, \mathbf{Y}_t) = \mathrm{V}(\mathbf{Y}_t) = \sum_{i=0}^{\infty} \mathbf{B}^{0+i} \mathbf{\Psi}_\varepsilon \mathbf{B}^{i\intercal} = \begin{pmatrix} 131.77 & 254.12 \\ 254.12 & 632.94 \end{pmatrix} \tag{S.47}$$

The initial variables X_1 and Y_1 are exogenous in our model. A common practice is to assume that the initial variables are distributed according to the distribution of the stationary solution (Hamilton, 1994), yielding:

$$\begin{pmatrix} X_1 \\ Y_1 \end{pmatrix} = \begin{pmatrix} \varepsilon_{x1} \\ \varepsilon_{y1} \end{pmatrix} \sim \mathrm{Norm}\left(\begin{pmatrix} 0 \\ 0 \end{pmatrix}, \begin{pmatrix} \psi_{x_1x_1} & \psi_{x_1x_y} \\ \psi_{x_1y_1} & \psi_{y_1y_1} \end{pmatrix} \right) \tag{S.48a}$$

$$\text{with} \quad \begin{pmatrix} \psi_{x_1x_1} & \psi_{x_1x_y} \\ \psi_{x_1y_1} & \psi_{y_1y_1} \end{pmatrix} = \begin{pmatrix} 131.77 & 254.12 \\ 254.12 & 632.94 \end{pmatrix} \tag{S.48b}$$

So far we have focused on the time series ($T = 31$) of a single person ($N = 1$). In our illustration we assumed a panel design for N individuals and time series of fixed length $T = 4$. In the second part of the main text we assumed that the individuals form a heterogeneous population. This heterogeneity is is modeled via additive random intercepts η_x and η_y, for which we made the following assumptions:

$$\begin{pmatrix} \eta_x \\ \eta_y \end{pmatrix} \sim \mathrm{Norm}\left(\begin{pmatrix} 0 \\ 0 \end{pmatrix}, \begin{pmatrix} \psi_{\eta_x\eta_x} & \psi_{\eta_x\eta_y} \\ \psi_{\eta_x\eta_y} & \psi_{\eta_y\eta_y} \end{pmatrix} \right) \tag{S.49a}$$

$$\text{with} \quad \begin{pmatrix} \psi_{\eta_x\eta_x} & \psi_{\eta_x\eta_y} \\ \psi_{\eta_x\eta_y} & \psi_{\eta_y\eta_y} \end{pmatrix} = \begin{pmatrix} 5 & 2.5 \\ 2.5 & 10 \end{pmatrix} \tag{S.49b}$$

The numeric values of the variances and the covariance of the the random intercepts could *not* be estimated from the available data ($N = 1$, $T = 31$). Instead, the values of the variances were chosen to be equal to 25% of the variances of the corresponding dynamic error terms,that is $\psi_{\eta_x\eta_x} = 0.25 \cdot \psi_{xx} = 0.25 \cdot 20 = 5$ and $\psi_{\eta_y\eta_y} = 0.25 \cdot \psi_{yy} = 0.25 \cdot 40 = 10$. The value of the covariance of the random intercepts $\psi_{\eta_x\eta_y} = 2.5$ was chosen such that the resulting correlation is of medium size according to Cohen's (1988) classification.

Finally, the following values were assumed for coefficients corresponding to the directed paths from the random intercepts to the initial values: $c_{x_1\eta_x} = -4.00$, $c_{x_1\eta_y} = 8.00$, $c_{y_1\eta_x} = -12.00$ and $c_{y_1\eta_y} = 19.00$. These values correspond to the long term accumulated effects of the random intercepts on the insulin and glucose levels, which can be

[9]From a causal point of view, a serial correlation among error terms (bidirected edge between successive error terms in the DAG) is an important indicator of (omitted) time-varying confounders.

calculated according to the following formula (Lütkepohl, 2005):[10]

$$\begin{pmatrix} c_{x_1\eta_x} & c_{x_1\eta_y} \\ c_{y_1\eta_x} & c_{y_1\eta_y} \end{pmatrix} = \begin{pmatrix} 1-c_{xx} & c_{xy} \\ c_{yx} & 1-c_{yy} \end{pmatrix}^{-1} = \begin{pmatrix} \frac{1-c_{yy}}{c_{yy}c_{xx}-c_{xx}-c_{xy}c_{yx}-c_{yy}+1} & \frac{c_{xy}}{c_{yy}c_{xx}-c_{xx}-c_{xy}c_{yx}-c_{yy}+1} \\ \frac{c_{yx}}{c_{yy}c_{xx}-c_{xx}-c_{xy}c_{yx}-c_{yy}+1} & \frac{1-c_{xx}}{c_{yy}c_{xx}-c_{xx}-c_{xy}c_{yx}-c_{yy}+1} \end{pmatrix} \tag{S.50}$$

Table S.2 collects all model parameters and their corresponding numeric values. All numeric quantities presented in the main text, the Appendix to the main text, and in this online supplementary material were calculated based on these numeric parameter values.

Numeric values of parameters in the model for a heterogeneous population

	structural coefficients				variance-covariance parameters				
unobserved heterogeneity	$c_{x_1\eta_x}$ -4.00	$c_{y_1\eta_y}$ 19.00	$c_{x_1\eta_y}$ 8	$c_{y_1\eta_x}$ -12	$\psi_{\eta_x\eta_x}$ 5.00	$\psi_{\eta_y\eta_y}$ 10.00	$\psi_{\eta_x\eta_y}$ 2.50		
homogeneous part of the model	c_{xx} 0.05	c_{xy} 0.40	c_{yx} -0.60	c_{yy} 1.20	$\psi_{x_1x_1}$ 131.76	$\psi_{y_1y_1}$ 632.94	$\psi_{x_1y_1}$ 254.12	ψ_{xx} 20.00	ψ_{yy} 40.00

Table S.2: Note. The numeric values of the structural coefficients correspond to a stationary and ergodic bivariate time series for the insulin-glucose dynamics displayed in Figures 1 and 5 of the main text. The variances ($\psi_{x_1x_1}$,$\psi_{y_1y_1}$) and covariance ($\psi_{x_1y_1}$) of the initial variables correspond to the long term equilibrium values of the insulin-glucose process. The structural coefficients related to the unobserved heterogeneity (left part of row one) correspond to the accumulated long-term effects of the random intercepts on insulin and glucose levels, respectively

Computer Code

The calculations were done on Windows 10 Pro (64-bit), platform: x86_64-w64-mingw32/x64 (64-bit).

R Code for Numerical Computations

We used the following open access software: R-Studio (version 1.1.463) (RStudio Team, 2016), R (R version 3.6.1 [2019-07-05]) (R Core Team, 2019), vars (version 1.5-3) (Pfaff, 2008), and matrixcalc (version 1.0-3) (Novomestky, 2012). The following R script was used to create all reported numerical results in the main text, the Appendix to the main text, and this online supplementary material. Before running the script, please ensure that the packages vars and matrixcalc are installed on your device.

```
library(matrixcalc)
library(vars)
######################################################################
# Part I: Data generation
######################################################################
# read in data and obtain estimates of bivariate vector autoregression
ins<-c(17,21,25,37,43,43,43,40,57,48,45,43,28,31,29,24,23,23,25,24,24,22,22,21,22,23,24,26,31,18)
gluc<-c(65,75,85,105,115,125,120,123,118,110,100,85,80,75,70,75,75,70,70,73,75,80,78,75,70,70,68,65,63,60)
```

[10]The calculation is based on a set of purely mathematical assumptions (e.g., the process has an infinite past, the causal structure and the parameter values are time stable) routinely made when formally analyzing the long-term characteristics of stochastic processes.

```
d<-data.frame(ins=(ins-mean(ins)),gluc=(gluc-mean(gluc)))
VAR1<-VAR(d, p = 1, type="const")
summary(VAR1)
#################
# read in parameter values for the data generation
# and obtain matrices needed for data generation
theta_dgp<-c(0.05,0.4,-0.6,1.2,20,40)
Sigma_epsilon<-matrix(nrow=2,c(theta_dgp[5],0,0,theta_dgp[6]))
B<-matrix(nrow=2,theta_dgp[c(1,2,3,4)],byrow = T)
# check if process satisfies regularity conditions
abs(eigen(B)$values)
# compute the value of the auto-covariance function at lag zero
# assuming that the process has an infinite past
I_4<-diag(1, nrow = (4))
Gamma_0<-solve(I_4-B%x%B)%*%vec(Sigma_epsilon)
Gamma_0<-matrix(Gamma_0, nrow=2)
# add parameters for the initial values that correspond to the
# long term stable values of the system
theta_dgp<-round(c(B[1,1],B[1,2],B[2,1],B[2,2],Gamma_0[1,1],Gamma_0[2,2],Gamma_0[1,2],Sigma_epsilon[1,1],Sigma_epsilon[2,2]),2)
theta_dgp
# add variances and covariances of the person-specific
# time invariant variables; since Ito et al. (1998) contains only a single time series
# we postulate a hypothetical between-person variance:
# exx=0.25*varepsilonxx, eyy=0.25*varepsilonyy
Sigma_eta<-matrix(nrow=2, c(5,2.5,2.5,10))
is.positive.definite(Sigma_eta)
eigen(Sigma_eta)
correxey<-Sigma_eta[1,2]/(sqrt(Sigma_eta[1,1]*Sigma_eta[2,2]))
# add loadings of the person-specific time invariant variables
# onto X and Y; the loading on the initial variable is set
# equal to the long term effect
I_2<-diag(1, nrow=2)
tao<-solve(I_2-B)
# the long run cumulative effect of a unit impulse on x
# is -4; that of a unit impulse on y is 19;
theta_dgp<-round(c(tao[1,1],tao[2,2],tao[1,2],tao[2,1],B[1,1],B[1,2],B[2,1],
B[2,2],Sigma_eta[1,1],Sigma_eta[2,2],Sigma_eta[1,2],
Gamma_0[1,1],Gamma_0[2,2],Gamma_0[1,2],Sigma_epsilon[1,1],Sigma_epsilon[2,2]),2)
# create data frame for parameters
thetas<-data.frame(t(1:16))
names(thetas)<-c("cx0ex","cy0ey","cx0ey","cy0ex",
                "cxx","cxy","cyx","cyy","psiexex","psieyey",
                "psiexey","psixx","psiyy","psixy","psixr","psiyr")
thetas[2,]<-theta_dgp
View(thetas)
######################################################################
# Part II: Results for a homogeneous population
######################################################################
# Define the matrix of structural coefficients,
# the error covariance matrix and
# compute the model implied moments
k<-2 # number of processes (insulin, glucose)
T<-4 # number of time points / measurement occasions
n<-k*T # number of variables in the system
r<-2 # row number from which the theta values are exctracted
Psi_hom<-matrix(nrow=n,ncol=n,0)
Psi_hom[1,1]<-thetas$psixx[r]
Psi_hom[3,3]<-thetas$psixr[r]
Psi_hom[5,5]<-thetas$psixr[r]
Psi_hom[7,7]<-thetas$psixr[r]
Psi_hom[2,2]<-thetas$psiyy[r]
Psi_hom[4,4]<-thetas$psiyr[r]
Psi_hom[6,6]<-thetas$psiyr[r]
Psi_hom[8,8]<-thetas$psiyr[r]
Psi_hom[1,2]<-thetas$psixy[r]
Psi_hom[2,1]<-thetas$psixy[r]
Psi_hom

C_hom<-matrix(nrow=n,ncol=n,0)
C_hom[3,1]<-thetas$cxx[r]
C_hom[3,2]<-thetas$cxy[r]
C_hom[4,1]<-thetas$cyx[r]
C_hom[4,2]<-thetas$cyy[r]
C_hom[5,3]<-thetas$cxx[r]
C_hom[5,4]<-thetas$cxy[r]
C_hom[6,3]<-thetas$cyx[r]
C_hom[6,4]<-thetas$cyy[r]
C_hom[7,5]<-thetas$cxx[r]
C_hom[7,6]<-thetas$cxy[r]
C_hom[8,5]<-thetas$cyx[r]
C_hom[8,6]<-thetas$cyy[r]
C_hom
# expected values of error terms are zero
Eepsilon = rep(0,n)
# calculating the model implied covariance matrix
# and model implied mean vector of the joint distribution
I_Cinv_hom<-solve(diag(1,nrow=n)-C_hom)
Sigma_V_hom = I_Cinv_hom%*%Psi_hom%*%t(I_Cinv_hom)
E_V_hom = I_Cinv_hom%*%Eepsilon
# define the interventional values / values of the conditional
# variables one population sd of V3
x2<-round(sqrt(Sigma_V_hom[3,3]),2)
# Calculate the interventional mean and covariance matrix
# for do(x2)
e83<-c(0,0,1,0,0,0,0,0)
IN83<-diag(c(1,1,0,1,1,1,1,1))
T1_hom = solve(diag(1,nrow=n)-IN83%*%C_hom)%*%IN83
a1_hom = solve(diag(1,nrow=n)-IN83%*%C_hom)%*%e83
Edox2_hom=a1_hom*x2
```

```
Vdox2_hom = T1_hom%*%Psi_hom%*%t(T1_hom)
# Calculate the conditional mean and covariance matrix
# for X2=x2
# reorder mean vector and covariance matrix from
# 1,2,3,4,5,6 to 1,2,4,5,6,3 and obtain partitions
E_V_hom_reorder = E_V_hom[c(1,2,4,5,6,7,8,3)]
E_V_hom_reorder1 = E_V_hom_reorder[c(1,2,3,4,5,7,8)]
E_V_hom_reorder2 = E_V_hom_reorder[8]
Sigma_V_hom_reorder = Sigma_V_hom[, c(1,2,4,5,6,7,8,3)]
Sigma_V_hom_reorder = Sigma_V_hom_reorder[c(1,2,4,5,6,7,8,3), ]
# define selection matrices to obtain
# partitions of the reordered covariance matrix
e88 = c(0,0,0,0,0,0,0,1)
IS8o8 = matrix(c(1,0,0,0,0,0,0,0,
               0,1,0,0,0,0,0,0,
               0,0,1,0,0,0,0,0,
               0,0,0,1,0,0,0,0,
               0,0,0,0,1,0,0,0,
               0,0,0,0,0,1,0,0,
               0,0,0,0,0,0,1,0), ncol=7)
Sigma_V_hom_reorder22 = e88%*%Sigma_V_hom_reorder%*%e88
Sigma_V_hom_reorder11 = t(IS8o8)%*%Sigma_V_hom_reorder%*%IS8o8
Sigma_V_hom_reorder21 = t(e88)%*%Sigma_V_hom_reorder%*%IS8o8
Sigma_V_hom_reorder12 = t(IS8o8)%*%Sigma_V_hom_reorder%*%e88
Ex2_hom = E_V_hom_reorder1 + Sigma_V_hom_reorder12/as.numeric(Sigma_V_hom_reorder22)*(x2 - E_V_hom_reorder2)
Vx2_hom = Sigma_V_hom_reorder11 - Sigma_V_hom_reorder12%*%Sigma_V_hom_reorder21/as.numeric(Sigma_V_hom_reorder22)
#####################################
# Calculate unconditional, conditional and interventional probabilities
ylow<--40
yup<-80
P_unconditional<-pnorm(yup , mean=E_V_hom[6], sd=sqrt(Sigma_V_hom[6,6]))-pnorm(ylow , mean=E_V_hom[6], sd=sqrt(Sigma_V_hom[6,6]))
P_dox2<-pnorm(yup , mean=Edox2_hom[6], sd=sqrt(Vdox2_hom[6,6]))-pnorm(ylow , mean=Edox2_hom[6], sd=sqrt(Vdox2_hom[6,6]))
P_x2<-pnorm(yup , mean=Ex2_hom[5], sd=sqrt(Vx2_hom[5,5]))-pnorm(ylow , mean=Ex2_hom[5], sd=sqrt(Vx2_hom[5,5]))
######################################################################
# Part III: Results for a heterogeneous population
######################################################################
# Define the matrix of structural coefficients,
# the error covariance matrix and compute the model implied moments
l<-2 # number of random intercepts
n_het<-k*T+l # number of variables in the system
r<-2 # row number from which the theta values are exctracted
Psi_het<-matrix(nrow=n_het,ncol=n_het,0)
Psi_het[1,1]<-thetas$psiexex[r]
Psi_het[2,2]<-thetas$psieyey[r]
Psi_het[1,2]<-thetas$psiexey[r]
Psi_het[2,1]<-thetas$psiexey[r]
Psi_het[3,3]<-thetas$psixx[r]
Psi_het[4,4]<-thetas$psiyy[r]
Psi_het[3,4]<-thetas$psixy[r]
Psi_het[4,3]<-thetas$psixy[r]
Psi_het[5,5]<-thetas$psixr[r]
Psi_het[7,7]<-thetas$psixr[r]
Psi_het[9,9]<-thetas$psixr[r]
Psi_het[6,6]<-thetas$psiyr[r]
Psi_het[8,8]<-thetas$psiyr[r]
Psi_het[10,10]<-thetas$psiyr[r]

C_het<-matrix(nrow=n_het,ncol=n_het,0)
C_het[3,1]<-thetas$cx0ex[r]
C_het[3,2]<-thetas$cx0ey[r]
C_het[4,1]<-thetas$cy0ex[r]
C_het[4,2]<-thetas$cy0ey[r]
C_het[5,1]<-1
C_het[6,2]<-1
C_het[5,3]<-thetas$cxx[r]
C_het[5,4]<-thetas$cxy[r]
C_het[6,3]<-thetas$cyx[r]
C_het[6,4]<-thetas$cyy[r]
C_het[7,1]<-1
C_het[8,2]<-1
C_het[7,5]<-thetas$cxx[r]
C_het[7,6]<-thetas$cxy[r]
C_het[8,5]<-thetas$cyx[r]
C_het[8,6]<-thetas$cyy[r]
C_het[9,1]<-1
C_het[10,2]<-1
C_het[9,7]<-thetas$cxx[r]
C_het[9,8]<-thetas$cxy[r]
C_het[10,7]<-thetas$cyx[r]
C_het[10,8]<-thetas$cyy[r]
# expected values of error terms are zero
Eepsilon = rep(0,n_het)
######################################################
# calculating the model implied covariance matrix
# and model implied mean vector of the joint distribution
I_Cinv_het<-solve(diag(1,nrow=n_het)-C_het)
Sigma_V_het = I_Cinv_het%*%Psi_het%*%t(I_Cinv_het)
E_V_het = I_Cinv_het%*%Eepsilon
# define the interventional values / values of the conditional
# variables one population sd of V5
x2<-round(sqrt(Sigma_V_het[5,5]),2)
# define the values of the person-specific characteristics
# for threee prototypical individuals from the population
z_Amy<-c(-round(sqrt(diag(Sigma_V_het))[1],2),-round(sqrt(diag(Sigma_V_het))[2],2))
z_Sam<-c(round(sqrt(diag(Sigma_V_het))[1],2),round(sqrt(diag(Sigma_V_het))[2],2))
z_Joe<-c(0,0)
# in the following we calculate the person-specific values for Amy
z<-z_Amy
####################################################################################################
```

```
# Calculate the interventional mean and covariance matrix
# for do(X2=x2)
e105<-c(0,0,0,0,1,0,0,0,0,0)
IN105<-diag(c(1,1,1,1,0,1,1,1,1,1))
T1_het = solve(diag(1,nrow=n_het)-IN105%*%C_het)%*%IN105
a1_het = solve(diag(1,nrow=n_het)-IN105%*%C_het)%*%e105
Edox2_het=a1_het*x2
Vdox2_het = T1_het%*%Psi_het%*%t(T1_het)
# get the interventional distribution of all
# non-interventional variables
one10_o5<-matrix(ncol=(n_het-1), c( 1,0,0,0,0,0,0,0,0,0,
                                  0,1,0,0,0,0,0,0,0,0,
                                  0,0,1,0,0,0,0,0,0,0,
                                  0,0,0,1,0,0,0,0,0,0,
                                  0,0,0,0,0,1,0,0,0,0,
                                  0,0,0,0,0,0,1,0,0,0,
                                  0,0,0,0,0,0,0,1,0,0,
                                  0,0,0,0,0,0,0,0,1,0,
                                  0,0,0,0,0,0,0,0,0,1))
# via selection from the interventional moments
Edox2_non_het = t(one10_o5)%*%Edox2_het
Vdox2_non_het = t(one10_o5)%*%Vdox2_het%*%one10_o5
#############################################################################################
# Calculate the conditional mean and covariance matrix
# given etax=zx and etay=zy of the interventional distribution etax=zx and etay=zy
Edox2c12_re = Edox2_non_het[c(3,4,5,6,7,8,9,1,2)]
# obtain partitions of the reordered mean vector
Edox2c12_1 = Edox2c12_re[1:7]
Edox2c12_2 = Edox2c12_re[c(8,9)]
# change the oder of variabels in the interventional covariance
# matrix from 1,2,,4,5,6 to 1,2,5,6,4
Vdox2c12_re = Vdox2_non_het[, c(3,4,5,6,7,8,9,1,2)]
Vdox2c12_re = Vdox2c12_re[c(3,4,5,6,7,8,9,1,2), ]
# define selection matrices to obtain
# partitions of the reordered covariance matrix
I9c12_1 = matrix(c(1, 0, 0, 0, 0,0,0, 0, 0,
                 0, 1, 0, 0, 0,0,0, 0, 0,
                 0, 0, 1, 0, 0,0,0, 0, 0,
                 0, 0, 0, 1, 0,0,0, 0, 0,
                 0, 0, 0, 0, 1,0,0, 0, 0,
                 0, 0, 0, 0, 0,1,0, 0, 0,
                 0, 0, 0, 0, 0,0,1, 0, 0), ncol=7)
I9c12_2 = matrix(c(0,0,0,0,0,0,0,1,0,
                 0,0,0,0,0,0,0,0,1),ncol=2)
Vdox2c12_22 = t(I9c12_2)%*%Vdox2c12_re%*%I9c12_2
Vdox2c12_11 = t(I9c12_1)%*%Vdox2c12_re%*%I9c12_1
Vdox2c12_21 = t(I9c12_2)%*%Vdox2c12_re%*%I9c12_1
Vdox2c12_12 = t(I9c12_1)%*%Vdox2c12_re%*%I9c12_2
Edox2c12 = Edox2c12_1 + Vdox2c12_12%*%solve(Vdox2c12_22)%*%(z - Edox2c12_2)
Vdox2c12 = Vdox2c12_11 - Vdox2c12_12%*%solve(Vdox2c12_22)%*%Vdox2c12_21
# Calculate unconditional, conditional and interventional probabilities
P_het<-pnorm(yup , mean=E_V_het[8], sd=sqrt(Sigma_V_het[8,8]))-pnorm(ylow , mean=E_V_het[8], sd=sqrt(Sigma_V_het[8,8]))
P_dox2_het<-pnorm(yup , mean=Edox2_het[8], sd=sqrt(Vdox2_het[8,8]))-pnorm(ylow , mean=Edox2_het[8], sd=sqrt(Vdox2_het[8,8]))
P_dox2_c12_het<-pnorm(yup , mean=Edox2c12[5], sd=sqrt(Vdox2c12[5,5]))-pnorm(ylow , mean=Edox2c12[5], sd=sqrt(Vdox2c12[5,5]))
```

Mathematica Code for Algebraic Derivations

We used the following commercial software: Mathematica (version 11.3) (Wolfram Research Inc., 2018). The following Mathematica script can be used to reproduce all analytic expressions from main text, the Appendix to the main text, and this online supplementary material.

```
(*HOMOGENEOUS CASE*)
(*define model equations*)
B8 = {{0, 0, 0, 0, 0, 0, 0, 0}, {0, 0, 0, 0, 0, 0, 0, 0}, {bxx, bxy,0, 0, 0, 0, 0, 0},
     {byx, byy, 0, 0, 0, 0, 0, 0}, {0, 0, bxx, bxy,0, 0, 0, 0}, {0, 0, byx, byy, 0, 0, 0, 0},
     {0, 0, 0, 0, bxx, bxy, 0, 0}, {0, 0, 0, 0, byx, byy, 0, 0}};
B8inv = Inverse[IdentityMatrix[8] - B8];
uvar8 = {{s1, s12, 0, 0, 0, 0, 0, 0}, {s12, s2, 0, 0, 0, 0, 0, 0}, {0,0, sx, 0, 0, 0, 0, 0},
       {0, 0, 0, sy, 0, 0, 0, 0}, {0, 0, 0, 0,sx, 0, 0, 0}, {0, 0, 0, 0, 0, sy, 0, 0},
     {0, 0, 0, 0, 0, 0, sx, 0}, {0, 0, 0, 0, 0, 0, 0, sy}};
uexp8 = {{0}, {0}, {0}, {0}, {0}, {0}, {0}, {0}};
ud8 = {{u1}, {u2}, {u3}, {u4}, {u5}, {u6}, {u7}, {u8}};

(*calculate the model implied moments*)
X8exp = B8inv.uexp8;
X8var = B8inv.uvar8.Transpose[B8inv];
X8var = Collect[X8var, {s1, s2, s12, sx, sy}];

(*calculate the interventional moments*)
e83 = {{0}, {0}, {1}, {0}, {0}, {0}, {0}, {0}};
I8o3 = {{1, 0, 0, 0, 0, 0, 0, 0}, {0, 1, 0, 0, 0, 0, 0, 0}, {0, 0, 0, 0, 0, 0, 0, 0},
       {0, 0, 0, 1, 0, 0, 0, 0}, {0, 0, 0, 0, 1, 0, 0, 0}, {0, 0, 0, 0, 0, 1, 0, 0},
     {0, 0, 0, 0, 0, 0, 1, 0}, {0, 0, 0, 0, 0, 0, 0, 1}};
xint83 = x2;
T83 = Inverse[IdentityMatrix[8] - I8o3.B8].I8o3;
a83 = Inverse[IdentityMatrix[8] - I8o3.B8].e83;
X8solint3 = T83.ud8 + a83*xint83;
X8int3var = T83.uvar8.Transpose[T83];
X8int3var = Collect[X8int3var, {s1, s2, s12, sx, sy}];
```

```
X8int3exp = a83*xint83;


(*calculate the conditional moments*)
rowOrd3 = {1, 2, 4, 5, 6, 7, 8, 3};
colOrd3 = {1, 2, 4, 5, 6, 7, 8, 3};
X8varc3 = X8var[[rowOrd3, colOrd3]];
X8c3exp = X8exp[[{1, 2, 4, 5, 6, 7, 8, 3}]]
X8c3exp1 = X8c3exp[[{1, 2, 3, 4, 5, 6, 7}]]
X8c3exp2 = Extract[X8c3exp, 8]
X8c3exp2 = ArrayReshape[X8c3exp2, {}];
e8c1 = {{0}, {0}, {0}, {0}, {0}, {0}, {0}, {1}};
X8varc322 = Transpose[e8c1].X8varc3.e8c1
X8varc322 = ArrayReshape[X8varc322, {1}];
X8varc322 = UnitVector[8, 8].X8varc3.UnitVector[8, 8]
I8c1 = Transpose[{{1, 0, 0, 0, 0, 0, 0, 0}, {0, 1, 0, 0, 0, 0, 0, 0}, {0, 0, 1, 0, 0, 0, 0, 0},
                  {0, 0, 0, 1, 0, 0, 0, 0}, {0, 0, 0, 0, 1, 0, 0, 0}, {0, 0, 0, 0, 0, 1, 0, 0},
              {0, 0, 0, 0, 0, 0, 1, 0}}];
X8varc311 = Transpose[I8c1].X8varc3.I8c1;
X8varc321 = Transpose[e8c1].X8varc3.I8c1;
X8varc312 = Transpose[I8c1].X8varc3.e8c1;
c3 = x3;
muc3 = X8c3exp1 + X8varc312*(1/X8varc322)*(c3 - X8c3exp2);
muc3s = Simplify[muc3];
var8c3 = X8varc311 - X8varc312.X8varc321/X8varc322;
var8c3s = Simplify[var8c3];


(*HETEROGENEOUS CASE*)
(*define model equations*)
B10 = {{0, 0, 0, 0, 0, 0, 0, 0, 0, 0}, {0, 0, 0, 0, 0, 0, 0, 0, 0, 0},
       {bxex, bxey, 0, 0, 0, 0, 0, 0, 0, 0}, {byex, byey, 0, 0, 0, 0, 0, 0, 0, 0},
      {1, 0, bxx, bxy, 0, 0, 0, 0, 0, 0}, {0, 1, byx, byy, 0, 0, 0, 0, 0, 0},
       {1, 0, 0, 0, bxx, bxy, 0, 0, 0, 0}, {0, 1, 0, 0, byx, byy, 0, 0, 0, 0},
      {1, 0, 0, 0, 0, 0, bxx, bxy, 0, 0}, {0, 1, 0, 0, 0, 0, byx, byy, 0, 0}};
B10inv = Inverse[IdentityMatrix[10] - B10];
uvar10 = {{ex, exy, 0, 0, 0, 0, 0, 0, 0, 0}, {exy, ey, 0, 0, 0, 0, 0, 0, 0, 0},
          {0, 0, s1, s12, 0, 0, 0, 0, 0, 0}, {0, 0, s12, s2, 0, 0, 0, 0, 0, 0},
        {0, 0, 0, 0, sx, 0, 0, 0, 0, 0}, {0, 0, 0, 0, 0, sy, 0, 0, 0, 0},
        {0, 0, 0, 0, 0, 0, sx, 0, 0, 0}, {0, 0, 0, 0, 0, 0, 0, sy, 0, 0},
        {0, 0, 0, 0, 0, 0, 0, 0, sx, 0}, {0, 0, 0, 0, 0, 0, 0, 0, 0, sy}};
uexp10 = {{0}, {0}, {0}, {0}, {0}, {0}, {0}, {0}, {0}, {0}};
ud10 = {{u1}, {u2}, {u3}, {u4}, {u5}, {u6}, {u7}, {u8}, {u9}, {u10}};


(*calculate the model implied moments*)
X10exp = B10inv.uexp10
X10var = B10inv.uvar10.Transpose[B10inv];
X10var = Collect[X10var, {ex, ey, exy, s1, s2, s12, sx, sy}];

(*calculate the interventional moments*)
e105 = {{0}, {0}, {0}, {0}, {1}, {0}, {0}, {0}, {0}, {0}};
I10o5 = {{1, 0, 0, 0, 0, 0, 0, 0, 0, 0}, {0, 1, 0, 0, 0, 0, 0, 0, 0, 0},
         {0, 0, 1, 0, 0, 0, 0, 0, 0, 0}, {0, 0, 0, 1, 0, 0, 0, 0, 0, 0},
          {0, 0, 0, 0, 0, 0, 0, 0, 0, 0}, {0, 0, 0, 0, 0, 1, 0, 0, 0, 0},
       {0, 0, 0, 0, 0, 0, 1, 0, 0, 0}, {0, 0, 0, 0, 0, 0, 0, 1, 0, 0},
       {0, 0, 0, 0, 0, 0, 0, 0, 1, 0}, {0, 0, 0, 0, 0, 0, 0, 0, 0, 1}};
xint105 = x5
T105 = Inverse[IdentityMatrix[10] - I10o5.B10].I10o5;
a105 = Inverse[IdentityMatrix[10] - I10o5.B10].e105;
X10int5var = T105.uvar10.Transpose[T105];
X10int5var = Collect[X10int5var, {ex, ey, exy, s1, s2, s12, sx, sy}];
X10int5exp = a105*xint105;

I10s5 = Transpose[{{1, 0, 0, 0, 0, 0, 0, 0, 0, 0}, {0, 1, 0, 0, 0, 0, 0, 0, 0, 0},
                   {0, 0, 1, 0, 0, 0, 0, 0, 0, 0}, {0, 0, 0, 1, 0, 0, 0, 0, 0, 0},
               {0, 0, 0, 0, 0, 1, 0, 0, 0, 0}, {0, 0, 0, 0, 0, 0, 1, 0, 0, 0},
               {0, 0, 0, 0, 0, 0, 0, 1, 0, 0}, {0, 0, 0, 0, 0, 0, 0, 0, 1, 0},
               {0, 0, 0, 0, 0, 0, 0, 0, 0, 1}}];
X10int5nonvar = Transpose[I10s5].T105.uvar10.Transpose[T105].I10s5;
X10int5nonvar = Collect[X10int5nonvar, {ex, ey, exy, s1, s2, s12, sx, sy}];
X10int5nonexp = Transpose[I10s5].X10int5exp;

(*calculate persion-specific interventional moments*)

rowOrd3 = {3, 4, 5, 6, 7, 8, 9, 1, 2};
colOrd3 = {3, 4, 5, 6, 7, 8, 9, 1, 2};
X10int5nonvarc12 = X10int5nonvar[[rowOrd3, colOrd3]];
X10int5nonc12exp = X10int5nonexp[[{3, 4, 5, 6, 7, 8, 9, 1, 2}]]
X10int5nonc12exp1 = X10int5nonc12exp[[{1, 2, 3, 4, 5, 6, 7}]]
X10int5nonc12exp2 = X10int5nonc12exp[[{8, 9}]]
I10int5nonc121 = Transpose[{{1, 0, 0, 0, 0, 0, 0, 0, 0}, {0, 1, 0, 0, 0, 0, 0, 0, 0},
            {0, 0, 1, 0, 0, 0, 0, 0, 0}, {0, 0, 0, 1, 0, 0, 0, 0, 0},
         {0, 0, 0, 0, 1, 0, 0, 0, 0}, {0, 0, 0, 0, 0, 1, 0, 0, 0},
         {0, 0, 0, 0, 0, 0, 1, 0, 0}}];
I10int5nonc122 = Transpose[{{0, 0, 0, 0, 0, 0, 0, 1, 0}, {0, 0, 0, 0, 0, 0, 0, 0, 1}}];
X10int5nonvarc1222 = Transpose[I10int5nonc122].X10int5nonvarc12.I10int5nonc122;
X10int5nonvarc1211 = Transpose[I10int5nonc121].X10int5nonvarc12.I10int5nonc121;
X10int5nonvarc1221 = Transpose[I10int5nonc122].X10int5nonvarc12.I10int5nonc121;
X10int5nonvarc1212 = Transpose[I10int5nonc121].X10int5nonvarc12.I10int5nonc122;
c12 = {{z1}, {z2}};
muint5nonc12 = X10int5nonc12exp1 + X10int5nonvarc1212.Inverse[X10int5nonvarc1222].(c12 - X10int5nonc12exp2);
muint5nonc12s = Simplify[muint5nonc12];
muint5nonc12s = Collect[muint5nonc12s, {x5, z1, z2}];
var10int5nonc12 = X10int5nonvarc1211 - X10int5nonvarc1212.Inverse[X10int5nonvarc1222].X10int5nonvarc1221;
ar10int5nonc12s = Simplify[var10int5nonc12];
var10int5nonc12s = Collect[var10int5nonc12s, {ex, ey, exy, s1, s2, s12, sx, sy}];
```

References

Bollen, K. A. (1987). Total, direct, and indirect effects in structural equation models. *Sociological Methodology*, *17*, 37–69.

Bollen, K. A. (1989). *Structural equations with latent variables.* New York, NY: John Wiley & Sons.

Cohen, J. (1988). *Statistical power analysis for the behavioral sciences.* Mahwah, NJ: Lawrence Erlbaum Associates.

Gische, C., & Voelkle, M. C. (2020). Beyond the mean: A flexible framework for studying causal effects using linear models. *Manuscript submitted for publication*. Retrieved from https://www.researchgate.net/publication/335030449_Gische_Voelkle_Causal_Inference_in_Linear_Models

Hamilton, J. (1994). *Time series analysis.* Princeton, NJ: Princeton University Press.

Ito, K., Wada, T., Makimura, H., Matsuoka, A., Maruyama, H., & Saruta, T. (1998). Vector autoregressive modeling analysis of frequently sampled oral glucose tolerance test results. *The Keio Journal of Medicine*, *47*(1), 28–36.

Lütkepohl, H. (2005). *New introduction to multiple time series analysis.* Berlin/ Heidelberg: Germany: Springer-Verlag.

Novomestky, F. (2012). matrixcalc: Collection of functions for matrix calculations [Computer software manual]. Retrieved from https://CRAN.R-project.org/package=matrixcalc (R package version 1.0-3)

Pfaff, B. (2008). Var, svar and svec models: Implementation within R package vars. *Journal of Statistical Software*, *27*(4).

R Core Team. (2019). R: A language and environment for statistical computing [Computer software manual]. Vienna, Austria. Retrieved from https://www.R-project.org/

Rao, C. (1973). *Linear statistical inference and its applications* (2nd ed.). New York, NY: Wiley.

RStudio Team. (2016). Rstudio: Integrated development environment for r [Computer software manual]. Boston, MA. Retrieved from http://www.rstudio.com/

Wolfram Research Inc. (2018). *Mathematica, Version 11.3.* Champaign, IL. Retrieved from https://www.wolfram.com/mathematica
